# Immunohistochemical Characterization of the Nervous System of *Culex pipiens* (Diptera, Culicidae)

**DOI:** 10.3390/biology11010057

**Published:** 2022-01-01

**Authors:** Katharina M. Gregor, Stefanie C. Becker, Fanny Hellhammer, Wolfgang Baumgärtner, Christina Puff

**Affiliations:** 1 Department of Pathology, University of Veterinary Medicine Hannover, Bünteweg 17, Lower Saxony, 30559 Hannover, Germany; katharina.manuela.gregor@tiho-hannover.de (K.M.G.); christina.puff@tiho-hannover.de (C.P.); 2 Institute for Parasitology, University of Veterinary Medicine Hannover, Bünteweg 17, Lower Saxony, 30559 Hannover, Germany; stefanie.becker@tiho-hannover.de (S.C.B.); fanny.hellhammer@tiho-hannover.de (F.H.); 3 Research Center for Emerging Infections and Zoonoses, University of Veterinary Medicine Hannover, Bünteweg 17, Lower Saxony, 30559 Hannover, Germany

**Keywords:** *Culex pipiens* biotype *molestus*, *Drosophila melanogaster*, immunohistochemistry, invertebrate, nervous system, neurotransmitter

## Abstract

**Simple Summary:**

Arbovirus-transmitting mosquitoes pose an omnipresent threat. Therefore, insights into the underlying mechanisms of (i) mosquito behavior, (ii) species-specific behavioral traits, and (iii) behavioral changes of arbovirus-infected mosquitoes are of great interest in vector research and disease pathogenesis. Consequently, tools to enable immunohistochemical investigations of the nervous system of mosquitoes are required to further elucidate the peculiarities of neuroanatomy and neurotransmission across the spectrum of mosquito species. Accordingly, the present study aimed to provide an immunohistochemical characterization of the nervous tissue of the widespread vector *Culex pipiens* biotype *molestus* in direct comparison with the model organism *Drosophila melanogaster*. Comparative immunohistochemical assessment of selected antisera presented immunomarkers for the entire nervous tissue, for the neuropilar meshwork of axons, dendrites and synapses, and for specialized neurons and/or glial cells.

**Abstract:**

Arthropod-borne diseases represent one of the greatest infection-related threats as a result of climate change and globalization. Repeatedly, arbovirus-infected mosquitoes show behavioral changes whose underlying mechanisms are still largely unknown, but might help to develop control strategies. However, in contrast to well-characterized insects such as fruit flies, little is known about neuroanatomy and neurotransmission in mosquitoes. To overcome this limitation, the study focuses on the immunohistochemical characterization of the nervous system of *Culex pipiens* biotype *molestus* in comparison to *Drosophila melanogaster* using 13 antibodies labeling nervous tissue, neurotransmitters or neurotransmitter-related enzymes. Antibodies directed against γ-aminobutyric acid, serotonin, tyrosine-hydroxylase and glutamine synthetase were suitable for investigations in *Culex pipiens* and *Drosophila melanogaster*, albeit species-specific spatial differences were observed. Likewise, similar staining results were achieved for neuronal glycoproteins, axons, dendrites and synaptic zones in both species. Interestingly, anti-phosphosynapsin and anti-gephyrin appear to represent novel markers for synapses and glial cells, respectively. In contrast, antibodies directed against acetylcholine, choline acetyltransferase, elav and repo failed to produce a signal in *Culex pipiens* comparable to that in *Drosophila melanogaster*. In summary, present results enable a detailed investigation of the nervous system of mosquitoes, facilitating further studies of behavioral mechanisms associated with arboviruses in the course of vector research.

## 1. Introduction

Arthropod-borne diseases pose an increasing threat for many species including mammals and birds [1,2] since globalization, urbanization [2,3] and climate change [4,5] result in an enlarged vector population size and habitat [2,6]. Furthermore, a transmission of pathogens to susceptible native insect populations may occur [7,8]. Emerging arboviral diseases in Europe include Zika [9], chikungunya [10], dengue [11], Rift Valley fever [12], Usutu [13], and West Nile [14]. More than 100 arboviruses are currently circulating worldwide, capable of causing disease in mammals [15]. Similarly, a high number of insect species may act as vectors including approximately 49 mosquito species in Germany alone [16]. 

*Culex pipiens* belongs to the family Culicidae of the order Diptera. This mosquito, also known as the northern house mosquito, is native to temperate Europe and Asia, but has spread to temperate zones worldwide [17]. In Germany, *Culex pipiens* belongs to one of the most abundant mosquitoes [18]. These mosquitoes are known vectors for a variety of pathogens, such as Rift Valley fever virus [19], West Nile virus [20], and Usutu virus [21], which are harmful for animals and humans [22]. 

Although arboviruses replicate in both host and vector, no apparent detrimental effects are observed in the latter [23,24]. However, in addition to infection of the nervous system [23,25,26,27,28], there is evidence that arboviruses alter vector behavior [29]. Arbovirus-infected mosquitoes have repeatedly shown behavioral changes, including altered locomotor activity, host-seeking or feeding behavior [23,24,30,31,32,33]. These factors indicate virus-induced changes in the nervous system of vectors and/or an influence on neurotransmitter synthesis, degradation, and distribution [23,24,30,31,32]. However, the underlying mechanisms of infection-related behavioral changes are still largely unknown [31]. 

One factor contributing to the lack of knowledge represents limited information about the neuroanatomy and neurotransmission of many mosquito species, irrespective of extensive efforts to elucidate the structural and functional organization of their nervous systems [34,35,36,37,38,39,40,41]. In fact, most studies focus on *Aedes* and *Anopheles* species, although cross-species studies are highly important for understanding the underlying mechanisms of species-specific behavioral traits [40,42]. 

Therefore, the aim of the present study was an immunohistochemical characterization of the nervous system of *Culex pipiens* biotype *molestus*, including the distribution of neurotransmitters and neurotransmitter-related enzymes, as a basis for future investigations in vector research. Results obtained were compared to those of *Drosophila melanogaster* as a widely studied model organism [43]. The investigation includes markers for (1) nervous tissue such as bruchpilot, embryonic lethal visual system (elav), futsch, gephyrin, horseradish peroxidase, phosphosynapsin and reversed polarity (repo), (2) the neurotransmitters acetylcholine, γ-aminobutyric acid and serotonin, and (3) the neurotransmitter-related enzymes choline acetyltransferase, glutamine synthetase and tyrosine-hydroxylase.

## 2. Materials and Methods

### 2.1. Animal Samples and Tissue Processing

Adult female individuals of a laboratory-established *Culex pipiens* biotype *molestus* line (*Culex pipiens*; courtesy of Department of Arbovirology, Bernhard Nocht Institute for Tropical Medicine, Hamburg, Germany) and a laboratory strain of *Drosophila melanogaster* (courtesy of Jean-Luc Imler, Institut de Biologie Moléculaire et Cellulaire; Université Luis Pasteur, Strasbourg, France) were maintained at the Institute for Parasitology and the Research Center for Emerging Infections and Zoonoses, University for Veterinary Medicine, Hannover. For histological assessment, insects were anesthetized with carbon dioxide and then fixed in 10% neutrally buffered formalin for 24 h. Subsequently, specimens were embedded in paraffin wax to produce 2–4 µm thick sections in a transverse plane.

### 2.2. Selection of Antibodies and Multiple Sequence Alignment

Many of the antibodies used in the present study were originally developed for *Drosophila melanogaster* or mammalians and were in part validated by Western blot analysis. Western blot analyses were available in the literature for bruchpilot [44,45], elav [46,47], futsch [48], repo [49,50] and choline acetyltransferase [51]. To enable prediction of antibody reactivity, multiple protein sequence alignments were performed using the Clustal Omega program (https://www.ebi.ac.uk/Tools/msa/clustalo/, accessed on 16 December 2021) [52], including sequences for *Drosophila melanogaster* and different mosquito species if available. Chosen protein sequences included bruchpilot, elav, futsch, gephyrin and phosphosynapsin as well as the neurotransmitter-related enzymes choline acetyltransferase, glutamine synthetase and tyrosine-hydroxylase. The protein repo was excluded from this alignment as no equivalent protein was available for mosquitoes. The neurotransmitters acetylcholine, γ-aminobutyric acid (GABA), and serotonin, as well as the α1,3-linked fucose, which is targeted by anti-horseradish peroxidase, were not included, since they are highly conserved non-protein molecules. An overview of antibodies, including epitope, clonality, host species, dilution, epitope retrieval, and secondary antibody are listed in Table 1.

### 2.3. Histochemistry and Immunohistochemistry

Morphological characterization of head ganglia was performed in *Culex pipiens* and *Drosophila melanogaster* after determination of the sectional plane using routine hematoxylin and eosin-stained slides. Immunohistochemistry was performed as previously described [26]. Sections were deparaffinized (Roticlear, #A538.3, Carl Roth GmbH and Co. KG, Karlsruhe, Germany), rehydrated and incubated with 0.5% hydrogen peroxide (H_2_O_2_; #9681.1, Carl Roth GmbH and Co. KG, Karlsruhe, Germany) in 85% ethanol to inactivate the endogenous peroxidase. Antigen retrieval was achieved using either simmering citrate buffer (pH: 6; #3958.1, Carl Roth GmbH and Co. KG, Karlsruhe, Germany) in a microwave (800 W) for 20 min or a 0.03% solution of proteinase K (PK, #3115887001, Sigma-Aldrich Chemie GmbH, Taufkirchen, Germany) at room temperature for 10 minutes or was not performed at all. Thereafter, sections were treated with goat serum diluted 1:5 in phosphate buffered saline (PBS) to block non-specific binding sites. Incubation with primary antibodies diluted in PBS and bovine serum albumin (Albumin Fraktion V, #0163.2, Carl Roth GmbH and Co. KG, Karlsruhe, Germany) was carried out overnight at 4 °C. Negative controls received ascites fluid from non-immunized BALB/c mice (1:1000; #BL CL8100, Cedarlane^®^, biologo, Kronshagen, Germany) and rabbit normal serum (1:3000; #R4505, Sigma-Aldrich Chemie GmbH, Tauffkirchen, Germany), respectively. Sections were then incubated for 30 min at room temperature with biotinylated secondary antibodies according to the host species of the primary antibody. Goat anti-mouse IgG (GAM; 1:200; #BA-9200, VECTOR^®^, Biozol Diagnostica Vetrieb GmbH, Eching, Germany) was used for monoclonal antibodies and goat anti-rabbit IgG (GAR; 1:200; #BA-1000, VECTOR^®^, Biozol Diagnostica Vetrieb GmbH, Eching, Germany) for polyclonal antibodies. Signal amplification was performed using the avidin-biotin-peroxidase complex (#PK 6100, Vectastain elite ABC kit, Vector Laboratories, Burlingame, CA, USA) for 30 min, followed by visualization of the antigen-antibody reaction by 3,3-diaminobenzidine tetrahydrochloride (DAB; #32750 25GF, Sigma Aldrich Chemie GmbH, Taufkirchen, Germany). Finally, sections were counterstained with hematoxylin (#T865.2, Carl Roth GmbH and Co. KG, Karlsruhe, Germany). To exclude non-specific binding of secondary antibodies, ABC Vectorstain Kit^®^ and DAB, additional experiments were performed as described above, omitting the respective reagents in separate experimental runs. No cross-reactivity was observed in these controls.

### 2.4. Evaluation of Results

Assessment of the staining results was performed independently by two pathologists using a light microscope (OLYMPUS BX53; Olympus Europa SE & Co. KG, Hamburg, Germany). Characterization of the nervous system with respect to the differential expression of immunoreactivity was carried out by comparing *Culex pipiens* with the known staining properties of *Drosophila melanogaster* and classified as either positive (+) or negative (−). Images of immunohistochemical stainings were taken using the microscope BZ-9000E (Keyence Deutschland GmbH, Neu-Isenburg, Germany).

## 3. Results

### 3.1. Multiple Sequence Alignment

The in silico assay for protein homologs identified respective protein sequences for *Drosophila melanogaster* and mosquito species such as *Culex quinquefasciatus*, showing homology between 39% to 65% and 40% to 71%, respectively. The homology to *Culex pipiens* biotype *molestus* could not be assessed due to lack of available sequences for comparison. Further details of the alignments are provided in Supplementary Materials S1, along with percent sequence identity and highlighted target epitopes if provided by the manufacturer.

### 3.2. Visualization of the Nervous System

**Figure 1:**
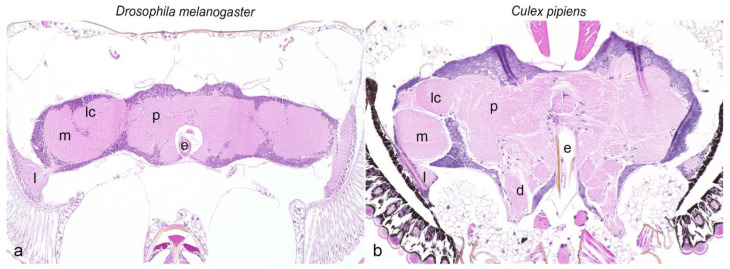
Comparative illustration of head ganglia from *Drosophila melanogaster* (**a**) and *Culex pipiens* (**b**), hematoxylin and eosin. Magnification ×20. e: esophagus; d: deutocerebrum; l: lamina; lc: lobula complex; m: medulla; p: protocerebrum.

Prior to the immunohistochemical characterization of the nervous system, hematoxylin and eosin-stained slides provided a detailed anatomical overview, which facilitated differentiation of various tissue structures, and allowed verification of the sectional plane. An anatomical overview of the head ganglia of *Drosophila melanogaster* and *Culex pipiens* is given in Figure 1 and a comparative overview of the staining results is shown in Table 2.

### 3.3. Characterization of the Nervous System

**Figure 2:**
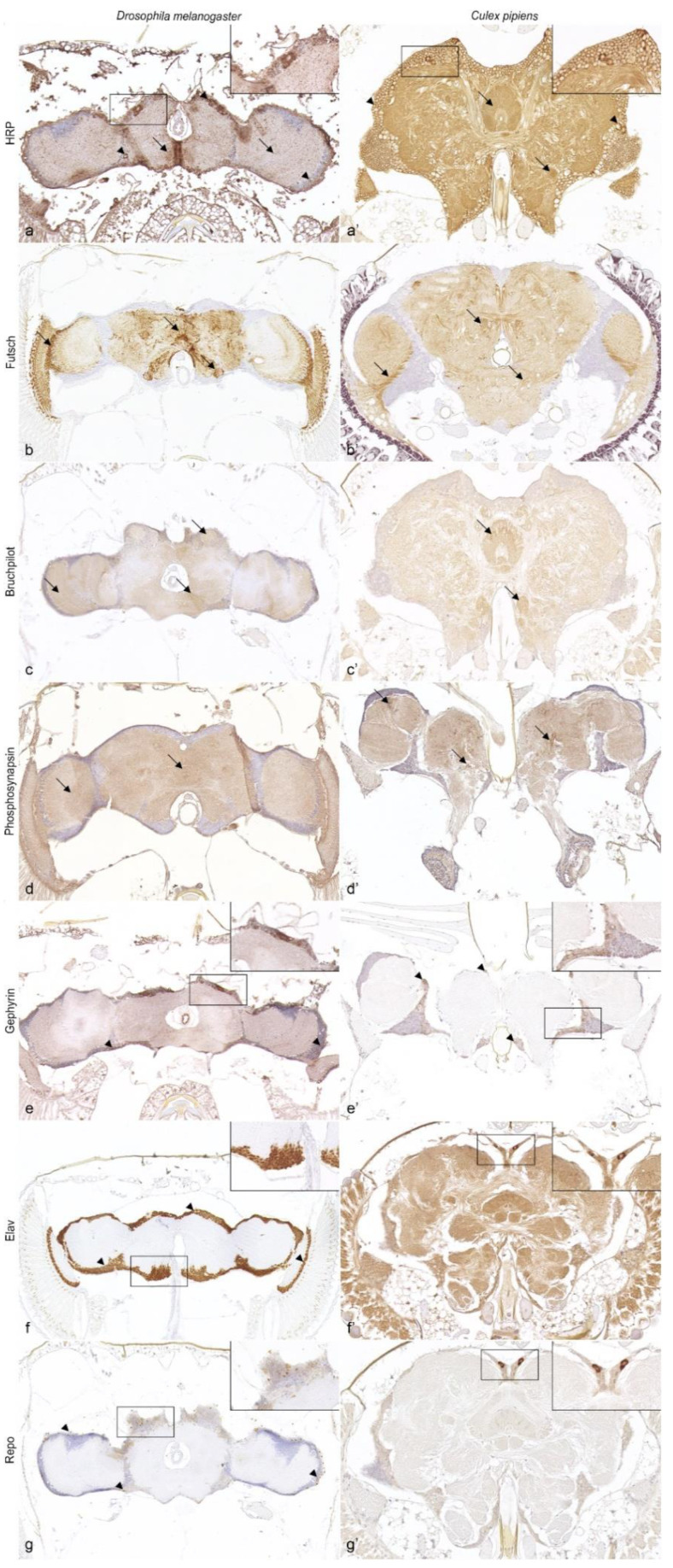
Immunoreactivity of antibodies directed against nervous tissue of *Culex pipiens* in comparison with *Drosophila melanogaster*. Anti-HRP visualizes the entire nervous tissue (cortical cells: arrowheads, neuropil: arrows) in *Drosophila melanogaster* (**a**) and *Culex pipiens* (**a’**), whereas anti-futsch (**b**,**b’**), anti-brp (**c**,**c’**) and anti-phosphosynapsin (**d**,**d’**) mark the neuropil (arrows) in both insects. Note the more defined presentation of axon tracts with anti-futsch in comparison to anti-HRP, anti-brp and anti-phosphosynapsin. Anti-gephyrin (**e**,**e’**) presents multifocally immunopositive cell bodies (arrowheads) in *Drosophila melanogaster* (**e**) and *Culex pipiens* (**e’**). While the antibody anti-repo labeled glial cells (arrowheads) in *Drosophila melanogaster* (**f**), *Culex pipiens* displayed a false positive bilateral symmetric strong cytoplasmic signal within the protocerebral cortex (**f’**). Application of anti-elav resulted in a distinctive visualization of neurons (arrowheads) within the ganglial cortex and optic lobes in *Drosophila melanogaster* (**g**). In contrast, head ganglia as well as optic lobes in *Culex pipiens* displayed a false positive bilateral symmetric strong cytoplasmic signal within the protocerebral cortex (**g’**). Magnification ×20, magnification inserts ×40. Brp: bruchpilot; elav: embryonic lethal abnormal vision; HRP: horseradish peroxidase; repo: reversed polarity homeodomain.

Application of anti-horseradish peroxidase (HRP) resulted in strong and diffuse staining of the entire nervous tissue in *Drosophila melanogaster* (Figure 2a) and *Culex pipiens* (Figure 2a’), including supra- and subesophageal ganglia as well as the optic lobes. 

Microtubules of axons and dendrites were labeled using an antibody directed against futsch in *Drosophila melanogaster* (Figure 2b) as well as *Culex pipiens* (Figure 2b’). Both insects presented an immunopositive neuropil, especially within the optic lobes, and multifocally in subcortical and neuropilar regions in the supra- and subesophageal ganglia.

Antibodies specifically targeting different synapse-associated proteins yielded an immunopositive signal in both insect species. Similar to *Drosophila melanogaster* (Figure 2c), *Culex pipiens* presented immunolabeling of the neuropil with multifocal accentuation in neuropilar regions such as the central complex or antennal glomeruli (Figure 2c’) with the marker anti-bruchpilot (brp). The marker anti-phosphosynapsin exhibited diffuse immunolabeling of the neuropil in *Drosophila melanogaster* (Figure 2d) and *Culex pipiens*, with *Culex pipiens* presenting multiple prominent immunopositive puncta within the protocerebrum as well as optic lobes (Figure 2d’). Gephyrin was expressed as a strong granular cytoplasmic immunolabeling of multiple cortical cells in the head ganglia of *Drosophila melanogaster* (Figure 2e) and *Culex pipiens* (Figure 2e’).

Application of anti-elav and anti-repo resulted in a distinct visualization of neurons and glial cells in *Drosophila melanogaster*, respectively (Figure 2f,g). In contrast, there was no specific expression pattern in the head ganglia of *Culex pipiens*. However, *Culex pipiens* displayed a strong granular false-positive bilaterally symmetric cytoplasmic signal within the protocerebral cortex with both antibodies, and strong diffuse background staining of neuropil and neurons with anti-elav (Figure 2f’,g’)

### 3.4. Neurotransmitters and Neurotransmitter-Related Enzymes

**Figure 3:**
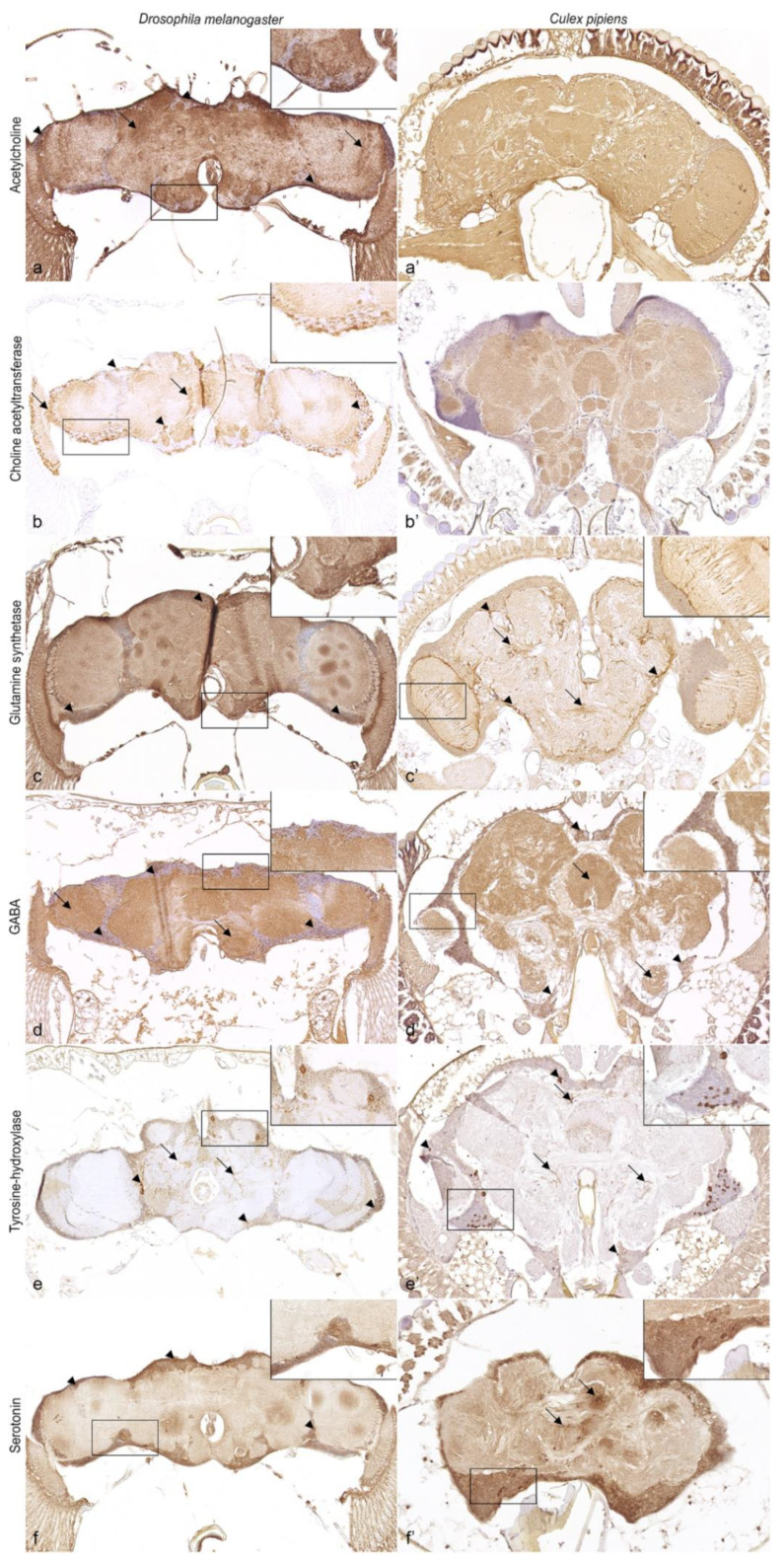
Immunoreactivity of antibodies directed against neurotransmitters and neurotransmitter-related enzymes in *Culex pipiens* in comparison to *Drosophila melanogaster*. A cell body (arrowheads) and neuropil-associated (arrows) immunolabeling was observed for ACh and ChAT in *Drosophila melanogaster* (**a**,**b**). In contrast, application of anti-ACh resulted in a diffuse immunosignal of cortex and neuropil in *Culex pipiens* (**a’**), while ChAT-immunoreactivity was restricted to the neuropil (**b’**). GS-immunoreactivity was multifocally present in cortical cell bodies in *Drosophila melanogaster* (**c**), while it was detected as a cell layer surrounding and extending short projections into the neuropil in *Culex pipiens* (**c’**). GABA-immunoreactive neurons (arrowheads) as well as innervations within the neuropil (arrows) were visible in *Drosophila melanogaster* (**d**) and *Culex pipiens* (**d’**). Note the species-specific distribution of TH-positive neurons (arrowheads) and their extensive arborization within the neuropil (arrows) in *Drosophila melanogaster* (**e**) and *Culex pipiens* (**e’**). Multifocal immunolabeling of cortical cells (arrowheads) was observed with anti-5HT in *Drosophila melanogaster* (**f**), whereas 5HT-positive neurons (arrowheads) and their arborization within the neuropil (arrows) were shown in *Culex pipiens* (**f’**). Magnification ×20, magnification insert ×40. 5HT: serotonin; ACh: acetylcholine; ChAT: choline acetyltransferase; GABA: γ-aminobutyric acid; GS: glutamine synthetase; TH: tyrosine-hydroxylase.

Application of the two antibodies targeting cholinergic neurons, anti-acetylcholine and anti-choline acetyltransferase, resulted in clear, granular, cytoplasmic labeling of numerous neurons. Moreover, these antisera visualized the innervation of neurons within the neuropil in *Drosophila melanogaster* (Figure 3a,b), with a prominent signaling pattern in the neuropil of the protocerebrum, deutocerebrum, and optic lobes. Surprisingly, the use of anti-acetylcholine resulted in diffuse immunolabeling of the cortex and neuropil in *Culex pipiens*, whereas immunostaining for the enzyme choline acetyltransferase was restricted to the neuropil (Figure 3a’,b’).

Immunoreactivity for glutamine synthetase was shown for both *Drosophila melanogaster* and *Culex pipiens*. In *Drosophila melanogaster*, immunopositive cells were multifocally visible within the cortex (Figure 3c). In contrast, *Culex pipiens* presented strong granular labeling in the cytoplasm of cortical cells surrounding and extending short projections into the neuropil (Figure 3c’).

The marker directed against GABA presented GABAergic neurons in large numbers with a strong, granular signal throughout the cortex and neuropil of the supra- and sub-esophageal ganglia in *Drosophila melanogaster* (Figure 3d) and *Culex pipiens* (Figure 3d’).

Application of the antibody targeting tyrosine-hydroxylase resulted in strong, granular, cytoplasmic labeling of specialized neurons and their extensive arborization in the neuropil in both *Drosophila melanogaster* (Figure 3e) and *Culex pipiens* (Figure 3e’).

Serotonin-immunoreactive neurons were multifocally labeled in clusters of 1–3 cells in *Drosophila melanogaster* (Figure 3f) and *Culex pipiens* (Figure 3f’) by a granular, cytoplasmic signal, with additional labeling of their arborization in *Culex pipiens*.

## 4. Discussion

Comparative immunohistochemical assessment of selected antisera presented immunomarkers for the entire nervous tissue, for the dense neuropilar meshwork of axons, dendrites and synapses, as well as for specialized neurons and/or glial cells. 

Anti-HRP is known to bind against the plant glycoprotein horseradish peroxidase, but also cross-reacts with glycoproteins, such as the α1,3-fucosylated N-glycan, expressed by nervous tissue in Ecdysozoa [61]. In *Culex pipiens* and *Drosophila melanogaster*, anti-HRP visualized the entire nervous tissue. This is in accordance with previous studies demonstrating this labeling in whole mount specimens of *Drosophila melanogaster*, *Aedes aegypti* and *Anopheles gambiae* [36,55,61]. 

Similar to *Drosophila melanogaster*, anti-futsch and anti-brp were expressed in the neuropil of *Culex pipiens*. While immunolabeling for bruchpilot in *Culex pipiens* seemed more likely with a homology of approximately 65% in the closely related *Culex quinquefasciatus*, the distinct immunolabeling for anti-futsch with a homology of only 40% was rather surprising. Nonetheless, these results are consistent with earlier studies in other mosquito species [36], rendering those markers also suitable for *Culex pipiens*. Synapsins are highly conserved synaptic vesicle-associated proteins that play a crucial role in neurotransmission [62]. The marker anti-phosphosynapsin is directed against the human phosphorylated protein synapsin 1, which is 39% and 40% homologous with *Drosophila melanogaster* and Culicinae mosquitoes, respectively. In the present study, the neuropil was labeled with anti-phosphosynapsin in both dipterans, comparable to immunolabeling with other synapsin markers in similar studies [36]. Thus, anti-phosphosynapsin likely represents a novel antibody for the study of synapses in insects. In summary, the complex organization of axon and dendrite bundles as well as synapses within the neuropil can be visualized in *Culex pipiens* and *Drosophila melanogaster* with the antibodies used.

The antibody targeting human gephyrin shares homology with an analogous protein in *Drosophila melanogaster* and Culicinae mosquitoes of approximately 39% and 41%, respectively. Application of anti-gephyrin resulted in immunostaining in *Drosophila melanogaster* and *Culex pipiens* comparable to that observed in mammals [63,64], indicating a neural function of gephyrin in insects. On the one hand, this protein is involved in the biosynthesis of the molybdenum cofactor (Moco) in eukaryotes, which also takes place in glial cells of the nervous system [54,64]. Consequently, anti-gephyrin might represent an interesting candidate to investigate Moco synthesis or allow visualization of a subset of glial cells in Diptera. However, this observation should be interpreted with caution and requires further investigation, as no information is yet available on the reactivity of this protein in the nervous system of insects. On the other hand, gephyrin is an important scaffolding protein at inhibitory postsynaptic sites by connecting glycinergic and GABAergic receptors to the cytoskeleton and is thus indirectly responsible for the strength of inhibitory neurotransmission [65]. However, no corresponding immunostaining in the form of multiple puncta of <1 µm [63] was detected in the neuropil of insect species examined. Therefore, anti-gephyrin does not appear to be suitable for the study of glycinergic or GABAergic postsynaptic sites and thus inhibitory neurotransmission in insects. 

Investigated monoclonal antibodies directed against the elav protein localized in neuronal nuclei [53] and the homeoprotein repo in the nuclei of glial cells [50], showed a clear signal in *Drosophila melanogaster*, but not in *Culex pipiens*. This observation renders these antibodies non-specific for investigations with *Culex pipiens*, despite homology for elav between *Drosophila melanogaster* and Culicinae mosquitoes of 70%. However, cross-reactivity for polyclonal elav and repo markers has recently been demonstrated in mosquitoes [36,38]. Therefore, the lack of adequate cross-reactivity is probably attributable to the binding of monoclonal antibodies to small epitopes with few amino acids, whereas polyclonal antibodies have a broad affinity for isoforms of target proteins [66]. Interestingly, the present results indicate that glutamine synthetase and gephyrin may represent valid alternatives to label subsets of glial cells in the nervous system of *Culex pipiens*.

Behavioral changes following a stimulus or a viral infection are likely to be based on the species-specific distribution of specialized neurons and the associated distribution of various neurotransmitters or neurotransmitter-related enzymes [36,40,42]. In the present study, the antibody for the neurotransmitter-related enzyme glutamine synthetase yielded a clear immunopositive signal in cell bodies of the nervous system in *Drosophila melanogaster* and *Culex pipiens*, similar to the immunoreactivity of earlier studies [36,67]. Glutamine synthetase is found in both vertebrates and invertebrates [36,67,68], with *Drosophila melanogaster* and Culicinae mosquitoes showing 65% and up to 70% homology with the human analog, respectively. This enzyme is responsible for the simultaneous metabolization of the excitatory neurotransmitter glutamate and ammonia to glutamine [69,70]. In contrast to vertebrates, glutamate was believed to be less abundant in the central nervous system and of higher significance in the peripheral nervous system [71,72,73,74]. Nevertheless, this neurotransmitter is still involved in numerous processes in the central nervous system [74,75,76,77,78] and is even reported to act as an inhibitory transmitter in the antennal lobe [79]. Furthermore, glutamine synthetase is indirectly involved in the synthesis of the inhibitory neurotransmitter GABA [80]. Thus, glutamine synthetase represents an attractive candidate for the investigation of glutamate and GABA. Interestingly, the expression of glutamate-related genes was increased in arbovirus-infected mosquitoes, suggesting altered synthesis and/or distribution, in which the involvement of glutamine synthetase cannot be excluded [23]. Finally, the presence of glutamine synthetase in glial cells [36,67,68,69] also allows visualization and thus targeted studies of these cells and their interaction in the nervous system of *Culex pipiens*.

GABA is an important inhibitory neurotransmitter involved in multiple processes within the peripheral and central nervous system [72,81,82,83]. Accordingly, GABAergic neurons were labeled in large numbers in all neuropilar regions in both *Drosophila melanogaster* and *Culex pipiens*. Results obtained were similar to previous investigations in *Drosophila melanogaster* [74,77,83,84] and other insects [75,77,82,85,86,87,88,89,90,91]. These results are highly interesting since GABA is involved in locomotor activity [23,30], regulation of the circadian clock [92], olfaction, and olfactory learning [83,93,94,95,96]. In particular, the significant role of GABA in olfaction is of interest in vector research considering that it is important for mosquito behavior [39,97,98]. Interestingly, behavioral changes related to olfaction [23,31,32] and locomotion [23,30] have already been described in arbovirus-infected mosquitoes, for which an influence on GABA is possible. Furthermore, GABA has been reported to facilitate arboviral infections of mosquitoes by modulating the gut antiviral immunity [99], denoting this antibody as a valuable tool for behavior and pathogenetic studies.

Tyrosine-hydroxylase and serotonin were both demonstrable in specialized neurons in *Culex pipiens*. As an important, rate-limiting enzyme in the synthesis of dopamine, tyrosine-hydroxylase is located in dopaminergic neurons. Therefore, immunolabeling of tyrosine-hydroxylase corresponds to the presence of dopamine [100]. The protein sequence of *Rattus norvegicus* targeted with anti-tyrosine-hydroxylase is approximately 50% homologous with *Drosophila melanogaster* and Culicinae mosquitoes. Nevertheless, and similar to previous reports, specifically labeled dopaminergic neurons were shown in both *Drosophila melanogaster* and *Culex pipiens* [40,101,102,103]. Since dopamine is involved in many behavioral patterns including learning, olfaction, and locomotion [98,100,104,105,106,107], which are reported to be altered in arbovirus-infected mosquitoes [23,30,31,32], the antibody used is advantageous for investigating neurotransmission and thus behavioral alterations among mosquito species and in pathogenetic studies.

In the present study, serotonin expression was demonstrated in *Drosophila melanogaster* and *Culex pipiens*, which is consistent with the results of former investigations [36,108,109]. Analysis of serotonergic neurons represents an interesting approach in arbovirus-related research, since serotonin is involved in aggression, feeding behavior and regulation of salivary gland secretions of mosquitoes [100,110]. A change of these behavioral traits has been observed in arbovirus-infected mosquitoes [24,32,33], which raises the presumption that this could arise from changes in the neurophysiology of 5HT, as has been postulated for La Crosse virus infections in *Aedes triseriatus* [32].

Acetylcholine is the leading excitatory neurotransmitter in the nervous system of insects and is particularly abundant in specialized neurons within the insect visual system [74,106]. Two antisera were used to localize cholinergic neurons in the nervous system of *Culex pipiens*. The first antibody was directed against the acetylcholine molecule itself. The second marker targeted choline acetyltransferase, an enzyme essential for the synthesis of acetylcholine [72] that shows a homology between *Drosophila melanogaster* and Culicinae mosquitoes of up to 71%. Surprisingly, the reactivity for both antibodies differed immensely between *Drosophila melanogaster* and *Culex pipiens*. Consistent with previous studies, both markers yielded a clear, immunopositive signal for cholinergic neurons in *Drosophila melanogaster*, illustrating the widespread expression in head ganglia [74,77,111,112,113,114]. In contrast, *Culex pipiens* displayed an unexpected immunoreactivity. The observed diffuse distribution of acetylcholine-positive neurons in the head ganglia along with immunoreactivity for choline acetyltransferase restricted to the neuropil would be an aberrant observation that has not been described to this extent in any other insect [58,74,77,111,114,115,116,117,118]. Similar results were described only in one study of the locust *Schistocerca gregaria*, where choline acetyltransferase immunoreactivity was predominantly restricted to sensory neuropil and only occasionally associated with cell bodies in the nervous system [117]. Accordingly, there are two possibilities that could explain this unexpected reactivity of acetylcholine and choline acetyltransferase. Either (1) acetylcholine plays a much larger, more complex or different role in mosquitoes than expected; or (2) both antibodies are not specific for either epitope in formalin-fixed and paraffin-embedded mosquitoes. The significance of this observation remains to be investigated.

## 5. Conclusions

The present study provides a comparative immunohistochemical characterization of the nervous system of *Culex pipiens* and the model organism *Drosophila melanogaster*. All antisera-tested labeling neural structures, neurotransmitters or neurotransmitter-related enzymes, were suitable for investigations in *Drosophila melanogaster*. Interestingly, most antibodies also proved valuable for immunolabeling in *Culex pipiens*.

Taken together, most of the investigated antibodies are suitable for subsequent analyses in *Culex pipiens* and facilitate further cross-species studies of neuroanatomy and neurotransmission in mosquitoes. This provides new possibilities in uncovering the underlying mechanisms of learning, memory, and mosquito behavior. Such knowledge might allow the observed behavioral changes in arbovirus-infected mosquitoes to be elucidated and could further implement the development of new countermeasures against arbovirus-transmitting vectors. In conclusion, this study presents a promising basis for further investigations in the context of vector research and disease pathogenesis.

## Figures and Tables

**Table 1:** Overview of antibodies used for characterization of the nervous system of *Culex pipiens* biotype *molestus* and *Drosophila melanogaster* including epitope, clonality, host species, dilution, epitope retrieval and secondary antibody.

Primary Antibody	Epitope	Clonality/Host Species	Dilution		Epitope Retrieval	Secondary Antibody	Source	Reference
			*Culex*	*Drosophila*				
Nervous Tissue								
Brp	Presynaptic active zone assembly protein	mc, mouse	1:50	1:50	heated citrate buffer *	GAM	# nc82, DSHB	[45]
Elav	Neuronal protein	mc, mouse	-	1:50,000	heated citrate buffer *	GAM	# 9F8A9, DSHB	[53]
Futsch	Microtubule-associated protein	mc, mouse	1:800	1:1600	none	GAM	# 22C10, DSHB	[48]
Gephyrin	Postsynaptic neuronal assembly protein, glial cells	pc, rabbit	1:16,000	1:4000	heated citrate buffer *	GAR	# PA5-29036, Thermo Fisher	[54]
HRP	Fucosylated N-glycans	pc, rabbit	1:25,000	1:20,000	heated citrate buffer *	GAR	# 323-005-021, Jackson Immunoresearch	[55,56]
Phosphosynapsin	Synapsin 1	pc, rabbit	1:50	1:50	PK ^#^	GAR	# PA5-38528, Thermo Fisher	[57]
Repo	Glial homeoprotein	mc, mouse	-	1:1600	heated citrate buffer *	GAM	# 8D12, DSHB	[50]
Neurotransmitters								
ACh	Acetylcholine	pc, rabbit	-	1:100	PK ^#^	GAR	# AB5522, Merck Millipore	[58]
GABA	γ-aminobutyric acid	pc, rabbit	1:6000	1:3000	none	GAR	# A2052, Sigma-Aldrich	[59]
5HT	Serotonin	pc, rabbit	1:60,000	1:6000	heated citrate buffer *	GAR	# S5545, Sigma-Aldrich	[59]
Neurotransmitter-Related Enzymes								
ChAT	Choline acetyltransferase	mc, mouse	-	1:1600	heated citrate buffer *	GAM	# ChAT4B1, DSHB	[60]
GS	Glutamine synthetase	pc, rabbit	1:8000	1:2000	heated citrate buffer *	GAR	# PA5-28940, Thermo Fisher	[36]
TH	Tyrosine-hydroxylase	mc, mouse	1:80	1:80	heated citrate buffer *	GAM	# 22941, Immunostar	[40]

-: no specific reaction; *: 20 min, microwave, 800 Watt; ^#^: 0.03% solution of proteinase K, 10 min; 5HT: serotonin; ACh: acetylcholine; Brp: bruchpilot; ChAT: choline acetyltransferase; *Culex*: *Culex pipiens* biotype *molestus*; *Drosophila*: *Drosophila melanogaster*; DSHB: Developmental Studies Hybridroma Bank; Elav: embryonic lethal abnormal vision; GABA: γ-aminobutyric acid; GAM: goat anti-mouse; GAR: goat anti-rabbit; GS: glutamine synthetase; HRP: horseradish peroxidase; mc: monoclonal; pc: polyclonal; repo: reversed polarity homeodomain; TH: tyrosine-hydroxylase.

**Table 2:** Immunoreactivity of tested antibodies in *Culex pipiens* biotype *molestus* compared to *Drosophila melanogaster*.

Primary Antibody Specificity	*Drosophila*	*Culex*
Neural Tissue		
Brp	+	+
Elav	+	− *
Futsch	+	+
Gephyrin	+	+
HRP	+	+
Phosphosynapsin	+	+
Repo	+	− *
Neurotransmitters		
5HT	+	+
ACh	+	− *
GABA	+	+
Neurotransmitter-Related Enzymes		
ChAT	+	− *
GS	+	+
TH	+	+

+: positive reaction; −: no reaction; *: false positive labeling; 5HT: serotonin; ACh: acetylcholine; Brp: bruchpilot; ChAT: choline acetyltransferase; *Culex*: *Culex pipiens* biotype *molestus*; *Drosophila*: *Drosophila melanogaster*; Elav: embryonic lethal abnormal vision; GABA: γ-aminobutyric acid; GS: glutamine synthetase; HRP: horseradish peroxidase; Repo: reversed polarity homeodomain; TH: tyrosine-hydroxylase.

## Data Availability

All data supporting the findings of this study are included within the main document and are available upon reasonable request.

## Supplementary Materials

The following supporting information can be downloaded at: https://www.mdpi.com/article/10.3390/biology11010057/s1, Supplementary Materials S1: Interspecies multiple sequence alignment of target proteins.
