# Supplementary material for: Immunohistochemical Characterization of the Nervous System of Culex pipiens (Diptera, Culicidae)
Source: Biology (Basel). 2022 Jan 1;11(1):57. doi: 10.3390/biology11010057 (PMC8772823; doi:10.3390/biology11010057)
Supplement: Supplementary file 1 [file biology-11-00057-s001.zip › biology-1498609-supplementary.pdf]

biology

MDPI

Research article

Immunohistochemical characterization of the nervous system of *Culex pipiens* (Diptera, Culicidae)

Katharina M. Gregor[1,2], Stefanie Becker[2], Fanny Hellhammer[2], Wolfgang Baumgärtner[1,*], Christina Puff[1]

[1] Department of Pathology, University of Veterinary Medicine Hannover, Bünteweg 17, Lower Saxony, 30559 Hannover, Germany
[2] Institute for Parasitology and Research Center for Emerging Infections and Zoonoses (RIZ), University of Veterinary Medicine Hannover, Bünteweg 17, Lower Saxony, 30559 Hannover, Germany
* Correspondence: Wolfgang.Baumgaertner@tiho-hannover.de, Phone: 0049 511 953 8620, Fax: 0049 511 953 8675

Supplementary material

S1. Interspecies multiple sequence alignment of the target epitope of antibodies used

S1.1 Bruchpilot (Gene ID: 35977)

S1.2 Elav (Gene ID: 31000)

S1.3 Futsch (Gene ID: 6724864)

S1.4 Gephyrin (Gene ID: 10243)

S1.5 Phosphosynapsin (Gene ID: 6853)

S1.6 Choline acetyltransferase (Gene ID: 42249)

S1.7 Glutamine synthetase (Gene ID: 2752)

S1.8 Tyrosine-hydroxylase (Gene ID: 25085)

The *in silico* assay for protein homologs identified respective protein sequences for *Drosophila melanogaster* and mosquito species such as *Culex quinquefasciatus*, showing homology between 39 to 65 % and 40 to 70 %, respectively. The homology to *Culex pipiens* biotype *molestus* could not be assessed due to lack of available sequences for comparison. The protein sequence alignments for each protein are shown below and further comprise percentage identity matrices for the respective protein. The epitope of interest is highlighted in yellow if provided by the manufacturer.

Sequence identities were marked underneath the protein alignment as follows:
* (asterisk): indicates single fully conserved amino acid,
. (period): indicates strongly similar amino acids between groups,
: (colon): indicates weakly similar amino acids between groups.

S1.1. Multiple sequence alignment for bruchpilot (Gene ID: 35977)

Drosophila melanogaster; AFH07983.1 msrddynpvtssgvrspgrvrrlqelptvdrspsrdygaprgsplamgspyyrdmdepts 60
Culex quinquefasciatus; EDS45873.1 -- 0
Anopheles darlingi; ETN64951.1 -- 0

Drosophila melanogaster; AFH07983.1 pagaghhrsrsasrppmahamdyprtryqsldrgglvdphdrefipireprdrsrdrsle 120
Culex quinquefasciatus; EDS45873.1 -- 0
Anopheles darlingi; ETN64951.1 -- 0

Drosophila melanogaster; AFH07983.1 rglyledelygrsarqspsamggyntgmgptsdraylgdlqhqntdlqrelgnlkrelel 180
Culex quinquefasciatus; EDS45873.1 -- 0
Anopheles darlingi; ETN64951.1 -- 0

Drosophila melanogaster; AFH07983.1 tnqklgssmhsiktfwspelkkeralrkeesakyslindqlkllstenqkqamlvrqlee 240
Culex quinquefasciatus; EDS45873.1 -- 0
Anopheles darlingi; ETN64951.1 -- 0

Drosophila melanogaster; AFH07983.1 elrlrmrqpnlemqqqmeaiyaendhlqreisilretikdlecrvetqkqtliardesik 300
Culex quinquefasciatus; EDS45873.1 -- 0
Anopheles darlingi; ETN64951.1 -- 0

Drosophila melanogaster; AFH07983.1	kllemlqakgmgkeeerqmfqqmqamaqkqmynnytgasgssppplpdiigqyygpgslgp	360
Culex quinquefasciatus; EDS45873.1	--	0
Anopheles darlingi; ETN64951.1	--marfyssnfh--	10
Drosophila melanogaster; AFH07983.1	gplsygsgngygppgydlygpststaaggyglydpqiygpcqqtspirrrrysiaglpsa	420
Culex quinquefasciatus; EDS45873.1	--	0
Anopheles darlingi; ETN64951.1	-----------napaskf---------------------ldvrsgrlparrysisgsvpl	38
Drosophila melanogaster; AFH07983.1	tmyndvygyp-applqqtsssnivnllneahksisrssqilnltnqarqlgqlvttplgg	479
Culex quinquefasciatus; EDS45873.1	--	0
Anopheles darlingi; ETN64951.1	ssmvdycnlsenaanlykhsanltnllnettkslsrssnilnmrcvppst---vthtlp-	94
Drosophila melanogaster; AFH07983.1	ggvgmglgrvvssyptlhpgdtsppylndnlmqmstsfssqpnmyfqpqaqvqqvlptql	539
Culex quinquefasciatus; EDS45873.1	--	0
Anopheles darlingi; ETN64951.1	gaigsnnngnscpltalfppsssq-----------------------------pvpppqs	125
Drosophila melanogaster; AFH07983.1	taaqsaaaaaarlrpaysvtnlvssypttaagygckyggygnpyqsdlgygnplapfqq	599
Culex quinquefasciatus; EDS45873.1	--	0
Anopheles darlingi; ETN64951.1	tip-ssagsvfdpspsisler-iaasaastvmgftcsypnagssaklpsaidn-------	176

Drosophila melanogaster; AFH07983.1 rqlmahqslhasnpaisqyyqnqgaatgtgasaaalaynlqqqfagtqhygggsqgpplg 659

Culex quinquefasciatus; EDS45873.1 -- 0

Anopheles darlingi; ETN64951.1 -------------ssnlsyynhl------------------------qh----------- 188

Drosophila melanogaster; AFH07983.1 hshiqasvqqqmhpqyqyhv-hqhqnhlqthplaalantsgpfggtlassardyvdglhh 718

Culex quinquefasciatus; EDS45873.1 --------------------------------m-v----ktfwndyt--------dtl-- 13

Anopheles darlingi; ETN64951.1 ---mqp----nvqpsqqltlqhqhqp-----pe-a----lhhfggyhp--sneyllsk-- 227

. :..

Drosophila melanogaster; AFH07983.1 aahshthphshphhsshhshhtayphshshhqqqqphqahhhhhlpyskldld-ypklpq 777

Culex quinquefasciatus; EDS45873.1 -----------p---------------------------------lr--krligvqipa- 26

Anopheles darlingi; ETN64951.1 -----------p---------------------------------iysnttlsgttnpfs 243

* *

Drosophila melanogaster; AFH07983.1 ehkrqldefrleiqrrdqeilamaakmktleeqhqdyqrhiavlkeslcakeehynmlqt 837

Culex quinquefasciatus; EDS45873.1 ---------------rt----nttgdlfpsgid--------------------------c 41

Anopheles darlingi; ETN64951.1 sqthslddfrmeisrrdqeimamaakmktleeq--------------------------h 277

* :..: :

Drosophila melanogaster; AFH07983.1 dveemrarleeknrliekktqgtlqtvqernrltseltelkdhmdikdrkisvlqrkien 897

Culex quinquefasciatus; EDS45873.1 lveelrnrleeknrmiekktqsamqsvqdknrvqteltelkehmdikdrkinvlqrkien 101

Anopheles darlingi; ETN64951.1 qveelrnrledknrmiekktqgtmltvqeknrlqteltelkehmdikdrkinvlqrkidn 337

:* ***::******.:: :**::**: :******:*********.******:*

Drosophila melanogaster; AFH07983.1	ledllkekdnqvdmararlsamqahhsssegaltsleeaigdkekqmaqlrdqrdraehe	957
Culex quinquefasciatus; EDS45873.1	ledllkekdnqvdmararlsamqahhcssegalssleeaigdkekqmqqlrdqrdraege	161
Anopheles darlingi; ETN64951.1	ledllkekdnqvdmararlsamqahhcssegaltsleeaigdkekqmqqlrdqrdraeae	397
	*************************.******:************* ********** *	
Drosophila melanogaster; AFH07983.1	kqeerdlherevadykiklraaeseveklqtrleravtererleikleasqselgkskae	1017
Culex quinquefasciatus; EDS45873.1	kkeerelherelaeykmklhtldseveklt srlhralnekdrlesklecsqselgkskae	221
Anopheles darlingi; ETN64951.1	kkeerelherelaefkmklhtldseveklttrlhrslaekdrleaklessqselgkskae	457
	*:***:*****:*::*:**:: :****** :**.*:: *::*** ***.***********	
Drosophila melanogaster; AFH07983.1	lekatcemgrssadwestkqriarlelenerlkhdlersqnvqklmfetgkisttfgrtt	1077
Culex quinquefasciatus; EDS45873.1	ldkaatdvgrsgsdwenakqrisrlelenerlradlersq-------------ttfgrst	268
Anopheles darlingi; ETN64951.1	ldkaatdvgrsgadwehakqrmsrlelenerlrnelersq-------------t------	498
	*:**: ::***.:*** :***::*********: :***** *	
Drosophila melanogaster; AFH07983.1	mttsqeldraqeradkasaelrrtqaelrvtqsdaerareeaaalqekleksqgevyrlk	1137
Culex quinquefasciatus; EDS45873.1	lstsqeldraqeradktsaelrrtqaelrvtqsdaerargeaaalqekleksqgevyrlk	328
Anopheles darlingi; ETN64951.1	--------------------------------daersraeaaalqekleksqgevyrlk	525
	****:* ********************	
Drosophila melanogaster; AFH07983.1	aklenaqgeqeslrqelekaqsgvsrihadrdrafsevekikeemertqatlgksqlqhe	1197
Culex quinquefasciatus; EDS45873.1	aklenaqgeqesikqemersqtgiqrivserdkayaeldkireelersqatlgksqlqqe	388
Anopheles darlingi; ETN64951.1	aklenaqgeqeslkqemergqagiqri------ayaelekireemertqatlgksqltqe	579
	************::**:*: *:*: ** *::*::**:**:**:********* :*	

Drosophila melanogaster; AFH07983.1	klqnsldkaqnevdhlqdkldkactenrrlvlekekltydydnlqsqldkalgqaarmqk	1257
Culex quinquefasciatus; EDS45873.1	kiqnaldkaqnevdhlqekldksvgevrrlqlekekinyefeniqsqldkqlgqssrvqk	448
Anopheles darlingi; ETN64951.1	klqnsldkaqndvdhlqekldksvgeirrlqlekekinyefeniqsqldkslgqssriqk	639
	*:**:******:*****:****: * *** *****:. *.:.*:****** ***:.*:**	
Drosophila melanogaster; AFH07983.1	eretlsldtdrireklektqvqlgriqkerdqfsdeletlkersesaqtllmkaardrea	1317
Culex quinquefasciatus; EDS45873.1	ereavqmdleryrdkadklqtalsrlqkerdilgdeleklkekaegtqssalkyqrerda	508
Anopheles darlingi; ETN64951.1	ekeaaqldldryrdkadklqstvarlqkerdilcdelekmkeksestqsnalkyqrerda	699
	:: .:* :* *:* :* * .:*:****** : ****.:**.:* .:*: :* *:*:*	
Drosophila melanogaster; AFH07983.1	mqtdlevlkeryekshaiqqklqmerddavteveilkekldkalyasqklidekdtsnke	1377
Culex quinquefasciatus; EDS45873.1	iqtelevvkerwekahqihqklqmerddavteidilkekldkalyasqklidekdnsnke	568
Anopheles darlingi; ETN64951.1	iqtelevvkerwekahqvhqklqmerddavteidilkekldkalyasqkvideketstke	759
	:**:***:***:**:* .:*************.:***************:****. *.**	
Drosophila melanogaster; AFH07983.1	fekmlekydraqneiyrlqsrcdtaeadrarleveaersglaaskaredlrklqdestrl	1437
Culex quinquefasciatus; EDS45873.1	fekmlekydrsqneiyrlqsrcdtaeadrnrleveaersalaaakakedlrklqeettrl	628
Anopheles darlingi; ETN64951.1	fekmlekydraqneiyrlqsrvdtaeadrnrleienersalatnkakedlrklqeetsrl	819
	**********:********** ******* ***:* ***.**: **:*******:*.:**	
Drosophila melanogaster; AFH07983.1	qeacdraalqlsrakecednarselehsrdrfdklqtdirraqgekehfqselervtyel	1497
Culex quinquefasciatus; EDS45873.1	qeacdraamqlgrskeledkakedvdlirerldktqadlrraqaekeniqaeverltyel	688
Anopheles darlingi; ETN64951.1	qeacdraamqlgrskeledkakedvdmirerldkcqtdlrraqsekenmqtelerltyel	879
	********:** *:** **:*: .::: *:*:** *:*:**** ***.:*:*:**:****	

Drosophila melanogaster; AFH07983.1	erahaaqtkasasveaakeeaahyavelekmrdryeksqvelrklqdtdtfgretrrlke	1557
Culex quinquefasciatus; EDS45873.1	drahnsytkqtasldaaqeeaarysleiekmreryektaaemrrlqenesfsresrrlke	748
Anopheles darlingi; ETN64951.1	drshnmytkqtasldsaqeevarygleiekmreryektaaelrrlqenesfsrearrmkd	939
	:*:* ** :**:::*:** *:* .*:****:****: .*:*:**: .::* **:**:*:	
Drosophila melanogaster; AFH07983.1	enerlrekldktlmeletirgksqyesesfekykdkyekiemevqnmesklhetslqlel	1617
Culex quinquefasciatus; EDS45873.1	endrlrekydkmmmelenfrsksqyeeetlekfkqkfelrenecqtmelklheatlqldl	808
Anopheles darlingi; ETN64951.1	endrlrekydkvivelenfrgksqyeqetfdkmkekfemrenefqtmelklhetslqldl	999
	**:***** ** ::*** .:* ***** *:::* *:*:* * * *.** ****::***:*	
Drosophila melanogaster; AFH07983.1	skgevakmlanqekqrselerahierekardkhekllkevdrlrlqqs-svspgdpvras	1676
Culex quinquefasciatus; EDS45873.1	arqevqkliasqdkqrtdaerahieyekirdkhekllkemerlrqnpgsavspgalaa--	866
Anopheles darlingi; ETN64951.1	araevakmvanqekqrtdserahieyekirdkhekllkeverlrqnpqsaispgaltt--	1057
	:: ** *::* *:***:: ****** ** **********::*** : ::*** .	
Drosophila melanogaster; AFH07983.1	tssssalsagerqeidrlrdrlekalqsrdateleagrlakelekaqmhlakqqentest	1736
Culex quinquefasciatus; EDS45873.1	----asvgmgdkneidrlrdrlekalqsrdatemeagrlakelekaqlhlakqqeayeat	922
Anopheles darlingi; ETN64951.1	--aatstgltdkneidrlrerlekalqsrdateleagrlakelekaqihltkqqevyeat	1115
	:: . :::******:*************:*************:**:**** *:*	
Drosophila melanogaster; AFH07983.1	riefermgaelgrlhdrlekaeaerealrqanrsggag---aaphpqlekhvqklesdvk	1793
Culex quinquefasciatus; EDS45873.1	riefermsaeltrvlerlekseaeketlrqstkiyekhhqqhaacnnidqnvlkieadnk	982
Anopheles darlingi; ETN64951.1	riefermsaeltrvlerlekseaeketlrqstkiyekhhqqhannhhvehnihkieadnk	1175
	*******.*** *: .****:***:*:***:.: * :::::: *:*:* *	

Drosophila melanogaster; AFH07983.1	qlamereqlvlqleksqeilmnfqkelqnaeaelqktreenrklrnghqvppvaappagp	1853
Culex quinquefasciatus; EDS45873.1	qlmaerdqlvmqleksqdmlmsfqqelnaaelelqrqceenrrlknspqgahg-------	1035
Anopheles darlingi; ETN64951.1	qlmaerdqlvmqleksqdmlmsfqqelnaaelelqrqceenrrlktspqppqstgpta--	1233
	** **:***:******::**.**:**: ** ***: ****:*:... *	

Drosophila melanogaster; AFH07983.1	spaefqamqkeiqtlqqklqeseralqaagpqqaqaaaaagasreeieqwrkvieqeksr	1913
Culex quinquefasciatus; EDS45873.1	---qpqnnsqeiqnykkeitrlkqmlqet----------gargdseleqwrkvveqeknr	1082
Anopheles darlingi; ETN64951.1	--aqnqataqeiqsykkeinklkqmlqet----------gcrgdneleqwrkvveqeknr	1281
	: * :***. :::: . :: ** : . . .*:******:****.*	

Drosophila melanogaster; AFH07983.1	admadkaaqemhkriqlmdqhikdqhaqmqkmqqqmqqqqqaaqqavqqaaqqqqsaaga	1973
Culex quinquefasciatus; EDS45873.1	adqaekaaielqkrmqvmeqqlkqqllqmtnmqkqmtqqqppqq----------ppqpqq	1132
Anopheles darlingi; ETN64951.1	adqaekaaielqkrmqlmeqqlkqqllqmtnmqkqmqqqstqqq----------------	1325
	** *:*** *::**:*:*:*::*:* ** :**:** **. *	

Drosophila melanogaster; AFH07983.1	ggadpkelekvrgelqaacterdrfqqqlellvtelekskmsnqeqakqlqtaqqqvqql	2033
Culex quinquefasciatus; EDS45873.1	pqinpkemdkiqeelknavtqrdqfqnqlellvqeleksqleaqdaakkaqqqhqklqqm	1192
Anopheles darlingi; ETN64951.1	spvnkkeleklqdelktavtqrdqfqnqlellvqeleksqteaqeatnkaqqqqqqlahl	1385
	: **::*:: **: * *:**:**:****** *****: . *: ::: * :*:: ::	

Drosophila melanogaster; AFH07983.1	qqqvqqlqqqmqqlqqaasa----gagatdvqrqqleqqqkqleevrkqidnqakatege	2089
Culex quinquefasciatus; EDS45873.1	eqqiqvlqqelqhatqaanqkaaqqagisehdr-------kqleaqmkqiedatrllese	1245
Anopheles darlingi; ETN64951.1	qqqiqqlqqqlqqvtqaanqkvaqnvgvseaer-------kqleaqmkqiedatrllese	1438
	:**:* ***::*: ***. .* :: :* **** ***:: :: *.*	

Drosophila melanogaster; AFH07983.1 rkiideqrkqidakrkdieekekkmaefdvqlrkrkeqmdqlekslqtqgggaaaageln 2149
Culex quinquefasciatus; EDS45873.1 rktfedqrkvienkrkeleekeknlvefdkqlkkrkeqmdqlekslqkaggtaaaageln 1305
Anopheles darlingi; ETN64951.1 rktfedqrktietkrkdledkeknliefdkqlkkrkeqmdqlekslqkaggttaaageln 1498
** :::*** *: ***::*:***:: *** **:**************. ** :*******

Drosophila melanogaster; AFH07983.1 kklmdtqrqleacvkelqntkeehkkaateterllqlvqmsqeeqnakektimdlqqalk 2209
Culex quinquefasciatus; EDS45873.1 kqltetqqqldkitkdfneakedaqrsaaeterllqlvqmtqeeqnqkektimdlqqalk 1365
Anopheles darlingi; ETN64951.1 kqltetqqmleqaskdlaeareeaqrsaaeterllqlvqmtqeeqnqkektimdlqqalk 1558
: :**: *: *:: :::*: :::*:***********:***** *************

Drosophila melanogaster; AFH07983.1 iaqakvkqaqtqqqqqqdagpagflksff 2238
Culex quinquefasciatus; EDS45873.1 naqaklktaqa---qpqdagpagflksff 1391
Anopheles darlingi; ETN64951.1 naqaklksaqa---qpqdagpagflksff 1584
****:* **: * *************

**Percent Identity Matrix for bruchpilot**	*Drosophila melanogaster*; AFH07983.1	*Culex quinquefasciatus*; EDS45873.1	*Anopheles darlingi*; ETN64951.1
Drosophila melanogaster; AFH07983.1	100.00	64.69	60.58
Culex quinquefasciatus; EDS45873.1	64.69	100.00	83.58
Anopheles darlingi; ETN64951.1	60.58	83.58	100.00

S1.2. Multiple sequence alignment for elav (Gene ID: 31000)

Drosophila melanogaster; AAA28506.1 mdfimantgagggvdtqaqlmqsaaaaaavaatnaaaapvqnaaavaaaaqlqqqqvqqa 60

Anopheles merus; XP_041763802.1 -- 0

Anopheles coluzzii; XP_040220033.1 -- 0

Culex quinquefasciatus; XP_038115089.1 -- 0

Culex pipiens pallens; XP_039431435.1 -- 0

Aedes aegypti; XP_001658986.1 -- 0

Drosophila melanogaster; AAA28506.1 ilqvqqqqtqqavaaaaaavtqqlqqqqqavvaqqavvqqqqqqaaavvqqaavqqavvp 120

Anopheles merus; XP_041763802.1 ------------mtnkvlaavqdlqkqqqqqqqqq--qqqqqq-----------qq--qq 33

Anopheles coluzzii; XP_040220033.1 ------------mtnkvlaavqdlqkqqqqqqqqq--qq--qq-----------qq--qq 31

Culex quinquefasciatus; XP_038115089.1 ------------mtnkvlaavqdlqkqngesqqnt------------------------- 23

Culex pipiens pallens; XP_039431435.1 ------------mtnkvlaavqdlqkqngesqqnt------------------------- 23

Aedes aegypti; XP_001658986.1 ------------mtnkvlaavqdlqkqngesqn-t-------------------------22

:: . *..*:**:*:

Drosophila melanogaster; AAA28506.1 qpqqaqpntngnag-------------sgsqngsngstetrtnlivnylpqtmtedeirs 167

Anopheles merus; XP_041763802.1 qqqqqqnganggggggggeagqtvaggaaggggqssdnnsrtnlivnylpqtmteeeirs 93

Anopheles coluzzii; XP_040220033.1 qqqqqqnganggggggggeagqtvaggaaggggqssdnnsrtnlivnylpqtmteeeirs 91

Culex quinquefasciatus; XP_038115089.1 ------------------------------taassgsdnartnlivnylpqtmteeeirs 53

Culex pipiens pallens; XP_039431435.1 ------------------------------taassgsdnartnlivnylpqtmteeeirs 53

Aedes aegypti; XP_001658986.1 ------------------------------aaaasgsetartnlivnylpqtmteeeirs 52

. ... :**************:****

Drosophila melanogaster; AAA28506.1	lfssvgeiesvklirdksqvyidplnpqapskgqslgygfvnyvrpqdaeqavnvlnglr	227
Anopheles merus; XP_041763802.1	lfssvgevesvklvrdknviypg------qpkgqslgygfvnyhrpqdaeqavnvlnglr	147
Anopheles coluzzii; XP_040220033.1	lfssvgevesvklvrdknviypg------qpkgqslgygfvnyhrpqdaeqavnvlnglr	145
Culex quinquefasciatus; XP_038115089.1	lfssvgevesvklvrdknviypg------qpkgqslgygfvnfhrsqdaeqavnvlnglr	107
Culex pipiens pallens; XP_039431435.1	lfssvgevesvklvrdknviypg------qpkgqslgygfvnfhrsqdaeqavnvlnglr	107
Aedes aegypti; XP_001658986.1	lfssvgevesvklvrdknviypg------qpkgqslgygfvnfhrsqdaeqavnvlnglr	106
	*******:*****:***. .:* . ***********: * **************	

Drosophila melanogaster; AAA28506.1	lqnktikvsfarpssdaikganlyvsglpktmtqqeleaifapfgaiitsrilqnagndt	287
Anopheles merus; XP_041763802.1	lqnkvlkvsfarpssegikganlyisglpktitqeeletifrpygeiitsrvliqdgnd-	206
Anopheles coluzzii; XP_040220033.1	lqnkvlkvsfarpssegikganlyisglpktitqeeletifrpygeiitsrvliqdgnd-	204
Culex quinquefasciatus; XP_038115089.1	lqnkvlkvsfarpssegikganlyisglpktitqeeleiifrpygeiitsrvlvqdgnd-	166
Culex pipiens pallens; XP_039431435.1	lqnkvlkvsfarpssegikganlyisglpktitqeeleiifrpygeiitsrvlvqdgnd-	166
Aedes aegypti; XP_001658986.1	lqnkvlkvsfarpssegikganlyisglpktitqeeletifrpygeiitsrvlvqegnd-	165
	****.:*********:.*******:******:**:*** ** *:* *****:* : ***	

Drosophila melanogaster; AAA28506.1	qtkgvgfirfdkreeatraiialngttpssctdpivvkfsntpgs--tskiiqpqlpafl	345
Anopheles merus; XP_041763802.1	kpkgvgfirfdqrkeaeraiqalngttpkgltdpitvkfsntpgqnaaakvvqpalpafl	266
Anopheles coluzzii; XP_040220033.1	kpkgvgfirfdqrkeaeraiqalngttpkgltdpitvkfsntpgqnaaakvvqpalpafl	264
Culex quinquefasciatus; XP_038115089.1	kpkgvgfirfdqrkeaeraiaalngttpkgltdpitvkfsntpgqnstakivqpalptfl	226
Culex pipiens pallens; XP_039431435.1	kpkgvgfirfdqrkeaeraiaalngttpkgltdpitvkfsntpgqnstakivqpalptfl	226
Aedes aegypti; XP_001658986.1	kpkgvgfirfdqrkeaeraiaalngttpkgltdpitvkfsntpgqntaakivqpalptfl	225
	: *********:*:** *** *******.. ****.********. ::*::** **:**	

Drosophila melanogaster; AAA28506.1	npqlvrriggamhtpvnkglarfspmagdmldvmlpnglgaaaaaattlasgpggaypif	405
Anopheles merus; XP_041763802.1	npqltrrlg-aihhpinkglarfspmggevldmmlpaapang-----ln-vapsggwsif	319
Anopheles coluzzii; XP_040220033.1	npqltrrlg-aihhpinkglarfspmggevldmmlpaapang-----ln-vapsggwsif	317
Culex quinquefasciatus; XP_038115089.1	npqltrrlg-aihhpinkglarfspmggevldmmlptaptng-----lgavapsggwsif	280
Culex pipiens pallens; XP_039431435.1	npqltrrlg-aihhpinkglarfspmggevldmmlptaptng-----lgavapsggwsif	280
Aedes aegypti; XP_001658986.1	npqltrrlg-aihhpinkglarfspmggevldmmlptapttg-----igaiapsggwsif	279
	****.**:* *:* *:***********.*::**:*** . . .*.*:: **	

Drosophila melanogaster; AAA28506.1	iynlapeteeaalwqlfgpfgavqsvkivkdpttnqckgygfvsmtnydeaamairalng	465
Anopheles merus; XP_041763802.1	iynlapeteentlwqlfgpfgavqnvkvikdaatnqckgygfvtmtnyeeamlairslng	379
Anopheles coluzzii; XP_040220033.1	iynlapeteentlwqlfgpfgavqnvkvikdaatnqckgygfvtmtnyeeamlairslng	377
Culex quinquefasciatus; XP_038115089.1	iynlapeteentlwqlfgpfgavqnvkiikdsatnqckgygfvtmtnyeeamlairslng	340
Culex pipiens pallens; XP_039431435.1	iynlapeteentlwqlfgpfgavqnvkiikdsatnqckgygfvtmtnyeeamlairslng	340
Aedes aegypti; XP_001658986.1	iynlapeteentlwqlfgpfgavqnvkvikdsatnqckgygfvtmtnyeeamlairslng	339
	********** :************.**::** :**********:****:** :***:***	

Drosophila melanogaster; AAA28506.1	ytmgnrvlqvsfktnkak------	483
Anopheles merus; XP_041763802.1	ytlgqrvlqvsfktnksnmqh---	400
Anopheles coluzzii; XP_040220033.1	ytlgqrvlqvsfktnksnmqh---	398
Culex quinquefasciatus; XP_038115089.1	ytlgqrvlqvsfktnksnsglgdh	364
Culex pipiens pallens; XP_039431435.1	ytlgqrvlqvsfktnksnsglgdh	364
Aedes aegypti; XP_001658986.1	ytlgqrvlqvsfktnkanaemadh	363
	**:*:************::	

**Percent Identity Matrix for elav**	*Drosophila melanogaster;* AAA28506.1	*Anopheles merus;* XP_041763802.1	*Anopheles coluzzii;* XP_040220033.1	*Culex quinquefasciatus;* XP_038115089.1	*Culex pipiens pallens;* XP_039431435.1	*Aedes aegypti;* XP_001658986.1
Drosophila melanogaster; AAA28506.1	100.00	69.37	69.21	70.22	70.22	69.86
Anopheles merus; XP_041763802.1	69.37	100.00	100.00	91.39	91.39	90.53
Anopheles coluzzii; XP_040220033.1	69.21	100.00	100.00	91.39	91.39	90.53
Culex quinquefasciatus; XP_038115089.1	70.22	91.39	91.39	100.00	100.00	95.04
Culex pipiens pallens; XP_039431435.1	70.22	91.39	91.39	100.00	100.00	95.04
Aedes aegypti; XP_001658986.1	69.86	90.53	90.53	95.04	95.04	100.00

S1.3. Multiple sequence alignment for futsch (Gene ID: 6724864)

Anopheles merus; XP_041782981.1	---miirssglfgldqhtma	17
Anopheles arabiensis; XP_040172246.1	---miirssglfgldqhtma	17
Anopheles coluzzii; XP_040218532.1	---miirssglfgldqhtma	17
Anopheles darlingi; ETN65512.1	----------------mgnvl-ckepnpgttasntgntfsnlssiicpnglskgkahpmr	43
Aedes albopictus; XP_029723621.1	mpittpkvpdglpelmrglaksvikenpeniyvhaaeyfen-lirerdggldrgyqnfsa	59
Drosophila melanogaster; ABW09325.1	----------------mgdqpkatttatggaagpvpegdav-mattnqdalakgagdgpa	43
Aedes aegypti; XP_021710788.1	---------------msgdqsgpielngtst-gingggagv-dgivtngg----------	33
Culex quinquefasciatus; EDS38616.1	--	0
Culex pipiens pallens; XP_039443854.1	--------------msagdqssplelnggsgaaatgggegl-ngnatengln--------	37

Anopheles merus; XP_041782981.1	yllvalalaliptahsapmyesdq------------------------------------	41
Anopheles arabiensis; XP_040172246.1	fllvalaltlipaahsapmyesdq------------------------------------	41
Anopheles coluzzii; XP_040218532.1	yllvalaltlipaahsapmyesdq------------------------------------	41
Anopheles darlingi; ETN65512.1	ktyftrs-----avd-askktssg-------httstgw-----------------qsvgl	73
Aedes albopictus; XP_029723621.1	ykvy--------adykekcrgkgggneslsggeip--------------ssaggvavrsr	97
Drosophila melanogaster; ABW09325.1	qdaaqep-----gqaehgepgdggd--ggddgatdagassl---p---pseiggrapldt	90
Aedes aegypti; XP_021710788.1	-----ip-----gge-stmnggggg--gge--aitpgggggsea-----gglgglstvda	73
Culex quinquefasciatus; EDS38616.1	--	0
Culex pipiens pallens; XP_039443854.1	----gap-----geg-atmnggggg--ggdggtvspgggagdvseeagtgagdpvmalda	85

Anopheles merus; XP_041782981.1	------------------telenygestgcyynynhygegdrimtnepclnctchdrmlm	83
Anopheles arabiensis; XP_040172246.1	------------------telenygestgcyynynhygegdrimtnepclnctchdrmlm	83
Anopheles coluzzii; XP_040218532.1	------------------telenygestgcyynynhygegdrimtnepclnctchdrmlm	83
Anopheles darlingi; ETN65512.1	hvpdg-----asnkvidtppvapprkkrgatldg----ryrsd-----------------	107
Aedes albopictus; XP_029723621.1	masdgdddsggsasatr-----------grrkkrv-rkqgske----------snk----	131
Drosophila melanogaster; ABW09325.1	acsdad---ggapsslaggivgppspltgcylliv-lgephse----------ehkdnil	136
Aedes aegypti; XP_021710788.1	gcseg-------pssii--gvgppspltgcyllii-igephsq----------ehkdiiv	113
Culex quinquefasciatus; EDS38616.1	--	0
Culex pipiens pallens; XP_039443854.1	gcsdg-------ppsii--gvgppspltgcyllii-vgephsq----------dhkdiil	125

Anopheles merus; XP_041782981.1	cylrvcpftkaigqdctiekredqccpvitcpevevqlvdhqttaspssalgatagsevg	143
Anopheles arabiensis; XP_040172246.1	cylrvcpftkaigqdctiekredqccpvitcpevevqlvdhqttaspssalgatagsevg	143
Anopheles coluzzii; XP_040218532.1	cylrvcpftkaigqdctiekredqccpvitcpevevqlvdhqttaspssalgatagsevg	143
Anopheles darlingi; ETN65512.1	--	107
Aedes albopictus; XP_029723621.1	----------------slekqesigsitenggeeek---------kpt---s-adgslqe	162
Drosophila melanogaster; ABW09325.1	-qhllkgflswdvsdchvdleeelntitqhape------------------g-eearhge	176
Aedes aegypti; XP_021710788.1	-qrlikgflswdatdchvdleeelhtitlqale------------------g-eegkhge	153
Culex quinquefasciatus; EDS38616.1	---mgkge	5
Culex pipiens pallens; XP_039443854.1	-qrlikgflswdvsdchvdleeelhtitlqale------------------g-eegkhge	165

Anopheles merus; XP_041782981.1	sldqygcsingrfypegaqvpsnpqkpcelcycirnmttcvmqectlhidgcqp------	197
Anopheles arabiensis; XP_040172246.1	sldqygcsingrfypegaqvpsnpqkpcelcycirnmttcvmqectlhidgcqp------	197
Anopheles coluzzii; XP_040218532.1	sldqygcsingrfypegaqvpsnpqkpcelcycirnmttcvmqectlhidgcqp------	197
Anopheles darlingi; ETN65512.1	--	107
Aedes albopictus; XP_029723621.1	slipikedtp-----dieeavv---kvqahlecqaprerpfnksvsv--dsvaaasavss	212
Drosophila melanogaster; ABW09325.1	rliqyasenl------vtevlihp-qyntliqcmrnllssftrhrhiihagytfsgngsw	229
Aedes aegypti; XP_021710788.1	rliqyasenl------vteilihp-qintfiqcirnllssftrhrhiihtgytfagngsw	206
Culex quinquefasciatus; EDS38616.1	rliqyasenl------vteilihp-qintfiqcirnllssftrhrhiihtgytfagngsw	58
Culex pipiens pallens; XP_039443854.1	rliqyasenl------vteilihp-qintfiqcirnllssftrhrhiihtgytfagngsw	218

Anopheles merus; XP_041782981.1	iynkg-------------------------------vccpvk-ydcdhdkd---------	216
Anopheles arabiensis; XP_040172246.1	iynkg-------------------------------vccpvk-ydcdhdkd---------	216
Anopheles coluzzii; XP_040218532.1	iynkg-------------------------------vccpvk-ydcdhdkd---------	216
Anopheles darlingi; ETN65512.1	--	107
Aedes albopictus; XP_029723621.1	vlqdageeaddadretgeaietqveadlaaelghpincedvdsyppdeted---------	263
Drosophila melanogaster; ABW09325.1	ilqdgtfsvadfseafqehdvqrvirayadtitmnihcadaglwhtlpekafarqcriri	289
Aedes aegypti; XP_021710788.1	ilqdgtfsvvdfmeafrehevqrvlraypdtitmdvhcapvgnwqaiqdksfarlcrirl	266
Culex quinquefasciatus; EDS38616.1	vlqdgtfsvvdfleafqehevqrvlraypdtitmdvhcapvgnwvsiqdktlarlcrvrl	118
Culex pipiens pallens; XP_039443854.1	vlqdgtfsvvdfleafqehevqrvlraypdtitmdvhcapvgnwvsiqdktlarlcrvrl	278

Anopheles merus; XP_041782981.1	-stlm----------ledehtttvrpt--------------pgfilt-ttvspavstdcv	250
Anopheles arabiensis; XP_040172246.1	-stlm----------ledehtttvrpt--------------pgfilt-ttvspavstdcv	250
Anopheles coluzzii; XP_040218532.1	-stlm----------ledehtttvrpt--------------pgfilt-ttvspavstdcv	250
Anopheles darlingi; ETN65512.1	--	107
Aedes albopictus; XP_029723621.1	--------dpeagng-edygsapstae------vggevigdaevlvn--dappngsdqvv	306
Drosophila melanogaster; ABW09325.1	npvdvldtssecingfidylapmvmptslrelletsdvvgnirfthptlyvfpggqgdaa	349
Aedes aegypti; XP_021710788.1	npvdvlssgseklnafvdylasmivpteisellessdvvgnirfshptlyvfpggqgdaa	326
Culex quinquefasciatus; EDS38616.1	npvdvlssgsdklnafvdylasmivpteisellessdvvgnirfshptlyvfpggqgdaa	178
Culex pipiens pallens; XP_039443854.1	npvdvlssgsdklnafvdylasmivpteisellessdvvgnirfshptlyvfpggqgdaa	338

Anopheles merus; XP_041782981.1	hnge--tyadgalimtd--kpcehcycmrgdivcavqecgtplenegknctalppaagqc	306
Anopheles arabiensis; XP_040172246.1	hnge--tyadgalimtd--kpcehcycmrgdivcavqecgtplenegknctalppaagqc	306
Anopheles coluzzii; XP_040218532.1	hnge--tyadgalimtd--kpcehcycmrgdivcavqecgtplenegknctalppaagqc	306
Anopheles darlingi; ETN65512.1	---ealkvkngfqdvfgresrrhscdithgtrpasv------------------------	140
Aedes albopictus; XP_029723621.1	viessqknsdevdlsvddvannneeplangsveeqi------------------------	342
Drosophila melanogaster; ABW09325.1	lfgi-----ngfnmlvdggfnrkacfw---dfarhl------------------------	377
Aedes aegypti; XP_021710788.1	lfgi-----ngfnmlvdggfsrkscfw---dfvrhl------------------------	354
Culex quinquefasciatus; EDS38616.1	lfgi-----ngfnmlvdggfsrkscfw---dfvrhl------------------------	206
Culex pipiens pallens; XP_039443854.1	lfgi-----ngfnmlvdggfsrkscfw---dfvrhl------------------------	366
	: . . :	

Anopheles merus; XP_041782981.1	cpdkyicdgsaaapmtttvpavtaaadeaeeqeqeqeqvaqsfdkaqpeqdeqqseatlt	366
Anopheles arabiensis; XP_040172246.1	cpdkyicdgsaaapmtttvpavtaaaddaeeqeeeqeqvaqsfdkaqpeqdeqqseatlt	366
Anopheles coluzzii; XP_040218532.1	cpdkyicdgsaaapmtttvpavttaaddveeqeqeqeqvaqsfdkaqpeqdeqqseatlt	366
Anopheles darlingi; ETN65512.1	-pprrledslpamparqtscfeidasnd----------afsdfs----------------	173
Aedes albopictus; XP_029723621.1	-------dgaaeasve--	351
Drosophila melanogaster; ABW09325.1	-------drldavlmt--	386
Aedes aegypti; XP_021710788.1	-------drldavlmt--	363
Culex quinquefasciatus; EDS38616.1	-------drldavlmt--	215
Culex pipiens pallens; XP_039443854.1	-------drldavlmt--	375
	*	

Anopheles merus; XP_041782981.1	ttaasverttmpqhddvvpvekeeidseddqqeqehvvpqfedvqtddshe---------	417
Anopheles arabiensis; XP_040172246.1	ttaasverttmpqhddvvpvekeeidseddqqeqehvvpqfedvqtddshe---------	417
Anopheles coluzzii; XP_040218532.1	ttaasverttmpqhddvvpvekeeidseddqqeqehvvpqfedvqtddshe---------	417
Anopheles darlingi; ETN65512.1	---------kvpttaelerspaaqiprvgnrks--------drffgehlsdslspeqdak	216
Aedes albopictus; XP_029723621.1	-------------kqdsaevqiegvdtvde---svpvveevdesvpnlp-----------	384
Drosophila melanogaster; ABW09325.1	---------------rlnnsnvqglgavvsrkrdahvypqighffgnvp-----------	420
Aedes aegypti; XP_021710788.1	---------------rinntnikgisavverkheahvypqighffcnip-----------	397
Culex quinquefasciatus; EDS38616.1	---------------rinntnikgisavverkheahvypqighfftnip-----------	249
Culex pipiens pallens; XP_039443854.1	---------------rinntnikgisavverkheahvypqighfftnip-----------	409
	: . :	

Anopheles merus; XP_041782981.1	-------------eaddddaqvpek---ved---------eqvatt--------------	438
Anopheles arabiensis; XP_040172246.1	-------------eaddddaqvpek---vdd---------eqvatt--------------	438
Anopheles coluzzii; XP_040218532.1	-------------eaddddaqvpek---vdd---------eqvatt--------------	438
Anopheles darlingi; ETN65512.1	espeqrpkrreksipkddvdkiekfvekleenkpvevsma--------------------	256
Aedes albopictus; XP_029723621.1	dvpteepnieevesgdteevqaepivdepneqkpvddetkedivedanviksskessvdk	444
Drosophila melanogaster; ABW09325.1	dr-------kglpspdgdkdrdpllidlfer--------ghgivsdlkaldlkpqccyrn	465
Aedes aegypti; XP_021710788.1	er-------kgllspdgdkdrdpllvdliee--------ghqivtnlkslnlkaqncyrd	442
Culex quinquefasciatus; EDS38616.1	er-------kgllspdgdkdrdpllvdliee--------ghqivtnlkslnlkaqncyrd	294
Culex pipiens pallens; XP_039443854.1	er-------kgllspdgdkdrdpllvdliee--------ghqivtnlkslnlkaqncyrd	454
	. : : :	

Anopheles merus; XP_041782981.1	--------------arpslseapeaesdhipghvgsheseedkqqeattvadldaplttm	484
Anopheles arabiensis; XP_040172246.1	--------------arpslseapeaesdhipghvgsheseedkqqeattvadldaplttm	484
Anopheles coluzzii; XP_040218532.1	--------------arpslseapeaesdhipghvgsheseedkqqeattvadlnaplttm	484
Anopheles darlingi; ETN65512.1	--	256
Aedes albopictus; XP_029723621.1	vdeasaaddatkdgkvksdaiedgaegqevpsn----kadsieevspkevneng------	494
Drosophila melanogaster; ABW09325.1	qepvn-lyhkvghgtldmyvispardskevkeflqkwhagdqrlfaardsrdfnfplqnl	524
Aedes aegypti; XP_021710788.1	vepin-lyhkvghgtldmyiispakdskevkeflakwnaadpklfaprdskefvfpmqni	501
Culex quinquefasciatus; EDS38616.1	adpin-lyhkvghgtldmyvispakdskevkeflakwhaadpklfspkdskeftfpmqni	353
Culex pipiens pallens; XP_039443854.1	adpin-lyhkvghgtldmyvispakdskevkeflakwhaadpklfspkdskeftfpmqni	513

Anopheles merus; XP_041782981.1	r---------pavqstt-------------------------vlaeqegttshadvdve	509
Anopheles arabiensis; XP_040172246.1	r---------pavqstt-------------------------vlveqesttshadvdae	509
Anopheles coluzzii; XP_040218532.1	r---------pavqstt-------------------------vlaeqesttshadvdae	509
Anopheles darlingi; ETN65512.1	---	256
Aedes albopictus; XP_029723621.1	-saspplvkseskdnetvgvasspkqirsveddtngnpsgegpvdkaveevaees-----	548
Drosophila melanogaster; ABW09325.1	vsicallvwqpanpddtitri---------------lfpgstpdfkiqeglekIkhlefm	569
Aedes aegypti; XP_021710788.1	isicallvwtpanpednitri---------------lfpgsapeykileglekmknvefm	546
Culex quinquefasciatus; EDS38616.1	isicallvwtpanpednitri---------------lfpgsapeykileglekmksvefm	398
Culex pipiens pallens; XP_039443854.1	isicallvwtpanpednitri---------------lfpgsapeykileglekmksvefm	558

Anopheles merus; XP_041782981.1	ehdvt--------------------gttvaptkdeqapeadavpfeeeedeapvttkpas	549
Anopheles arabiensis; XP_040172246.1	ehdvt--------------------gttvaptideqapeadaapfeeeedeapvttkpas	549
Anopheles coluzzii; XP_040218532.1	ehdvt--------------------gttvaptkdeqapeadavsfeeeedeapvttkpaf	549
Anopheles darlingi; ETN65512.1	-----qkeaiqkssntftsrqkdiekdevpaalpktappdeapasgeneedli-------	304
Aedes albopictus; XP_029723621.1	-spva----------------------vegpedeqadpseaqpenvngnesg-------	577
Drosophila melanogaster; ABW09325.1	khstctaksiapaiqtvtstrkslksaieatp----appsasykt---------------	610
Aedes aegypti; XP_021710788.1	rqpvctvksiapslstqaltkkslksssidkiipepilpskstkp---------------	591
Culex quinquefasciatus; EDS38616.1	rhpvctvksiapsistqtftkkslksssidkiipepilpskatks---------------	443
Culex pipiens pallens; XP_039443854.1	rhpvctvksiapsistqtftkkslksssidkiipepilpskatks---------------	603
	:	

Anopheles merus; XP_041782981.1	deiaapeaeat-tv-srdetve-------qtmt--talpsqqvplasadekeneiddhhd	598
Anopheles arabiensis; XP_040172246.1	deiaapeaeat-tv-srdetve-------qtmt--talpsqqvplasadekeneiddhhd	598
Anopheles coluzzii; XP_040218532.1	deiaapeaeat-tv-srdetve-------qtmt--talpsqqvpltsadekeneiddhhd	598
Anopheles darlingi; ETN65512.1	kyid-rtvsgnsqlgsraeflmamledyndsvryegmqpieeplivpkkrksrhicddhd	363
Aedes albopictus; XP_029723621.1	gsldnppenga--------------------------pvdnvpeevevsask-n-----	604
Drosophila melanogaster; ABW09325.1	tkf-spvasaa--------------------lavqhpqqqdnkakeaaaaaa--------	641
Aedes aegypti; XP_021710788.1	sekdnkildnk--------------------mk-----midnkltds-ksstd-m-----	619
Culex quinquefasciatus; EDS38616.1	lekdnrnidnk--------------------lk-----midnklvdsaksssd-i-----	472
Culex pipiens pallens; XP_039443854.1	lekdnrnidnk--------------------lk-----midnklvdsaksssd-i-----	632
	. ::	

Anopheles merus; XP_041782981.1	qtnaaehddgasadipeeqkeedeqveqeeqqeaattpvvpvrda------vpseqdqqt	652
Anopheles arabiensis; XP_040172246.1	qtnavehddgasadipeeqkeedvqveqeeqqeavttpvvpvrde------vpseqdqqt	652
Anopheles coluzzii; XP_040218532.1	qtnavehddgasadipeeqkeedeqfeqeeqqeaattpvvpvhde------vpseqdqqt	652
Anopheles darlingi; ETN65512.1	hlheklhahe----kgestkq-ekpa-aniilteasiev-aprkps---rdfs------k	407
Aedes albopictus; XP_029723621.1	---------------peatksngksg-eatplksesveqidaqsa---gedip----ses	641
Drosophila melanogaster; ABW09325.1	----------------------------aaaaaaasaatiaraka---------------	658
Aedes aegypti; XP_021710788.1	---------------mdssadeak---lekhitkssvatvatakvtvskskviktsstes	661
Culex quinquefasciatus; EDS38616.1	---------------mdssadeak---lekqltkssvatvatakvmsktvk-sksstest	513
Culex pipiens pallens; XP_039443854.1	---------------mdssadeak---lekqltkssvatvatakvmsktvk-sksstest	673
	: .	

Anopheles merus; XP_041782981.1	yqpseqevsapeqdrpavvstdkkepaqkaddepqvp-veqdfvelppavvvtelppvpt	711
Anopheles arabiensis; XP_040172246.1	yqpsepevsvpeqdrpavvstdkkepaqkaddepqmp-veqdfvelppavvvtelppvpt	711
Anopheles coluzzii; XP_040218532.1	yqpsepevsvpeqdrpavvsadkkepaqkaddepqvp-veqdfvelppavvvtelppvpt	711
Anopheles darlingi; ETN65512.1	ykpldke-----------vg--kidqefdsdeepsssgaataavv-rpsr---------t	444
Aedes albopictus; XP_029723621.1	aenvesn------dap--gseataqpedgvqensngsgeekgkaskepsl----------	683
Drosophila melanogaster; ABW09325.1	----dsm-----------dtda--epeheadpepadtgdeaapteqepea----------	691
Aedes aegypti; XP_021710788.1	tkkidsm-----------liesskkidldekkdssdagsdkdetekkvaa----------	700
Culex quinquefasciatus; EDS38616.1	tkkvdnk-----------lietkkieseekkdsasdagsekdetekkast----------	552
Culex pipiens pallens; XP_039443854.1	tkkvdnk-----------lietkkieseekkdsasdagsekdetekkast----------	712
	: . . .	

Anopheles merus; XP_041782981.1	tsdepstteqtpeppkdeilpnm------pleadshvlsds-----------------mt	748
Anopheles arabiensis; XP_040172246.1	tsdepstteqtpeppkdeilpnm------pleadshilsds-----------------mt	748
Anopheles coluzzii; XP_040218532.1	tsdepstteqtpeppkdeilpnm------pleadshvlsds-----------------mt	748
Anopheles darlingi; ETN65512.1	kqakskqrenlpsppapp---kfqkslse------slfd---------------------	474
Aedes albopictus; XP_029723621.1	--------d-kpes-------ekgesadepkssg------------saaasrepsvdkve	715
Drosophila melanogaster; ABW09325.1	--------etepepehep-eaeqdkdvgeekkvevlimkpqqatpaviaasgkdgvdaas	742
Aedes aegypti; XP_021710788.1	--------e-eaipekklvqdkkdddakdskkddsitikdetk-----------------	734
Culex quinquefasciatus; EDS38616.1	--------e-eae-----------kkpeedkkedtaiikeetk-----------------	575
Culex pipiens pallens; XP_039443854.1	--------e-eae-----------kkpeedkkedtaiikeetk-----------------	735
	:	

Anopheles merus; XP_041782981.1	tehaptfgdr-------keddgqeqeqhpvqggadldep----vemntiipleaeedqkv	797
Anopheles arabiensis; XP_040172246.1	tehaptfgdr-------keddaqeqeqhpvqggadldep----vemntiipldaeedqkv	797
Anopheles coluzzii; XP_040218532.1	tehaptfgdr-------keddaqeqeqhpvqggadldep----vemntiipldaeedqkv	797
Anopheles darlingi; ETN65512.1	------------------rqvsreststpvksvvddtpkahtprmlkriismpvteh---	513
Aedes albopictus; XP_029723621.1	s------------ekegsadepkssgsakas----nep----------sldkpesek---	746
Drosophila melanogaster; ABW09325.1	adatp-tgklskasakgkadkpraevkpvvrsridtkpp----ksmdrklakrdekk---	794
Aedes aegypti; XP_021710788.1	---------------vakritekssrpstarsridtkpp----kmaakpmkkkeean---	772
Culex quinquefasciatus; EDS38616.1	---------------stkkigdrvsrpatarsrvdtkpp----kpttktikkkdett---	613
Culex pipiens pallens; XP_039443854.1	---------------ttkkigdrvgrpatarsrvdtkpp----kpttktikkkdett---	773
	: . :	

Anopheles merus; XP_041782981.1	ehskeedqkvdeqdeqeqqvqehaqdvvpdthadddeqaqsgeepevvtpvsvdaqpepa	857
Anopheles arabiensis; XP_040172246.1	ehpkeedqkvdeqdeqdeqvqeqaqdvvpdthadaddqaqsgeepdvvtpvsddaqpepa	857
Anopheles coluzzii; XP_040218532.1	ehpkeedqkideqdeqdeqvqeqaqdvvpdthaddddqpqsgeeqevvtpvsddaqpepa	857
Anopheles darlingi; ETN65512.1	--------------------------------lnadarpetpqrpaltkssssssfst--	539
Aedes albopictus; XP_029723621.1	--------------------------------ngsqeepks-dkpesekdvsadepksn-	772
Drosophila melanogaster; ABW09325.1	--------------------------------ssptttpaar-apvaqna----------	811
Aedes aegypti; XP_021710788.1	--------------------------------kssptttpkksvepkltngiskdeeskk-	799
Culex quinquefasciatus; EDS38616.1	--------------------------------ksspttpkktaepkltngvskdddakk-	640
Culex pipiens pallens; XP_039443854.1	--------------------------------ksspttpkktaepkltngvskdedakk-	800
	. .	

Anopheles merus; XP_041782981.1	apvdeklgeddeqqsvhespdqlfpesipg-------egdclid--------gvtyen--	900
Anopheles arabiensis; XP_040172246.1	apve-kvgeddeqqtvhespdqlfpesipg-------egdclid--------gvtyen--	899
Anopheles coluzzii; XP_040218532.1	apve-kvgeddeqqtvhespdqlfpesipg-------egdclid--------gvtyen--	899
Anopheles darlingi; ETN65512.1	--------------vdlqrsricverfspedyahhgggadelvqpksklitrkiswksle	585
Aedes albopictus; XP_029723621.1	-------gsa----qaskepsldkpksesvdepksggsak--------------------	801
Drosophila melanogaster; ABW09325.1	-----------kpkvlsrpatksspsstpaksakeannrkvl-eskq-------------	846
Aedes aegypti; XP_021710788.1	-----ttappkttkpvttrpgkaspkstpaksakdannrkvl-earq-------------	840
Culex quinquefasciatus; EDS38616.1	-----atgppkttkpastkpgkaspkstpaksakdannrkvl-earq-------------	681
Culex pipiens pallens; XP_039443854.1	-----atgppkttkpastkpgkaspkstpaksakdannrkvl-earq-------------	841
	.	

Anopheles merus; XP_041782981.1	-------gasvpa---------sgkcqvachcsnsiv--hcemvrceaapsadctpkstl	942
Anopheles arabiensis; XP_040172246.1	-------gasvpa---------sgkcqvachcsnsiv--hcemvrceaapsadctpkstl	941
Anopheles coluzzii; XP_040218532.1	-------gasvpa---------sgkcqvachcsnsiv--hcemvrcdaapsadctpkstl	941
Anopheles darlingi; ETN65512.1	tpatnrtptptqdtat-npcftigdaavvassgntlqppevpk-rkrsttstemetiger	643
Aedes albopictus; XP_029723621.1	--------------sskepsldk-----peseknm----sadevksggsaksskdpsldk	838
Drosophila melanogaster; ABW09325.1	q------aarvqatstvsrrvts-----taserrvqqqaeaktaatgatqatqrkpisrr	895
Aedes aegypti; XP_021710788.1	r------paakpapakkd-----------------------smekkdsppkverkpisrr	871
Culex quinquefasciatus; EDS38616.1	r------papkaa-akke-----------------------paekkeappkverkpisrr	711
Culex pipiens pallens; XP_039443854.1	r------papkaa-akke-----------------------paekkeappkverkpisrr	871
	. .	

Anopheles merus; XP_041782981.1	pge-------ccpsys------------c---pkeasttvs---sasvesteastesdae	977
Anopheles arabiensis; XP_040172246.1	pge-------ccpsys------------c---pkeasttvs---sasvesteastesdae	976
Anopheles coluzzii; XP_040218532.1	pge-------ccpsys------------c---pkeasttvs---sasvesteastesdae	976
Anopheles darlingi; ETN65512.1	pdastpaepscipaaqheepadsalrkfclgsfiessnvlqhhdvvsv----ldrvysas	699
Aedes albopictus; XP_029723621.1	pksennes---------------aiepkssgsa-ka------skepsqdnpdsdkngnmd	876
Drosophila melanogaster; ABW09325.1	prgvspsk---------------ra-p-apgspvkqak----pkaadlkktrldkggttd	934
Aedes aegypti; XP_021710788.1	prgpts-----------------at-a-vggspvkakkvlksekdavirkakldkngttd	912
Culex quinquefasciatus; EDS38616.1	pkgpas-----------------aa-a-vggspvkakkvlksekdavirkakldkngttd	752
Culex pipiens pallens; XP_039443854.1	pkgpas-----------------aa-a-vggspvkakkvlksekdavirkakldkngttd	912
	* : . .	

Anopheles merus; XP_041782981.1	se------------------eeqvsadvs---asdsgas----dvesaeattkapa-sds	1011
Anopheles arabiensis; XP_040172246.1	se------------------eeqvsadvs---asdsgas----nvesaeattkapa-sds	1010
Anopheles coluzzii; XP_040218532.1	se------------------eeqvsadvs---asdsgas----nvesaeattkapa-sds	1010
Anopheles darlingi; ETN65512.1	dnken--iieqfqtflen----qinaelndpnptnpnvakllekl---srtstasgg---	747
Aedes albopictus; XP_029723621.1	epkangsakasqetsldkpnsekddgsveepkssgsakaskepsleqaepdlksdgsakv	936
Drosophila melanogaster; ABW09325.1	ss------------------------lvstpsadeataakklq---------------dl	955
Aedes aegypti; XP_021710788.1	ss------------------------lvstpsadeagiavqkriid----ttas----aa	940
Culex quinquefasciatus; EDS38616.1	ss------------------------lvstpsadeagvavqkrivd----svtlagtssa	784
Culex pipiens pallens; XP_039443854.1	ss------------------------lvstpsadeagvavqkrivd----svtlagtssa	944
	. :. :	

Anopheles merus; XP_041782981.1	qslstdeeddddvsdkp---seaesdsagvdlddevfkpyrptepshget---------h	1059
Anopheles arabiensis; XP_040172246.1	qslstdeeddddvsdkp---seaesdsagadlddevfkpyrptepshget---------h	1058
Anopheles coluzzii; XP_040218532.1	qslstdeeddddvsdkp---seaesdsagadlddevfkpyrptepshget---------h	1058
Anopheles darlingi; ETN65512.1	-------sdledvkiel--vgsssassnnasdvddcfdpefekiekfeivgdlpkidipp	798
Aedes albopictus; XP_029723621.1	psldkteseldqsngdqskessakesagesgsveavtapdeggsadagemsm--------	988
Drosophila melanogaster; ABW09325.1	t----asqeldaekqre--lddlkeeqevvreieavfsrdemkrqqhqqika--------	1001
Aedes aegypti; XP_021710788.1	a----gtsdmdaiqqqq--laelkeeqeavreieavfkrdsekikrheil-s--------	985
Culex quinquefasciatus; EDS38616.1	a----sstdidpiqqqq--laelkeeqeavreieavfnkg--kvtrrevl-s--------	827
Culex pipiens pallens; XP_039443854.1	a----sstdidpiqqqq--laelkeeqeavreieavfnkg--kvtrrevl-s--------	987
	: : . . . . :	

Anopheles merus; XP_041782981.1	inffagnrpttadyhdeiilstaaptkvpvvvsaqdddvaqedpvkmeq----peqdaap	1115
Anopheles arabiensis; XP_040172246.1	inffagnrpttadyhdeiilstvapskvpvvvstqdddvaqedpvkmeq----peqdaap	1114
Anopheles coluzzii; XP_040218532.1	inffagnrpttadyhdeiilstaaptkvpvvvstqdddvaqedpvkmeq----qeqdaap	1114
Anopheles darlingi; ETN65512.1	khaysgkrrdsieymsdwfgdsdgstteekasg----gvknkpavgakgkrresiedvgs	854
Aedes albopictus; XP_029723621.1	--	988
Drosophila melanogaster; ABW09325.1	------------------------------------elre--------------------	1005
Aedes aegypti; XP_021710788.1	-------------------------------------ehr--------------------	988
Culex quinquefasciatus; EDS38616.1	-------------------------------------ehr--------------------	830
Culex pipiens pallens; XP_039443854.1	-------------------------------------ehr--------------------	990

Anopheles merus; XP_041782981.1	attqasvte----salqeegttlrtaergtesdesvaaeqdeqeqatttktfteet---g	1168
Anopheles arabiensis; XP_040172246.1	attqasvte----salqeegttlrtaergpesdesvaaeqdeqeqvttaktfaeet---g	1167
Anopheles coluzzii; XP_040218532.1	attqasvte----salqeegttlrtaergpesdesvaaeqddqeevttaktfaeet---g	1167
Anopheles darlingi; ETN65512.1	wfsnhnmlrpsfgqefeepsrrlrrgs-----dgflgydm--nrqypfgkvrersesqsa	907
Aedes albopictus; XP_029723621.1	--------ssrmgddeqepes-----e-----a----------------vdepsa--pp	1011
Drosophila melanogaster; ABW09325.1	------mpaegtgdgenepde-----e-----eeyliiekeeveqytedsiveqes--sm	1047
Aedes aegypti; XP_021710788.1	----------eiqdsttepee-----e-----eeyliieke--eqytedsinepes--sa	1024
Culex quinquefasciatus; EDS38616.1	----------eiqdsttepee-----e-----deyliieke--eqytedsinepes--sa	866
Culex pipiens pallens; XP_039443854.1	----------eiqdsttepee-----e-----deyliieke--eqytedsinepes--sa	1026
	. * . .	

Anopheles merus; XP_041782981.1	ttlasvvkteedse---a------------edkdakped------------ttsgvetdq	1201
Anopheles arabiensis; XP_040172246.1	ttlasvvkpeedsv---veeg-------qrgdddakped------------ttsgvetdq	1205
Anopheles coluzzii; XP_040218532.1	ttlasvvkpeedsv---veeg-------qrgeddakped------------ttsgvetdq	1205
Anopheles darlingi; ETN65512.1	emfeditkhqetpgdkegth--sler----------------------------------	931
Aedes albopictus; XP_029723621.1	medeetget----ggedkvksdel----kteddsakndg---ngeettadqvnqgddeet	1060
Drosophila melanogaster; ABW09325.1	tkeeeiqkhqrdsqesekkrkksaeeeieaa--iakvea-------aerkarlegasarq	1098
Aedes aegypti; XP_021710788.1	tkeeeiqkhqrdsqesekckrdsmegekqekadvgkmeg-----etatvveiiegdkvde	1079
Culex quinquefasciatus; EDS38616.1	tkeeeiqkhqrdsqesekrkresledekeekadvgktddeaadgaaaavveliegekdea	926
Culex pipiens pallens; XP_039443854.1	tkeeeiqkhqrdsqesekrkresledekeekadvgktedeaadgaaaavveliegekdea	1086
	. :	

Anopheles merus; XP_041782981.1	qd--------krvttvtadeveddkeqat--------tastissd------kqqevskad	1239
Anopheles arabiensis; XP_040172246.1	qd--------krvttvvadevendkdqat--------tvstissd------kqqevskad	1243
Anopheles coluzzii; XP_040218532.1	qd--------krvttvvadeveddkeqat--------tvstissd------kqqevskad	1243
Anopheles darlingi; ETN65512.1	---------------------------kglsralpiherkvlehrnssdallmkvlnre	963
Aedes albopictus; XP_029723621.1	de--------skeevveepciq----------rqvspsaskipskqps-v-elddikkvd	1100
Drosophila melanogaster; ABW09325.1	de---------------------------seldvepeqskik------a-evqdiiata	1123
Aedes aegypti; XP_021710788.1	ehpaeethqedketilekeaiedvkeepkdteklsakhkqdie------e-evqdiiasa	1132
Culex quinquefasciatus; EDS38616.1	eeeep--kpedke----seeekvdaeeskepekmspkhkhele------e-evqeiiasa	973
Culex pipiens pallens; XP_039443854.1	eeeep--kpevke----seeekvdaeeskepekmspkhkhdle------e-evqeiiasa	1133
	: .:	

Anopheles merus; XP_041782981.1	-dgvttsssvted-----------tavsveqdekleqpapttartveadeetv-v-----	1281
Anopheles arabiensis; XP_040172246.1	-dgvttsssvaad-----------tpvsveqdekleqpapttvrtveadeeaa-v-----	1285
Anopheles coluzzii; XP_040218532.1	-devttsssvaed-----------tpvsveqdekleqpapttvrtveadedta-v-----	1285
Anopheles darlingi; ETN65512.1	-hrishssahssaeklnne---------skpqemask------stsdptdeee----qkp	1003
Aedes albopictus; XP_029723621.1	lasfnkdsaealfytlkkselenqesqatkpeakven----gveenedddddvvvteepp	1156
Drosophila melanogaster; ABW09325.1	-kdiaksrteeqlakpaee-----elssptpeeklsk------ktsdtkddqi----gap	1167
Aedes aegypti; XP_021710788.1	-keiakskmetsmdglkte-----emssispdekvss----tkktsdtrdene----pep	1178
Culex quinquefasciatus; EDS38616.1	-keiakskmetsmdglkte-----emssispddkiss----tkktsdtrdene----pep	1019
Culex pipiens pallens; XP_039443854.1	-keiakskmetsmdglkte-----emssispddkiss----tkktsdtrdene----pep	1179
	. . : .. : ::	

Anopheles merus; XP_041782981.1	--eqeqdqpqssddqaeeddepssttvha-------dkpsaveedeegpaqeattaraie	1332
Anopheles arabiensis; XP_040172246.1	--aqeqdqpqssddqaedddepssttvra-------dkpssveedeegpaqeatttrate	1336
Anopheles coluzzii; XP_040218532.1	--aqeqdqpqssddqaedddepssttvra-------dkpssveqdeegpaqeattarvte	1336
Anopheles darlingi; ETN65512.1	l--------lsstseasvva----aeisne------sakqssnkdsveplqaanehstll	1045
Aedes albopictus; XP_029723621.1	srvsrv-spkrsftdnflesspitdevsksaegdgvadqdgveedenqqfnpmfa-----	1210
Drosophila melanogaster; ABW09325.1	vdvlpv-nlqeslpe---------ekfsatiesga-ttaptlpederipldqikedlvie	1216
Aedes aegypti; XP_021710788.1	vqkehieppheshhe---------ervsataesgatttaptlpederipldeikedlvie	1229
Culex quinquefasciatus; EDS38616.1	vvkghieppheshhe---------ervsatgesgatttaptlpederipldeikedlvie	1070
Culex pipiens pallens; XP_039443854.1	vvkghieppheshhe---------ervsatgesgatttaptlpederipldeikedlvie	1230
	* : . :*. :	

Anopheles merus; XP_041782981.1	tateqqesvpeietkrktiqttvap-ttvseq-------qddlektetttqpaa-saaaa	1383
Anopheles arabiensis; XP_040172246.1	tateqeesvteietkrktiqttvap-ttvseq-------qddlektetttqpaa-saaaa	1387
Anopheles coluzzii; XP_040218532.1	tateqeesvtefetkrktiqttvap-ttvseq-------qddlektetttqpaa-saaaa	1387
Anopheles darlingi; ETN65512.1	--	1045
Aedes albopictus; XP_029723621.1	-------------------------asvrnkq----l-qdqlhsrfsqdd---tskldn	1236
Drosophila melanogaster; ABW09325.1	ekyv----------keetkeaeaivvatvqtlpeaaplaidtilasatkdapkdanaeal	1266
Aedes aegypti; XP_021710788.1	ekyv----------keetkevevappl---------------------------------	1246
Culex quinquefasciatus; EDS38616.1	ekhv----------keetkevevappp---------------------------------	1087
Culex pipiens pallens; XP_039443854.1	ekhv----------keetkevevappp---------------------------------	1247

Anopheles merus; XP_041782981.1	delqee----eheeekltdepelteddvqesaapttsrptaqeeastqydhepetthkss	1439
Anopheles arabiensis; XP_040172246.1	delqee----eheevkltdepeltednaqesaaattsrptvqeeastqydhepetthkps	1443
Anopheles coluzzii; XP_040218532.1	delqee----ehqevkltdepelteddaqesaaattsrptgqeeastqydhvpdsthkps	1443
Anopheles darlingi; ETN65512.1	---------------kflskerlie-----------------------------------	1055
Aedes albopictus; XP_029723621.1	strraamhrsmtermdlarqdtnyvd-lrkydpd----yveeedqfdgyyignlk-----	1286
Drosophila melanogaster; ABW09325.1	gelpdsgervlpmkmtfeaqqnllrd-viktpdevadlpvheeadlglyekdsqdagaks	1325
Aedes aegypti; XP_021710788.1	ptvyeplerptpakmhfsaqqahmrd-vvktpdevadlpmheeadfeeysenkdkedeke	1305
Culex quinquefasciatus; EDS38616.1	ptvyeplerptpakmhfnaqqphmrd-vvktpdevadlpvheeadfeeygedkdkddeke	1146
Culex pipiens pallens; XP_039443854.1	ptvyeplerptpakmhfnaqqphmrd-vvktpdevadlpvheeadfeeygedkdkddeke	1306
	: :	

Anopheles merus; XP_041782981.1	evhddeqpgttvssvtsekesdvpvavtvqaameqeddgdvsktteapaaqsttaspnav	1499
Anopheles arabiensis; XP_040172246.1	eahddeqpgttvssvtsekesdvpvavtvqtameqevdedvskateaaaahsttassnav	1503
Anopheles coluzzii; XP_040218532.1	eahddeqpgttvssvtsekesdvpvavtvqaameqeddedvsktteataahsttassnav	1503
Anopheles darlingi; ETN65512.1	--	1055
Aedes albopictus; XP_029723621.1	--h---kil---assvsvadsdy------ydqeq--aeg---------------------	1309
Drosophila melanogaster; ABW09325.1	ishk-eesa---ke------------eketddekenkvgeie--------lg--depnkv	1359
Aedes aegypti; XP_021710788.1	rdtk-ekpe---avd--------saqkketevkepektdkvi--------vdksaesdkl	1345
Culex quinquefasciatus; EDS38616.1	rdtk-ekpv---tattaaaesaapaakketeekqpektekvt--------tdksaesdkl	1194
Culex pipiens pallens; XP_039443854.1	rdtk-ekpv---tattaaaesaapaakketeekqpektekvt--------tdksaesdkl	1354

Anopheles merus; XP_041782981.1	ddaiiyrvdeeednkpivkptleeevstshavqpthdevkpvedaakpqavdaqet----	1555
Anopheles arabiensis; XP_040172246.1	ddaiiyrvdeeednkpivkptleeevstshavqpthdevkpvedaakpqavdaqet----	1559
Anopheles coluzzii; XP_040218532.1	ddaiiyrvdeeednkpivkptleeevstshavqpthdevkpvedaakpqavdaqet----	1559
Anopheles darlingi; ETN65512.1	--	1055
Aedes albopictus; XP_029723621.1	----smd-------dnnvqtaletiastd-----test-lpsqttiqanrgflkrgsqnt	1352
Drosophila melanogaster; ABW09325.1	dishvll-------kesvqevaekvvvie-----ttve-kkqeeiveattvitqen----	1402
Aedes aegypti; XP_021710788.1	cdtvkdi-------kqevqeviekvai-d-----tmve-kpp--kldtqqpsa-kd----	1384
Culex quinquefasciatus; EDS38616.1	cdtvkda--------qevqeviekiek-d-----tkve-kpp--klepqtspakea----	1233
Culex pipiens pallens; XP_039443854.1	cdtvkda--------qevqeviekiek-d-----tkve-kpp--klepqtspakea----	1393

Anopheles merus; XP_041782981.1	dddnlispvggepehdggfhfpqeddeeqeepifkptlddteqhaavplpaeip-----q	1610
Anopheles arabiensis; XP_040172246.1	dddnlispvggepeqdggfhfpqeddeeqeepifkptlddteqhaavplpaeip-----q	1614
Anopheles coluzzii; XP_040218532.1	dddnlispvggepeqdggfhfpqeddeeqeepifkptlddteqhaavplpaeip-----q	1614
Anopheles darlingi; ETN65512.1	--	1055
Aedes albopictus; XP_029723621.1	ssnipyasfgnnainqslddfiere-----eq-----mkeaeaqaastiqrsyrrfrtnk	1402
Drosophila melanogaster; ABW09325.1	-------------qedlmeqvk-------------------------------------dk	1413
Aedes aegypti; XP_021710788.1	-------------qhde---	1388
Culex quinquefasciatus; EDS38616.1	-------------avde---	1237
Culex pipiens pallens; XP_039443854.1	-------------avde---	1397

Anopheles merus; XP_041782981.1	etvplepvdqi-keqedaeqrttpraeqetmapitskpelpeqere----rvtpairdde	1665
Anopheles arabiensis; XP_040172246.1	etvplepvdqi-keqedaeqgttpraeqetmapstskpelpeqere----rvtpairdde	1669
Anopheles coluzzii; XP_040218532.1	etvplepvdqi-keqedaeqgttpraeqetmapstskpelpeqere----rvtpairdde	1669
Anopheles darlingi; ETN65512.1	--	1055
Aedes albopictus; XP_029723621.1	kkllrdyhstmqtfted--------------qstesledypssviqikldrkqpeesdns	1448
Drosophila melanogaster; ABW09325.1	eeheqkiesgiitekeakksastpee--ketsditsddelpaqladp---ttvppksakd	1468
Aedes aegypti; XP_021710788.1	advlkdlesqiisse--------pei--kkqpetk-------qleqt---peqppvvadd	1428
Culex quinquefasciatus; EDS38616.1	aavlkdlesqiiske--------pev--kaektva-------stvaa---p-veqleddd	1276
Culex pipiens pallens; XP_039443854.1	aavlkdlesqiiske--------pev--kaektva-------stvaa---p-veqleddd	1436

Anopheles merus; XP_041782981.1	qedneieadiqsvavpekkpesvpmydeieeteqqt-vaptkqepvpttvaqeqese---	1721
Anopheles arabiensis; XP_040172246.1	qedneieadiqpvavpekkpepvpmydeieeteqqt-vaptkqepvpttvaqeqese---	1725
Anopheles coluzzii; XP_040218532.1	qkdneieadiqpvavpekkpepvpmydeieeteqqt-vaptkqepvpttvaqeqesk---	1725
Anopheles darlingi; ETN65512.1	--	1055
Aedes albopictus; XP_029723621.1	fedarse------------nrrrpmyslnidey----dtaarr------mtl--trgvam	1484
Drosophila melanogaster; ABW09325.1	redtgsi------------es-pptieeaievevqakqeaqkpvpapeeaikteksplas	1515
Aedes aegypti; XP_021710788.1	eedkksv------------he-----------evivakkvepkvpvpetsildeiwgapq	1465
Culex quinquefasciatus; EDS38616.1	eddkksv------------he-----------evivptkvdpkvpap--svldeiwaapd	1311
Culex pipiens pallens; XP_039443854.1	eddkksv------------he-----------evivptkvdpkvpap--svldeiwaapd	1471

Anopheles merus; XP_041782981.1	---vqpttetagkdevspv--eqdkpeadiepeqpapvt--qsspvd---------ttpa	1765
Anopheles arabiensis; XP_040172246.1	---vqptteaagtdevspv--eqdkpeadsepeqpapvt--qsspvd---------ttpa	1769
Anopheles coluzzii; XP_040218532.1	---vqptteaagndevspv--eqdkpeadsepeqpapvt--qsspvd---------ttpa	1769
Anopheles darlingi; ETN65512.1	--	1055
Aedes albopictus; XP_029723621.1	qrnstpeedsgksdnasgekksasepasnlaitegtprtssdlssee---------kkss	1535
Drosophila melanogaster; ABW09325.1	ketsrpesatgsvk-------------------edteqtkskkspvpsrpeseakdkksp	1556
Aedes aegypti; XP_021710788.1	kaeekleelkqiid-------------------dkakettsdlvav-----------kde	1495
Culex quinquefasciatus; EDS38616.1	kaeekleelkqiid-------------------geakkttselvai-----------kde	1341
Culex pipiens pallens; XP_039443854.1	kaeekleelkqiid-------------------geakkttselvai-----------kde	1501

Anopheles merus; XP_041782981.1	mvqdieqeqdeqehdtepeqvhsddihsttvrva-----aadsaed------eehdeehl	1814
Anopheles arabiensis; XP_040172246.1	ivqdieqeqdeqehgtepeqvhsddihsttvrva-----vadsaed------eehgeehl	1818
Anopheles coluzzii; XP_040218532.1	ivqdieqeqdeqehdtepeqvhsddihsttvrva-----vadsaed------eehdeehl	1818
Anopheles darlingi; ETN65512.1	--	1055
Aedes albopictus; XP_029723621.1	tssdekenkdns--sskssgggkdsa-ces----------------------astln---	1567
Drosophila melanogaster; ABW09325.1	fasgeasrpesvaesvkdeagkaesr-resiakthkdessldkakeqes--rreslaesi	1613
Aedes aegypti; XP_021710788.1	mfkelvdrkeevmdkvkqeiidtdkkaketva-------elakaaenvtvqaaeavakei	1548
Culex quinquefasciatus; EDS38616.1	mvkeiverkeevidvvkaevadvdqkitetvt-------elgksvdq----aagellegl	1390
Culex pipiens pallens; XP_039443854.1	mvkeiverkeevidvvkaevadvdqkitetvt-------elgksvdq----aagellegl	1550

Anopheles merus; XP_041782981.1	ittpaavqaeqdekqqt-t---------------paiassdsevtaveeeqsevqh----	1854
Anopheles arabiensis; XP_040172246.1	nttpaavqaeqdekeqt-t---------------paiassdsdvtpveeehpevqh----	1858
Anopheles coluzzii; XP_040218532.1	ittpaavqaeqdekqqt-t---------------paiaasdsdvtqveeehpdvqh----	1858
Anopheles darlingi; ETN65512.1	--	1055
Aedes albopictus; XP_029723621.1	-errk---	1571
Drosophila melanogaster; ABW09325.1	kp------------esgideksal--------askeasrpesv-tdkskepsrresiaes	1652
Aedes aegypti; XP_021710788.1	vakvdavgedlhkktqqvdekvasevdkiktsveqaaikaesvltsietekkeleklatd	1608
Culex quinquefasciatus; EDS38616.1	ttkadavsadlqqkaqqlddkvaanvdvvktevekaaakadevlttiaaekkeieqlakd	1450
Culex pipiens pallens; XP_039443854.1	atkadavsadlqqkaqqlddkvaanvdvvktevekaaakadevlttiatekkeieqlakd	1610

Anopheles merus; XP_041782981.1	-------------------------------adkvdedeepedd--------vpvvttpq	1875
Anopheles arabiensis; XP_040172246.1	-------------------------------adkvgedeepedd--------vpvattpq	1879
Anopheles coluzzii; XP_040218532.1	-------------------------------adkvgedeepedd--------vpvattpq	1879
Anopheles darlingi; ETN65512.1	--	1055
Aedes albopictus; XP_029723621.1	--	1571
Drosophila melanogaster; ABW09325.1	lkaestkdeksappskeasrpgsvve---svkdetekskepsrresiaesakppiefrev	1709
Aedes aegypti; XP_021710788.1	lkqevs---------------gqidatkkqlddelnkave-----qvqekaksgis----	1644
Culex quinquefasciatus; EDS38616.1	ikeeva---------------eklvetkqqledelnktle-----kttdkakggis----	1486
Culex pipiens pallens; XP_039443854.1	ikeeva---------------eklvetkqqledelnktle-----kttdkakggis----	1646

Anopheles merus; XP_041782981.1	vfvkpvstddeqqveqveqd------------sesdvqsvseh-----------------	1906
Anopheles arabiensis; XP_040172246.1	vfvkpvrtddeqqveqveqd------------seldaqsvseh-----------------	1910
Anopheles coluzzii; XP_040218532.1	vfvkpvstddeqqveqveqd------------sesdaqsvseh-----------------	1910
Anopheles darlingi; ETN65512.1	--	1055
Aedes albopictus; XP_029723621.1	--	1571
Drosophila melanogaster; ABW09325.1	srpesvidgikdesakpesrrdsplaskeasrpesvlesvkdepiksteksrresvaesf	1769
Aedes aegypti; XP_021710788.1	sffggitesiktgiekvadkvetklkdkt-eqvetrlqevtdkieelkgkepveevc---	1700
Culex quinquefasciatus; EDS38616.1	sffggiadgikseiekiadkmetkikdkt-dqvetklqkvtekieelngkqaeeevs---	1542
Culex pipiens pallens; XP_039443854.1	sffggiadgikseiekiadkmetkikdkt-dqvetklqkvtekieelnekqveeevs---	1702
Anopheles merus; XP_041782981.1	-----------------------attpaaifddseqtttadvaddsktvapvateqqtta	1943
Anopheles arabiensis; XP_040172246.1	-----------------------attqaatfddseqtttadvaddnktvapvateqqtta	1947
Anopheles coluzzii; XP_040218532.1	-----------------------attqaatfddseqtttadvaddsktvapvateqqtta	1947
Anopheles darlingi; ETN65512.1	--	1055
Aedes albopictus; XP_029723621.1	---------------------sasmdvqklfiarqrtmpvqiett---------vmra--	1599
Drosophila melanogaster; ABW09325.1	kadstkdekspltskdisrpesavenvmdavgsaersqpesvtas---------rdvsrp	1820
Aedes aegypti; XP_021710788.1	---------vkkevttvpepseqtigfeqsfgy-ertftq--------------------	1730
Culex quinquefasciatus; EDS38616.1	---------qpa--------epeqtgfdapfgy-ertftq--------------------	1564
Culex pipiens pallens; XP_039443854.1	---------qpa--------epeqtgfdapfgy-ertftq--------------------	1724

Anopheles merus; XP_041782981.1	aplqdgekedsekpvfsddasapaad---------ddesqeegteqqtdkppkvpvaqaa 1994
Anopheles arabiensis; XP_040172246.1	apvqdgekedsekpvfsddasapaad---------ddesqeegteqqtdtppkvpvaqaa 1998
Anopheles coluzzii; XP_040218532.1	apvqdgekedsekpvfsddasapaad---------ddesqeegseqqthtpskvpvaqaa 1998
Anopheles darlingi; ETN65512.1	-- 1055
Aedes albopictus; XP_029723621.1	----------------------------qpkhl---------------------------1604
Drosophila melanogaster; ABW09325.1	esvaesekddtdkpesvvesvipasdvve----------iekgaadk------------- 1857
Aedes aegypti; XP_021710788.1	-elrethittldspvtdvdkvvdmkssvnipqhidedkeleeleskq------------- 1776
Culex quinquefasciatus; EDS38616.1	-elrethittldspvseadkmaelksavsipqhidedkeleeietqn------------- 1610
Culex pipiens pallens; XP_039443854.1	-elrethittldspvseadkmaelksavsipqhidedkeleeietqn------------- 1770
Anopheles merus; XP_041782981.1	ddvsedsmyptepvpatnvadvteavlqdsqegeqvehdddeqqtrttlppqvtqdekyq 2054
Anopheles arabiensis; XP_040172246.1	ndvseetmyptepvpatnvadvteavmkdsqeddqvehdddeqqtrttlppqvtqdekyq 2058
Anopheles coluzzii; XP_040218532.1	ddvsedsmyptepvpatnvtdvteavlqdsqedeqvehdddeqqtrttlppqvtqdekyq 2058
Anopheles darlingi; ETN65512.1	-- 1055
Aedes albopictus; XP_029723621.1	-- 1604
Drosophila melanogaster; ABW09325.1	----ekgvfvsle---igkpdspsevisrp--gpvves---------vkpesr--resst 1897
Aedes aegypti; XP_021710788.1	----magsyviee---vkystfeeanlreikeeeeedr---------vtppqkddkksss 1820
Culex quinquefasciatus; EDS38616.1	----msgsymiee---vkyssfedanlrdikeeeeedr---------tsppqkdekksft 1654
Culex pipiens pallens; XP_039443854.1	----msgsymiee---vkyssfedanlrdikeeeeedr---------tsppqkdekksft 1814

Anopheles merus; XP_041782981.1	pq------pqeadaiakptepeqeapmed-------------------------------	2077
Anopheles arabiensis; XP_040172246.1	pq------pqeadaiakptepeqeapmed-------------------------------	2081
Anopheles coluzzii; XP_040218532.1	pq------pqeadaiakptepeqeapmed-------------------------------	2081
Anopheles darlingi; ETN65512.1	--	1055
Aedes albopictus; XP_029723621.1	------------------------rkriks------------------------------	1610
Drosophila melanogaster; ABW09325.1	----eivlpchaedskepsrpeskveclkdesevlkgstrresvaesdkssqpfketsrp	1953
Aedes aegypti; XP_021710788.1	pklepfvvpqra------rtpedvakivanvaevlksdkdleeiip------gf----dp	1864
Culex quinquefasciatus; EDS38616.1	pklepavqptrs------rtpedvakivanvaevlksdkditeiip------gf----dp	1698
Culex pipiens pallens; XP_039443854.1	pklepavqptrs------rtpedvakivanvaevlksdkditeiip------gf----dp	1858

Anopheles merus; XP_041782981.1	----------evefepeqdkqttaapqaveeqdqhttlpstvtsasldeevepeadkeed	2127
Anopheles arabiensis; XP_040172246.1	----------evefepeqekqttaapqaveeqdqhttlpstvssasldeevepeadkeed	2131
Anopheles coluzzii; XP_040218532.1	----------evefepeqekqttaapqaveehdqhttlpstlssasldeevkpeadkeed	2131
Anopheles darlingi; ETN65512.1	--	1055
Aedes albopictus; XP_029723621.1	-------------------------agmirk-----------------------------	1616
Drosophila melanogaster; ABW09325.1	esavgsmkdesmskepsrre--svkdgaaqsret--srpasvaesakdga-------dd-	2001
Aedes aegypti; XP_021710788.1	h---------el----er----klsqgaareedigtvqrmlvtassedgg-------eet	1900
Culex quinquefasciatus; EDS38616.1	k---------el----er----klsqgtareedvstvqrmlvtassedgg-------eet	1734
Culex pipiens pallens; XP_039443854.1	k---------el----er----klsqgtareedvstvqrmlvtassedgg-------eet	1894

Anopheles merus; XP_041782981.1	tmgekvhittarvpamesddkhedqpeadlpepsigvade-----eds------------	2170
Anopheles arabiensis; XP_040172246.1	tmdekvhvttarvpatetddkhedqpeadlsepsigdade-----eds------------	2174
Anopheles coluzzii; XP_040218532.1	tmdekvhvttarvpametddkhedqpeadlpepsigdade-----dds------------	2174
Anopheles darlingi; ETN65512.1	--	1055
Aedes albopictus; XP_029723621.1	--	1616
Drosophila melanogaster; ABW09325.1	---------lkelsrpes-------ttqskeagsikdeksplaseeasrpasvaesvkde	2045
Aedes aegypti; XP_021710788.1	vicpdgtitfsktatpep-------pisgkttpeikvdedave-----------------	1936
Culex quinquefasciatus; EDS38616.1	vicppgtitfskattpep-------ptsgkttpeikvdee--------------------	1767
Culex pipiens pallens; XP_039443854.1	vicppgtitfskattpep-------ptsgkttpeikvdee--------------------	1927

Anopheles merus; XP_041782981.1	------ysttfatpertglddaeqd--------------------------------kkp	2192
Anopheles arabiensis; XP_040172246.1	------ysttfatpertglddaeqa--------------------------------tkp	2196
Anopheles coluzzii; XP_040218532.1	------ysttfatpertglddaeqd--------------------------------tkp	2196
Anopheles darlingi; ETN65512.1	--	1055
Aedes albopictus; XP_029723621.1	--	1616
Drosophila melanogaster; ABW09325.1	aekskeesrresvaeksplpskea---srpasvaesikdea--ekskeesrresvaeksp	2100
Aedes aegypti; XP_021710788.1	------vkpsevrpekpqlpevkqdekaspassgksspdlktsptsieek------dkhe	1984
Culex quinquefasciatus; EDS38616.1	---------seakpekpslpevkpdgktsptssgksspdlktsptsieek------dkhe	1812
Culex pipiens pallens; XP_039443854.1	---------seakpekpslpevkpdgktsptssgksspdlktsptsieek------dkhe	1972

Anopheles merus; XP_041782981.1	veketadsnttvqppspmy---desiepeadsqhkdap----------------------	2227
Anopheles arabiensis; XP_040172246.1	vekemaesnttvqppspvy---desiepeadsqhkdap----------------------	2231
Anopheles coluzzii; XP_040218532.1	veketaesnttvqppspvy---deaiepeadsqhkdap----------------------	2231
Anopheles darlingi; ETN65512.1	--	1055
Aedes albopictus; XP_029723621.1	--	1616
Drosophila melanogaster; ABW09325.1	lpskeasrpa--sv--------aesikdeaekskeesrresva-----------eksplp	2139
Aedes aegypti; XP_021710788.1	lpekvvieak--ktpekitedrkesiaeqyaq-iddsrresaistfserdytkdessild	2041
Culex quinquefasciatus; EDS38616.1	lpeksadakk--tpekkiieerkesiadqydr-iddsrresaistfserdytkdessfvd	1869
Culex pipiens pallens; XP_039443854.1	lpeksadakk--tpekkiieerkesiadqydr-iddsrresaistfserdytkdessfvd	2029

Anopheles merus; XP_041782981.1	-------s------------------------stalpvt------taaaleedevqdndk	2250
Anopheles arabiensis; XP_040172246.1	-------s------------------------stalpvt------taapleedevqdndk	2254
Anopheles coluzzii; XP_040218532.1	-------s------------------------stalpvt------taapleedevqdndk	2254
Anopheles darlingi; ETN65512.1	--	1055
Aedes albopictus; XP_029723621.1	--	1616
Drosophila melanogaster; ABW09325.1	skeasrpasvaesikdeaekskeesrresvaeksplpskeasrpasvaesikdeaekske	2199
Aedes aegypti; XP_021710788.1	erdssrahsisshisdlke--------------------dvadvksvteflkesdkqqev	2081
Culex quinquefasciatus; EDS38616.1	erdssrahsisshisdlke--------------------daadvksvaeflketdekqde	1909
Culex pipiens pallens; XP_039443854.1	erdssrahsisshisdlke--------------------daadvksvaeflketdekqde	2069

Anopheles merus; XP_041782981.1	dgkstt--vqpaliseedaqpsttaasveaeper--dlpsegtvvgda------qdeeqt	2300
Anopheles arabiensis; XP_040172246.1	dgkatt--vqpaliseedsqpsttaasvqgeper--dlpsegtvvsda------qdeeqt	2304
Anopheles coluzzii; XP_040218532.1	dgkatt--vqpaliseedrqpsttaasvqaeper--dlpsegtvvsda------qdeeqt	2304
Anopheles darlingi; ETN65512.1	--	1055
Aedes albopictus; XP_029723621.1	--	1616
Drosophila melanogaster; ABW09325.1	esrresvaeksplpskeasrpasvaesikdeaekskeesrresvaeksp-------lp-s	2251
Aedes aegypti; XP_021710788.1	ftepekqkdsltkkseelsspesitsq---------ktvseksvaaeavlmkseekipqe	2132
Culex quinquefasciatus; EDS38616.1	vg------kattkkadelsspdsvtsq---------ktvseksvadkpltdddka-----	1949
Culex pipiens pallens; XP_039443854.1	vg------katmkkadelsspdsvtsq---------ktvseksvadkpltdddka-----	2109

Anopheles merus; XP_041782981.1	deattaasvsesd---------ekvqptvssttglpvafdave----vgatseeehddae	2347
Anopheles arabiensis; XP_040172246.1	deattpasvpesd---------ekvqptvssttglpvafdave----vgatseeehddae	2351
Anopheles coluzzii; XP_040218532.1	deattpaslpesd---------ekvqptvssttglpvafdave----vgatseeehddae	2351
Anopheles darlingi; ETN65512.1	--	1055
Aedes albopictus; XP_029723621.1	--	1616
Drosophila melanogaster; ABW09325.1	keasrpasvaesikdeaekskeesrresvaeksplp-----skeasrpasvaesikdeae	2306
Aedes aegypti; XP_021710788.1	tqssrpesaashi----------------sskspldnvstspveisgpesa---------	2167
Culex quinquefasciatus; EDS38616.1	--askpesvadek----------------tts--ieakleaekeasrpasaase------	1983
Culex pipiens pallens; XP_039443854.1	--askpesvadek----------------tts--ieakleaekeasrpasaase------	2143

Anopheles merus; XP_041782981.1	qpqt----ddlnad-desseeaeslaavttprap---------------ekvsttaepas	2387
Anopheles arabiensis; XP_040172246.1	qpqt----ddlnad-desseeaeslaavttprap---------------ekvsttaepas	2391
Anopheles coluzzii; XP_040218532.1	qpqt----ddlnad-desseeaeslaavttprap---------------ekvsttaepas	2391
Anopheles darlingi; ETN65512.1	--	1055
Aedes albopictus; XP_029723621.1	--	1616
Drosophila melanogaster; ABW09325.1	kskeetrresvaeksplpskeasrpasvaesikdeaekskeesrresaae-------ksp	2359
Aedes aegypti; XP_021710788.1	----------------iptekspivdtksdtntcekieakeqsrsesaasekm------e	2205
Culex quinquefasciatus; EDS38616.1	---------------kapseksvekeaate-ksptaeaskepsrpesaashvsekaasek	2027
Culex pipiens pallens; XP_039443854.1	---------------kapseksvtaeavk---adekeaskepsrpesaashvsekaasek	2185

Anopheles merus; XP_041782981.1	tpvkevsaeeltpkpe-vyttvaaesdsqvteqp-------------ttvqqeaeqtste	2433
Anopheles arabiensis; XP_040172246.1	ipvkevsaeeltpkpe-vyttvaaesdsqvteqp-------------ttvqqeaeqtste	2437
Anopheles coluzzii; XP_040218532.1	tpvkevsaeeltpkpe-vyttvaaesdsqvteqp-------------ttvqqeaeqtste	2437
Anopheles darlingi; ETN65512.1	--	1055
Aedes albopictus; XP_029723621.1	--	1616
Drosophila melanogaster; ABW09325.1	lpskeas------rpasvaesvkdeadkskeesrresmaesgkaqsikgdqsplkevsrp	2413
Aedes aegypti; XP_021710788.1	dgakeip------rpesvvsnvsekvpsdni----------------e-gqqdskqssrp	2242
Culex quinquefasciatus; EDS38616.1	idvkeas------rpesvashvsekaaseks----------------a-tlekpeessrp	2064
Culex pipiens pallens; XP_039443854.1	idvkeas------rpesvashvsekaaseks----------------a-tlekpeessrp	2222

Anopheles merus; XP_041782981.1	vt---aahadttttskpsqdeiqttaaavdvinrde---eqhhseapeevvsqep---a-	2483
Anopheles arabiensis; XP_040172246.1	vt---aahadttttskpaqdeiqttaaavdvinrde---eqqhseapeevvsqep---a-	2487
Anopheles coluzzii; XP_040218532.1	vt---aahadttttskpsqdeiqttaaavdvinrde---eqhhseapeevvsqep---a-	2487
Anopheles darlingi; ETN65512.1	--	1055
Aedes albopictus; XP_029723621.1	--	1616
Drosophila melanogaster; ABW09325.1	esvaesvkddpvkskepsrresvagsvtadsarddqspleskgasrpesvvdsvkdeaek	2473
Aedes aegypti; XP_021710788.1	esvasykgegavddkepsqpetilscgdvsatkadikpedikevsrpesvasvvnekddt	2302
Culex quinquefasciatus; EDS38616.1	asaasqaseka-----------------------asekaeakeasrsesvashvsekaas	2101
Culex pipiens pallens; XP_039443854.1	asaasqaseka-----------------------asekaeakeasrsesvashvsekaas	2259

Anopheles merus; XP_041782981.1	----------------vtttstaattaqpvaa-------etdgpvressqpatttqedla	2520
Anopheles arabiensis; XP_040172246.1	----------------vtttstaattaqpvaa-------etdgpvressqpatttqedla	2524
Anopheles coluzzii; XP_040218532.1	----------------vtttstaattaqpvaa-------etdgpvressqpatttqedla	2524
Anopheles darlingi; ETN65512.1	--	1055
Aedes albopictus; XP_029723621.1	--	1616
Drosophila melanogaster; ABW09325.1	qesrresktesvippkakddkspkevlqpvsm-tetiredadqpmkpsqaes--rresia	2530
Aedes aegypti; XP_021710788.1	ekkaatks-e-----------sp--ashptsietektsaetkessrpesvas--l---vs	2343
Culex quinquefasciatus; EDS38616.1	eksvsek------------------------aaveqaesktdepsrpesaas--h---vs	2132
Culex pipiens pallens; XP_039443854.1	eksvsek------------------------aaveqaesktdepsrpesaas--h---vs	2290

Anopheles merus; XP_041782981.1	sstvaapvaaqddkldekvdeq-----avvpavpekpqpvsddseeqqeavkptftede-	2574
Anopheles arabiensis; XP_040172246.1	sstvaapvaahddkldekvdeq-----avvpavpekpqpvsddseeqqeavkptftede-	2578
Anopheles coluzzii; XP_040218532.1	sstvaapvaaqddkldekvdeq-----avvpavpekpqpvsddseeqqeavkptftede-	2578
Anopheles darlingi; ETN65512.1	--	1055
Aedes albopictus; XP_029723621.1	--	1616
Drosophila melanogaster; ABW09325.1	esikassprdeksplaskeasrpgsvaesikydldkpqiikddksteh--srresledks	2588
Aedes aegypti; XP_021710788.1	ek-------alpektdd---	2353
Culex quinquefasciatus; EDS38616.1	ek-------aveskltteqqsrpdsaashvseketap--------kep--srlesaasqv	2175
Culex pipiens pallens; XP_039443854.1	ek-------aveskltteqqsrpdsaashvseketap--------kep--srpesaasqv	2333

Anopheles merus; XP_041782981.1	-hddqqkvqepevvgvsees-------------------------sssspakptqqpetv	2608
Anopheles arabiensis; XP_040172246.1	-hddqqkvpepevvgvsees---------------------------sspakptqqpetv	2610
Anopheles coluzzii; XP_040218532.1	-hddqqkvqepevvgvsees---------------------------sslakptqqpetv	2610
Anopheles darlingi; ETN65512.1	--	1055
Aedes albopictus; XP_029723621.1	--	1616
Drosophila melanogaster; ABW09325.1	avtseksvsrplsvasdheaavaied-----------------dakssispkdksrpgfv	2631
Aedes aegypti; XP_021710788.1	----skevsrpesaashisdklvseqketipsekvetkespraesvasqetkehsrpesv	2409
Culex quinquefasciatus; EDS38616.1	aekvdvkvsrpesaashvsektsadk---------------------lsepkeasrpasa	2214
Culex pipiens pallens; XP_039443854.1	aekvdvkvsrpesaashvsektsadk---------------------lsepkeasrpasa	2372

Anopheles merus; XP_041782981.1	gpsygapgqhydtgyghmpphyppssyeddygeeedpaafgpgtcryggklyvsaqqipr	2668
Anopheles arabiensis; XP_040172246.1	gpsygapgqhydtgyghmpphyppssyeddygeeedpaafgpgtcryggklyvsaqqipr	2670
Anopheles coluzzii; XP_040218532.1	gpsygapgqhydtgyghmpphyppssyeddygeeedpaafgpgtcryggklyvsaqqipr	2670
Anopheles darlingi; ETN65512.1	--	1055
Aedes albopictus; XP_029723621.1	--	1616
Drosophila melanogaster; ABW09325.1	aetvsspieeatmefskievve------------ksslals-lqggsggklqtdsspvdv	2678
Aedes aegypti; XP_021710788.1	lsqks-------------------------------dk----pd-------n--------	2419
Culex quinquefasciatus; EDS38616.1	lsdka-------------------------------as----dksaesgkve--------	2231
Culex pipiens pallens; XP_039443854.1	lsdka-------------------------------as----dksvesgkve--------	2389

Anopheles merus; XP_041782981.1	ddpcdfcfcfrsdiiclqqscpppisgcneepiagfccpryecpvsmatvlnvtt-----	2723
Anopheles arabiensis; XP_040172246.1	ddpcdfcfcfrsdiiclqqscpppisgcneepiagfccpryecpvsmatvlnvtt-----	2725
Anopheles coluzzii; XP_040218532.1	ddpcdfcfcfrsdiiclqqscpppisgcneepiagfccpryecpvsmatvlnvtt-----	2725
Anopheles darlingi; ETN65512.1	--	1055
Aedes albopictus; XP_029723621.1	--	1616
Drosophila melanogaster; ABW09325.1	-aegdfshavas----vs-t----------------vtptltkpaelaqigaaktvsspl	2716
Aedes aegypti; XP_021710788.1	--	2419
Culex quinquefasciatus; EDS38616.1	--	2231
Culex pipiens pallens; XP_039443854.1	--	2389

Anopheles merus; XP_041782981.1	stttttttlpphflshaykghvqkrgcqiqgkp--ynvgetvasasgpcmrctcggdgq-	2780
Anopheles arabiensis; XP_040172246.1	stttttttlpphflshaykghvqkrgcqiqgkp--ynvgetvasasgpcmrctcggdgq-	2782
Anopheles coluzzii; XP_040218532.1	stttttttlpphflshaykghvqkrgcqiqgkp--ynvgetvasasgpcmrctcggdgq-	2782
Anopheles darlingi; ETN65512.1	--	1055
Aedes albopictus; XP_029723621.1	--	1616
Drosophila melanogaster; ABW09325.1	dealrtpsapehisrads-----paecaseeiasqdkspqvlkessrpawvaeskddaaq	2771
Aedes aegypti; XP_021710788.1	-------------------------------------kqeamkgssrpesvasdvgeki-	2441
Culex quinquefasciatus; EDS38616.1	-------------------------------------adkdvkeasrpesaasvvgdke-	2253
Culex pipiens pallens; XP_039443854.1	-------------------------------------adkdvkeasrpesaasvigdke-	2411

Anopheles merus; XP_041782981.1	m-------------------------qcepkac------------spepmlqqmiavaaa	2803
Anopheles arabiensis; XP_040172246.1	m-------------------------qcepkac------------spepmlqqmiavaaa	2805
Anopheles coluzzii; XP_040218532.1	m-------------------------qcepkac------------spepmlqqmiavaaa	2805
Anopheles darlingi; ETN65512.1	--	1055
Aedes albopictus; XP_029723621.1	--	1616
Drosophila melanogaster; ABW09325.1	lkssvedlrspvasteisrpasagetasspieeapkdfaefeqaeka--vlpltielkgn	2829
Aedes aegypti; XP_021710788.1	-------------tsee-------------------kseeikdgsrpesvasqivettsh	2469
Culex quinquefasciatus; EDS38616.1	-------------ekepsrpv----------saapsekldvkeasrpesaashtsekdda	2290
Culex pipiens pallens; XP_039443854.1	-------------ekepsrpv----------saapsekldvkeasrpesaashtsekd--	2446

Anopheles merus; XP_041782981.1	---r--------------	2804
Anopheles arabiensis; XP_040172246.1	---r--------------	2806
Anopheles coluzzii; XP_040218532.1	---r--------------	2806
Anopheles darlingi; ETN65512.1	--	1055
Aedes albopictus; XP_029723621.1	--	1616
Drosophila melanogaster; ABW09325.1	lptlsspvdvahgdfpqtstptssptvasvqp-aelskvdiekta-----sspideapks	2883
Aedes aegypti; XP_021710788.1	---------------------------------rvakdvdekqtarsesvashvseksst	2496
Culex quinquefasciatus; EDS38616.1	lvais--ddkkdgkepsr--p---esvashvsekaeektdvkeasrpesvashvsekpaa	2343
Culex pipiens pallens; XP_039443854.1	--------dkkdgkepsr--p---esvashvsekaeektdvkeasrpesvashvsekpaa	2493

Anopheles merus; XP_041782981.1	----------rr--	2806
Anopheles arabiensis; XP_040172246.1	----------rr--	2808
Anopheles coluzzii; XP_040218532.1	----------rr--	2808
Anopheles darlingi; ETN65512.1	--	1055
Aedes albopictus; XP_029723621.1	--	1616
Drosophila melanogaster; ABW09325.1	li--gcpaeerpespaesa--kdaae--svekskdasrppsvvestkadstkgdispspe	2937
Aedes aegypti; XP_021710788.1	ekepaameasrpesvtslisdkdet---kkaeskeasrpesavshisek-eevksss---	2549
Culex quinquefasciatus; EDS38616.1	ek--aethasrpesvashvsekdateeqtkteakeasrpesvashvsek-issdksp---	2397
Culex pipiens pallens; XP_039443854.1	ek--aethasrpesvashvsekdateeqtkteakeasrpesvashvsek-issdksp---	2547

Anopheles merus; XP_041782981.1	--	2806
Anopheles arabiensis; XP_040172246.1	--	2808
Anopheles coluzzii; XP_040218532.1	--	2808
Anopheles darlingi; ETN65512.1	--	1055
Aedes albopictus; XP_029723621.1	--	1616
Drosophila melanogaster; ABW09325.1	svlegpkddvekskessrppsvsasitgdstk----------dvsrpasvvesvkdehdk	2987
Aedes aegypti; XP_021710788.1	-svekdvaktdeakeasrpdsvashvsekdake----------asrpasvashvseqldt	2598
Culex quinquefasciatus; EDS38616.1	-lvd-kveekptgkeesrpesvashvsekaasdksshldkpeessrpasaasqasekaas	2455
Culex pipiens pallens; XP_039443854.1	-lvd-kveekstgkeesrpesvashvsekaasdksshldkpeessrpasaasqasekatf	2605

Anopheles merus; XP_041782981.1	--	2806
Anopheles arabiensis; XP_040172246.1	--	2808
Anopheles coluzzii; XP_040218532.1	--	2808
Anopheles darlingi; ETN65512.1	--	1055
Aedes albopictus; XP_029723621.1	--	1616
Drosophila melanogaster; ABW09325.1	aesrresiakvesvideagksdsksssqdsqkdekstlaskeasrresvvesskdda-ek	3046
Aedes aegypti; XP_021710788.1	-----------------------------------ykletkeasrpesrtshtseiidak	2623
Culex quinquefasciatus; EDS38616.1	-----------------------------------ekpde--------------------	2460
Culex pipiens pallens; XP_039443854.1	-----------------------------------ekpde--------------------	2610

Anopheles merus; XP_041782981.1	--	2806
Anopheles arabiensis; XP_040172246.1	--	2808
Anopheles coluzzii; XP_040218532.1	--	2808
Anopheles darlingi; ETN65512.1	--	1055
Aedes albopictus; XP_029723621.1	--	1616
Drosophila melanogaster; ABW09325.1	sesrpesviasgepvpreskspldskdtsrpgsmvesvtaede--kseqqsrresvaesv	3104
Aedes aegypti; XP_021710788.1	lkeepklvetkd--v---aqpdakmeetsrpesvvshasekvstdkateavrpdsteshi	2678
Culex quinquefasciatus; EDS38616.1	-------------------------keasrpesvvshvsekaaseksatlekp-------	2488
Culex pipiens pallens; XP_039443854.1	-------------------------keasrpesvvshvsekaaseksatlekp-------	2638

Anopheles merus; XP_041782981.1	--	2806
Anopheles arabiensis; XP_040172246.1	--	2808
Anopheles coluzzii; XP_040218532.1	--	2808
Anopheles darlingi; ETN65512.1	--	1055
Aedes albopictus; XP_029723621.1	--	1616
Drosophila melanogaster; ABW09325.1	kadtkkdgksqeasrpssvdellkdddekqesrrqsitgshkamstmgdespmdkadksk	3164
Aedes aegypti; XP_021710788.1	--gakattmqeetsrpmsvasnv------------------------sekld-tkkeepk	2711
Culex quinquefasciatus; EDS38616.1	----------eessrptsaasqa------------------------setap-sekqdak	2513
Culex pipiens pallens; XP_039443854.1	----------eessrptsaasqa------------------------setap-sekqdak	2663

Anopheles merus; XP_041782981.1	--	2806
Anopheles arabiensis; XP_040172246.1	--	2808
Anopheles coluzzii; XP_040218532.1	--	2808
Anopheles darlingi; ETN65512.1	--	1055
Aedes albopictus; XP_029723621.1	--	1616
Drosophila melanogaster; ABW09325.1	epsrpesvaesikhentk------------------------------deespl------	3188
Aedes aegypti; XP_021710788.1	dssrpesviseatsdkfdek----------q------------------kdtlqsvasqe	2743
Culex quinquefasciatus; EDS38616.1	easrpesaashvsekaaseksamlekpeekeasrpasatsqvsekatsekdtpaektdgk	2573
Culex pipiens pallens; XP_039443854.1	easrpesaashvsekaaseksamlekpeekeasrpasatsqvsekatsekntpaektdgk	2723

Anopheles merus; XP_041782981.1	--	2806
Anopheles arabiensis; XP_040172246.1	--	2808
Anopheles coluzzii; XP_040218532.1	--	2808
Anopheles darlingi; ETN65512.1	--	1055
Aedes albopictus; XP_029723621.1	--	1616
Drosophila melanogaster; ABW09325.1	-gsrrdsvaesik-sditkgeksplpskevsrpesvvgsikdekaesrresvaesvkpes	3246
Aedes aegypti; XP_021710788.1	ivktsekveseliasqvsektsvkseveessrpesvashlsekas---------------	2788
Culex quinquefasciatus; EDS38616.1	easrsesvashvsekaasdkaeekpaskeasrpesvashvsekaa---------------	2618
Culex pipiens pallens; XP_039443854.1	easrsesvashvsekaasdkaeekp-----------------------------------	2748

Anopheles merus; XP_041782981.1	--	2806
Anopheles arabiensis; XP_040172246.1	--	2808
Anopheles coluzzii; XP_040218532.1	--	2808
Anopheles darlingi; ETN65512.1	--	1055
Aedes albopictus; XP_029723621.1	--	1616
Drosophila melanogaster; ABW09325.1	skdatsappskehsrpesvlgslkdegdkttsrrvsvadsikdeksllvsqeasrpesea	3306
Aedes aegypti; XP_021710788.1	sdkvei----kdssrpesvashisektssad------------aks--------------	2818
Culex quinquefasciatus; EDS38616.1	sdkaeekpaskeasrpesvashvsekaasek------------satlekp----------	2656
Culex pipiens pallens; XP_039443854.1	--------askeasrpesvashvsekaasek------------satlekp----------	2778

Anopheles merus; XP_041782981.1	--	2806
Anopheles arabiensis; XP_040172246.1	--	2808
Anopheles coluzzii; XP_040218532.1	--	2808
Anopheles darlingi; ETN65512.1	--	1055
Aedes albopictus; XP_029723621.1	--	1616
Drosophila melanogaster; ABW09325.1	eslkdaaapsqetsrpesvtesvkdgkspvaskeasrpasvaenakdsadeskeqrpesl	3366
Aedes aegypti; XP_021710788.1	--------------------------------paasrpdsvashssdkvaekedikkeel	2846
Culex quinquefasciatus; EDS38616.1	--------------------------------eessrpasaasqaseka-----------	2673
Culex pipiens pallens; XP_039443854.1	--------------------------------eessrpasatsqaseka-----------	2795

Anopheles merus; XP_041782981.1	--	2806
Anopheles arabiensis; XP_040172246.1	--	2808
Anopheles coluzzii; XP_040218532.1	--	2808
Anopheles darlingi; ETN65512.1	--	1055
Aedes albopictus; XP_029723621.1	--	1616
Drosophila melanogaster; ABW09325.1	pqskagsikdeksplaskdeaekskeesrresvaeqfplvskevsrpasvaesvkdeae-	3425
Aedes aegypti; XP_021710788.1	haat---------------------------------qkevkeaqrpesasshv------	2867
Culex quinquefasciatus; EDS38616.1	-pse---------------------------------kpdakeasrpdsaashvsekaas	2699
Culex pipiens pallens; XP_039443854.1	-pse---------------------------------kpdakeasrpdsaashvsekaas	2821

Anopheles merus; XP_041782981.1	--	2806
Anopheles arabiensis; XP_040172246.1	--	2808
Anopheles coluzzii; XP_040218532.1	--	2808
Anopheles darlingi; ETN65512.1	--	1055
Aedes albopictus; XP_029723621.1	--	1616
Drosophila melanogaster; ABW09325.1	-kskeesplmskeasrpasvagsvkdeaekskeesrr-----esvaek----------sp	3469
Aedes aegypti; XP_021710788.1	-----sdkpevkdtsrpesvashasekgpsdkidikdet--kglvqpmlpvsqektdskq	2920
Culex quinquefasciatus; EDS38616.1	eksmtldkpeekeasrpasaashvsekaaseksatlekpddkessrps-------salsq	2752
Culex pipiens pallens; XP_039443854.1	eksttldkpeekeasrpasaashvsekaaseksatlekpddkessrps-------salsq	2874

Anopheles merus; XP_041782981.1	--	2806
Anopheles arabiensis; XP_040172246.1	--	2808
Anopheles coluzzii; XP_040218532.1	--	2808
Anopheles darlingi; ETN65512.1	--	1055
Aedes albopictus; XP_029723621.1	--	1616
Drosophila melanogaster; ABW09325.1	lpskeasrpasvaesvkdeadkskeesrresgaeksplaskeasrpasvaesikdeaeks	3529
Aedes aegypti; XP_021710788.1	deknelsrpescashvsekemset---------tetklgsmevaqlksias----qasek	2967
Culex quinquefasciatus; EDS38616.1	adekeasrpesaashvsekvtseg---------kpedk---pvsrpesmvg----etkps	2796
Culex pipiens pallens; XP_039443854.1	adekeasrpesaashvsemvtseg---------khedk---pvsrpesmdg----etkps	2918

Anopheles merus; XP_041782981.1	--	2806
Anopheles arabiensis; XP_040172246.1	--	2808
Anopheles coluzzii; XP_040218532.1	--	2808
Anopheles darlingi; ETN65512.1	--	1055
Aedes albopictus; XP_029723621.1	--	1616
Drosophila melanogaster; ABW09325.1	keesrresvaeksplpskeasrptsvaesvkdeaekskeesrrdsvaeksplaskeasrp	3589
Aedes aegypti; XP_021710788.1	----tstevietvkvvskepsrpesvashtseh-----aetk---------sdakessrs	3009
Culex quinquefasciatus; EDS38616.1	----dkaedkpdgkqdakeasrpessashvsekaasdktedk---------qdakeasrp	2843
Culex pipiens pallens; XP_039443854.1	----dkvedkpedkqdakeasrpv------sekaasdktedk---------qdakeasrp	2959

Anopheles merus; XP_041782981.1	--	2806
Anopheles arabiensis; XP_040172246.1	--	2808
Anopheles coluzzii; XP_040218532.1	--	2808
Anopheles darlingi; ETN65512.1	--	1055
Aedes albopictus; XP_029723621.1	--	1616
Drosophila melanogaster; ABW09325.1	asvaesvqdeaekskeesrresvaeksplaskeasrpasvaesikdeaekskeesrresv	3649
Aedes aegypti; XP_021710788.1	asvashvsdks--------------epkpevedasrpesvashtsdkehstekeskstye	3055
Culex quinquefasciatus; EDS38616.1	esvashvsekaasdkspla---dkaeetsaskeasrpesvashvsekaasekst------	2894
Culex pipiens pallens; XP_039443854.1	esvashvsekaasdkppla---dkaeetsaskeasrpesvashvsekaasekst------	3010

Anopheles merus; XP_041782981.1	--	2806
Anopheles arabiensis; XP_040172246.1	--	2808
Anopheles coluzzii; XP_040218532.1	--	2808
Anopheles darlingi; ETN65512.1	--	1055
Aedes albopictus; XP_029723621.1	--	1616
Drosophila melanogaster; ABW09325.1	aeksplaskeasrptsvaesvkdeaekskeessrdsvaeksplaskeasrpasvaesvqd	3709
Aedes aegypti; XP_021710788.1	ikgqlpeskeastlesatnivtgkvptdd-------------gk----------------	3086
Culex quinquefasciatus; EDS38616.1	---qlekpeessrpvsaashvsekavsek-------------adametsrpesvashvse	2938
Culex pipiens pallens; XP_039443854.1	---qlekpeessrpvsaashvsekavsek-------------adaketsrpesvvshvse	3054

Anopheles merus; XP_041782981.1	--	2806
Anopheles arabiensis; XP_040172246.1	--	2808
Anopheles coluzzii; XP_040218532.1	--	2808
Anopheles darlingi; ETN65512.1	--	1055
Aedes albopictus; XP_029723621.1	--	1616
Drosophila melanogaster; ABW09325.1	eaekskeesrresvaeksplaskeasrpasvaesvkddaekskeesrres---------v	3760
Aedes aegypti; XP_021710788.1	na----------etnithasvnkedskpasilsqidekalssvkdtarpqekepsvtdsq	3136
Culex quinquefasciatus; EDS38616.1	ke----------saevqiktedketsrpasvashvsekaasek----------------s	2972
Culex pipiens pallens; XP_039443854.1	ke----------saevqiktedkeasrpasvashvsekaasek----------------s	3088

Anopheles merus; XP_041782981.1	--	2806
Anopheles arabiensis; XP_040172246.1	--	2808
Anopheles coluzzii; XP_040218532.1	--	2808
Anopheles darlingi; ETN65512.1	--	1055
Aedes albopictus; XP_029723621.1	--	1616
Drosophila melanogaster; ABW09325.1	aeksplaskeasrpasvaesvkdeaekskeesrresvaeksplpskeasrptsvaesvkd	3820
Aedes aegypti; XP_021710788.1	cgkeqplkesdsrptsaashvsekv-----------stdktp--ekeesrpasavshvse	3183
Culex quinquefasciatus; EDS38616.1	atlekpddkepsrpesaashvseka-----------asdkakpdekeasrpesvvsqase	3021
Culex pipiens pallens; XP_039443854.1	vtleksddkelsrpesaashvseka-----------asdkakpdekeasrpesvvsqase	3137

Anopheles merus; XP_041782981.1	--	2806
Anopheles arabiensis; XP_040172246.1	--	2808
Anopheles coluzzii; XP_040218532.1	--	2808
Anopheles darlingi; ETN65512.1	--	1055
Aedes albopictus; XP_029723621.1	--	1616
Drosophila melanogaster; ABW09325.1	eaekskeesrresvaeksslaskkasrpasvaesvkdeaekskeesrresvaeksplask	3880
Aedes aegypti; XP_021710788.1	sapsdk-----ldiik---aedkdywrpassashvsekss----------tidavvidqk	3225
Culex quinquefasciatus; EDS38616.1	kv----------svad---kseekvsrpesqasavdektv--------ektvselqvdsk	3060
Culex pipiens pallens; XP_039443854.1	ka----------svad---kseekvsrpesqasavdektv--------ektvselqvdsk	3176

Anopheles merus; XP_041782981.1	--	2806
Anopheles arabiensis; XP_040172246.1	--	2808
Anopheles coluzzii; XP_040218532.1	--	2808
Anopheles darlingi; ETN65512.1	--	1055
Aedes albopictus; XP_029723621.1	--	1616
Drosophila melanogaster; ABW09325.1	easrpasvaesvkdeaekskeesrresvaeksplpskeasrptsvaesvkdeadkskees	3940
Aedes aegypti; XP_021710788.1	espapistvsptiekeqkepsrsqs-aasek-----------------------------	3255
Culex quinquefasciatus; EDS38616.1	ds-rpasaashtsdkaepasekiqv-assekapeqkpeak----------d---------	3099
Culex pipiens pallens; XP_039443854.1	ds-rpasaashtsdkaepasekiqv-vssektpeqkleak----------d---------	3215

Anopheles merus; XP_041782981.1	--	2806
Anopheles arabiensis; XP_040172246.1	--	2808
Anopheles coluzzii; XP_040218532.1	--	2808
Anopheles darlingi; ETN65512.1	--	1055
Aedes albopictus; XP_029723621.1	--	1616
Drosophila melanogaster; ABW09325.1	rresgaeksplasmeasrptsvaesvkdetekskeesrresvteksplpskeasrptsva	4000
Aedes aegypti; XP_021710788.1	------------veetsrpvsvasd--------------kaktveskaddkeasrptsaa	3289
Culex quinquefasciatus; EDS38616.1	-----dkpsaddktsqdrpassashvsdk------eseketarkesiisekeasrptsva	3148
Culex pipiens pallens; XP_039443854.1	-----------dktsqdrpassashvsdk------eseketarkesiisekeasrptsva	3258

Anopheles merus; XP_041782981.1	--	2806
Anopheles arabiensis; XP_040172246.1	--	2808
Anopheles coluzzii; XP_040218532.1	--	2808
Anopheles darlingi; ETN65512.1	--	1055
Aedes albopictus; XP_029723621.1	--	1616
Drosophila melanogaster; ABW09325.1	esvkdeaekskeesrresvaeksplaskessrpasvaesikde------aegtkqesrre	4054
Aedes aegypti; XP_021710788.1	shksekdsh-kaef------------ekdssrpassashasekgsvlqetestkte----	3332
Culex quinquefasciatus; EDS38616.1	shvsekdaavkves------------ekeasrpassashvse------------------	3178
Culex pipiens pallens; XP_039443854.1	shvsekdaavkves------------ekeasrpassashvse------------------	3288

Anopheles merus; XP_041782981.1	--	2806
Anopheles arabiensis; XP_040172246.1	--	2808
Anopheles coluzzii; XP_040218532.1	--	2808
Anopheles darlingi; ETN65512.1	--	1055
Aedes albopictus; XP_029723621.1	--	1616
Drosophila melanogaster; ABW09325.1	smpesgkaesikgdqsslasketsrpdsvvesvkdetekpegsaidksqvasrpesvavs	4114
Aedes aegypti; XP_021710788.1	emdiav-------kveskddkdpsrsasvashvse-tdkev-----dektqsrpdsaash	3379
Culex quinquefasciatus; EDS38616.1	----------------kedakvpsrpestashvsettdkkv-----nekepsrpesaash	3217
Culex pipiens pallens; XP_039443854.1	----------------kedakvpsrpestashvsettdkkm-----dekepsrpesaash	3327

Anopheles merus; XP_041782981.1	--	2806
Anopheles arabiensis; XP_040172246.1	--	2808
Anopheles coluzzii; XP_040218532.1	--	2808
Anopheles darlingi; ETN65512.1	--	1055
Aedes albopictus; XP_029723621.1	--	1616
Drosophila melanogaster; ABW09325.1	akdeksplhsrpesvadkspdaskeasrslsvaetasspieegprsiadlslplnltge-	4173
Aedes aegypti; XP_021710788.1	gpsdksvpkd------------skpddkelslpesttse---------tvakpldassid	3418
Culex quinquefasciatus; EDS38616.1	apsdkspakd------------lkvdekiasrpesaaes---------vvs---dktsev	3253
Culex pipiens pallens; XP_039443854.1	apsdkspakd------------lkvdekiasrpesaaes---------vvs---nkasev	3363

Anopheles merus; XP_041782981.1	--	2806
Anopheles arabiensis; XP_040172246.1	--	2808
Anopheles coluzzii; XP_040218532.1	--	2808
Anopheles darlingi; ETN65512.1	--	1055
Aedes albopictus; XP_029723621.1	--	1616
Drosophila melanogaster; ABW09325.1	---akgklptlss-----------pidvaegdflevkae-----ssprpavlskpaefsq	4214
Aedes aegypti; XP_021710788.1	kligegdfsrpespascssdkv-epskepkstfvekdlarpdsscsyhgehipdekpfsr	3477
Culex quinquefasciatus; EDS38616.1	kptadgdfsrpespascssdkivapskepesgffekdlarrdsndsfhgepipdektfsr	3313
Culex pipiens pallens; XP_039443854.1	kptadgdfsrpespascssdkivapskepesgffekdlarrdsndsfhgepipdektfsr	3423

Anopheles merus; XP_041782981.1	--	2806
Anopheles arabiensis; XP_040172246.1	--	2808
Anopheles coluzzii; XP_040218532.1	--	2808
Anopheles darlingi; ETN65512.1	--	1055
Aedes albopictus; XP_029723621.1	--	1616
Drosophila melanogaster; ABW09325.1	pdtghtastpvdeaspvleeievveqhttsgvgatgataetdlldltetksetvtkqset	4274
Aedes aegypti; XP_021710788.1	pespds-------------------------------caddklnevetqpaekq------	3500
Culex quinquefasciatus; EDS38616.1	pespds-------------------------------caedkkeqivetkpetvtkveek	3342
Culex pipiens pallens; XP_039443854.1	pespds-------------------------------caedkkelivetkpetvtkveek	3452

Anopheles merus; XP_041782981.1	--	2806
Anopheles arabiensis; XP_040172246.1	--	2808
Anopheles coluzzii; XP_040218532.1	--	2808
Anopheles darlingi; ETN65512.1	--	1055
Aedes albopictus; XP_029723621.1	--	1616
Drosophila melanogaster; ABW09325.1	tlfetltskveskvevlessvkqveekvqtsvkqaettvtdsleqltkksseqlteiksv	4334
Aedes aegypti; XP_021710788.1	vt---------gdda-----vkqvi----dttk--dvkked---------------mesp	3525
Culex quinquefasciatus; EDS38616.1	it--aseqkvetkea-----iqevkkeqiteskqattseqk---------------dvsv	3380
Culex pipiens pallens; XP_039443854.1	it--aseqkvetkea-----iqevkkeqvteskqattseqk---------------dvsv	3490

Anopheles merus; XP_041782981.1	--	2806
Anopheles arabiensis; XP_040172246.1	--	2808
Anopheles coluzzii; XP_040218532.1	--	2808
Anopheles darlingi; ETN65512.1	--	1055
Aedes albopictus; XP_029723621.1	--	1616
Drosophila melanogaster; ABW09325.1	ldtnfeevakivadvakvlksdkditdi----------------ipdfd------erqle	4372
Aedes aegypti; XP_021710788.1	evkefsrp-----dspascgdeteltsktpessipekhlaredsvqsyhgepipeekqfs	3580
Culex quinquefasciatus; EDS38616.1	eekdfsrp-----dspascgdekeltskapessvpekdlarpdsaasyhgepipgekqfs	3435
Culex pipiens pallens; XP_039443854.1	eekdfsrp-----dspascgdekeltskapessvpekdlarpdsaasyhgepipgekqfs	3545

Anopheles merus; XP_041782981.1	--	2806
Anopheles arabiensis; XP_040172246.1	--	2808
Anopheles coluzzii; XP_040218532.1	--	2808
Anopheles darlingi; ETN65512.1	--	1055
Aedes albopictus; XP_029723621.1	--	1616
Drosophila melanogaster; ABW09325.1	eklkstadteee---sdkstrdeksl-----------------eisvkveieseksspdq	4412
Aedes aegypti; XP_021710788.1	rpdspasggedetpapvvk-----------------------skessrpesaashisgkd	3617
Culex quinquefasciatus; EDS38616.1	rpespasctedepaeaekvsrpesavshvsekdsksqdeskdvkaesrpesvashvsekd	3495
Culex pipiens pallens; XP_039443854.1	rpespasctedepteaekvsrpesavshvsekdskpqdeskdvkaesrpesvashvsekd	3605

Anopheles merus; XP_041782981.1	--	2806
Anopheles arabiensis; XP_040172246.1	--	2808
Anopheles coluzzii; XP_040218532.1	--	2808
Anopheles darlingi; ETN65512.1	--	1055
Aedes albopictus; XP_029723621.1	--	1616
Drosophila melanogaster; ABW09325.1	ksgpisieekdkieqsekaqlrqgiltssrpesvasq-------------------pesv	4453
Aedes aegypti; XP_021710788.1	ks-pvgseakes-------------l----------------------------------	3629
Culex quinquefasciatus; EDS38616.1	skspapveakev-------------keasrpesvvshvsekdtkpqeeskdtkesrpesv	3542
Culex pipiens pallens; XP_039443854.1	skspapveakev-------------keasrpesvvshvsekdtksqeeskdtkesrpesv	3652

Anopheles merus; XP_041782981.1	--	2806
Anopheles arabiensis; XP_040172246.1	--	2808
Anopheles coluzzii; XP_040218532.1	--	2808
Anopheles darlingi; ETN65512.1	--	1055
Aedes albopictus; XP_029723621.1	--	1616
Drosophila melanogaster; ABW09325.1	pspsqsaashehkevelseshkaekssrpesvasqvsekdmkts----------rpasst	4503
Aedes aegypti; XP_021710788.1	--tl-----------------nekdts---------------------------rptsaa	3643
Culex quinquefasciatus; EDS38616.1	ashvs---ekdskspapveskevkdas---------------------------rpesva	3572
Culex pipiens pallens; XP_039443854.1	ashvs---ekdskspaavestvakeasrpesvashvsekdtksqeeskdtktesrpesva	3709

Anopheles merus; XP_041782981.1	--	2806
Anopheles arabiensis; XP_040172246.1	--	2808
Anopheles coluzzii; XP_040218532.1	--	2808
Anopheles darlingi; ETN65512.1	--	1055
Aedes albopictus; XP_029723621.1	--	1616
Drosophila melanogaster; ABW09325.1	sqfstkegdeettes----llhslttt--etvetk--------qmeekssfesvstsvtk	4549
Aedes aegypti; XP_021710788.1	shasdksgatea-------kmes-------------------------------------	3659
Culex quinquefasciatus; EDS38616.1	shvsekdskssaqddtkdtkaqsrpesaashvsekdskspadkpkseesrpesaishv--	3630
Culex pipiens pallens; XP_039443854.1	shvsekdskssaqddtkdtkaqsrpesaashvsekdskspadkpkseasrpesaashv--	3767

Anopheles merus; XP_041782981.1	--	2806
Anopheles arabiensis; XP_040172246.1	--	2808
Anopheles coluzzii; XP_040218532.1	--	2808
Anopheles darlingi; ETN65512.1	--	1055
Aedes albopictus; XP_029723621.1	--	1616
Drosophila melanogaster; ABW09325.1	stvlssqstvqlreestseslss-------slkvedssrreslssllaek---ggiat-n	4598
Aedes aegypti; XP_021710788.1	----rresatqeerps--salsdhadnaqeekifmkesrskslsavmsstpqrigfegsa	3713
Culex quinquefasciatus; EDS38616.1	----seksvdqsrres--aalsdhed--eaekvvmketrskslsavmatttqrsgfegsa	3682
Culex pipiens pallens; XP_039443854.1	----seksvdqsrres--aalsdhed--eaekvvmketrskslsavmatttqrsgfegsa	3819

Anopheles merus; XP_041782981.1	--	2806
Anopheles arabiensis; XP_040172246.1	--	2808
Anopheles coluzzii; XP_040218532.1	--	2808
Anopheles darlingi; ETN65512.1	--	1055
Aedes albopictus; XP_029723621.1	--	1616
Drosophila melanogaster; ABW09325.1	ts-lkedtsasasqleellvqseecsse---sivseiqtsiaqksnkeikda-----ret	4649
Aedes aegypti; XP_021710788.1	lgvleesaatsrddlhilktpresitkieemaftekkqtftasktgmslfgpsqfpteer	3773
Culex quinquefasciatus; EDS38616.1	lgvleetmvtsrddlhvlktpresitkieplse------kieskagmslfgaeqfpadgk	3736
Culex pipiens pallens; XP_039443854.1	lgvleetmvtsrddlhvlktpresitkieplse------kieskagmslfgaeqfpadgk	3873

Anopheles merus; XP_041782981.1	--	2806
Anopheles arabiensis; XP_040172246.1	--	2808
Anopheles coluzzii; XP_040218532.1	--	2808
Anopheles darlingi; ETN65512.1	--	1055
Aedes albopictus; XP_029723621.1	--	1616
Drosophila melanogaster; ABW09325.1	kvtsqftttts---s--------atkddslketvaeflatekivsakeafsteatksadd	4698
Aedes aegypti; XP_021710788.1	keaegfpiagaehespdqtdtiipgvdqllketassmkeiekmsss--------------	3819
Culex quinquefasciatus; EDS38616.1	ketngfpvvglehespdetdsiipgvdvllketvssmkeiekmsts--------------	3782
Culex pipiens pallens; XP_039443854.1	ketngfpvvglehespdetdsiipgvdvllketvssmkeiekmsts--------------	3919

Anopheles merus; XP_041782981.1	--	2806
Anopheles arabiensis; XP_040172246.1	--	2808
Anopheles coluzzii; XP_040218532.1	--	2808
Anopheles darlingi; ETN65512.1	--	1055
Aedes albopictus; XP_029723621.1	--	1616
Drosophila melanogaster; ABW09325.1	clkkttasavsstsasqralfvgtdesrresllsq-asesrlt--hsdpedeepaddvde	4755
Aedes aegypti; XP_021710788.1	--------vi---------tmiqttekkdesikqesk-sstlafplldsgkstpmsge--	3859
Culex quinquefasciatus; EDS38616.1	--------vi---------smiktsekkdetvkieeksastlsfplldsgkstpmstd--	3823
Culex pipiens pallens; XP_039443854.1	--------vi---------smiktsekkdetvkieeksastlsfplldsgkstpmstd--	3960

Anopheles merus; XP_041782981.1	--	2806
Anopheles arabiensis; XP_040172246.1	--	2808
Anopheles coluzzii; XP_040218532.1	--	2808
Anopheles darlingi; ETN65512.1	--	1055
Aedes albopictus; XP_029723621.1	--	1616
Drosophila melanogaster; ABW09325.1	rssvkesrsksiatimmtsiykpsedmepisklveeehehveelaqevtstsktttllqs	4815
Aedes aegypti; XP_021710788.1	-------kdksqt--------------ssikstsphdkdslegss-----a-slntfiek	3892
Culex quinquefasciatus; EDS38616.1	-------kdksqa--------------ssiksvsphekdsldgss-----a-slntfiek	3856
Culex pipiens pallens; XP_039443854.1	-------kdksqa--------------ssiksvsphekdsldgss-----a-slntfiek	3993

Anopheles merus; XP_041782981.1	--	2806
Anopheles arabiensis; XP_040172246.1	--	2808
Anopheles coluzzii; XP_040218532.1	--	2808
Anopheles darlingi; ETN65512.1	--	1055
Aedes albopictus; XP_029723621.1	--	1616
Drosophila melanogaster; ABW09325.1	seqsstttsstsktgasrvesitltqmd--qqtsqsqgdpadrktpptapvspgvkamss	4873
Aedes aegypti; XP_021710788.1	dslse-stskqvtikmsaveeiadlksmttstkvectellggsktpptapispnvakedc	3951
Culex quinquefasciatus; EDS38616.1	dslse-sttkhvtikmstveeigct-tvttgskaegsellggaktpptapispnvvredf	3914
Culex pipiens pallens; XP_039443854.1	dslse-sttkhvtikmstveeigct-tvttgskaegsellggaktpptapispnvvredf	4051

Anopheles merus; XP_041782981.1	--	2806
Anopheles arabiensis; XP_040172246.1	--	2808
Anopheles coluzzii; XP_040218532.1	--	2808
Anopheles darlingi; ETN65512.1	--	1055
Aedes albopictus; XP_029723621.1	--	1616
Drosophila melanogaster; ABW09325.1	tg---------sagsvigagagavaaggkcessaasivsssgpmsp--kdisgksspgal	4922
Aedes aegypti; XP_021710788.1	tlgqhiestvtsisattgtss---sqgivkqq-k---edvsgistpkesvpsgksspglm	4004
Culex quinquefasciatus; EDS38616.1	slghqlessststtttitsssqqqlvggskqq-k---edvsgistpkesvpsgksspglm	3970
Culex pipiens pallens; XP_039443854.1	slghqlessststtttitsssqqqlvggikqq-k---edvsgistpkesvpsgksspglm	4107

Anopheles merus; XP_041782981.1	--	2806
Anopheles arabiensis; XP_040172246.1	--	2808
Anopheles coluzzii; XP_040218532.1	--	2808
Anopheles darlingi; ETN65512.1	--	1055
Aedes albopictus; XP_029723621.1	--	1616
Drosophila melanogaster; ABW09325.1	tse-----sqsiptplgreshtdtpesspkptspfprvskdelkslem-----------q	4966
Aedes aegypti; XP_021710788.1	svhtgstdsasksinlghssgietsesspkptspfpklldtl-ktdevktssgmstpdmt	4063
Culex quinquefasciatus; EDS38616.1	svhtgstdsasksinlghssgietsesspkptspfpkvvdtl-ktdevktssgmstpdmt	4029
Culex pipiens pallens; XP_039443854.1	svhtgstdsasksinlghssgietsesspkptspfpkvvdtl-ktdevktssgmstpdmt	4166

Anopheles merus; XP_041782981.1	--	2806
Anopheles arabiensis; XP_040172246.1	--	2808
Anopheles coluzzii; XP_040218532.1	--	2808
Anopheles darlingi; ETN65512.1	--	1055
Aedes albopictus; XP_029723621.1	--	1616
Drosophila melanogaster; ABW09325.1	hhsqeqmlagaaaaagaecegdipelhelrglecttalsgstdkiitttittvtkvisad	5026
Aedes aegypti; XP_021710788.1	rtstpdmvdsqie--------ripedt----------keevkqdvitttttttkryiikd	4105
Culex quinquefasciatus; EDS38616.1	rtstpdmvdsqie--------rvpedk----------agggesvtttttttttkryivkd	4071
Culex pipiens pallens; XP_039443854.1	rtstpdmvdsqie--------rvpedk----------agggesvtttttttttkryivkd	4208

Anopheles merus; XP_041782981.1	--	2806
Anopheles arabiensis; XP_040172246.1	--	2808
Anopheles coluzzii; XP_040218532.1	--	2808
Anopheles darlingi; ETN65512.1	--	1055
Aedes albopictus; XP_029723621.1	--	1616
Drosophila melanogaster; ABW09325.1	gkeivteqktv--tttd----------------------ssepdsekvv----vttt---	5055
Aedes aegypti; XP_021710788.1	gekievgstvktevsgdskkddtssagesmststttttttkvvkeaiktpinltlatte	4165
Culex quinquefasciatus; EDS38616.1	gekievgstvktevatdtkkddas---esmstssvttittgkptkevvktpgiltlsste	4128
Culex pipiens pallens; XP_039443854.1	gekievgstvktevatdtkkddas---esmstssvttittgkptkevvktpgiltlsste	4265

Anopheles merus; XP_041782981.1	--	2806
Anopheles arabiensis; XP_040172246.1	--	2808
Anopheles coluzzii; XP_040218532.1	--	2808
Anopheles darlingi; ETN65512.1	--	1055
Aedes albopictus; XP_029723621.1	--	1616
Drosophila melanogaster; ABW09325.1	-rtts--eserdqllp-------kevallrglyrastpgse---ddedlllgsprsatsy	5102
Aedes aegypti; XP_021710788.1	drsltedydekdvmsprsdissgqasrivagwhdedvpgspmsvtsqaplspstkytydy	4225
Culex quinquefasciatus; EDS38616.1	drsfaedfdekeamsprsdissgqasrivagwhdedvpgspmsvtsqaplspstkytydy	4188
Culex pipiens pallens; XP_039443854.1	drsfaedfdekeamsprsdissgqasrivagwhdedvpgspmsvtsqaplspstkytydy	4325

Anopheles merus; XP_041782981.1	--	2806
Anopheles arabiensis; XP_040172246.1	--	2808
Anopheles coluzzii; XP_040218532.1	--	2808
Anopheles darlingi; ETN65512.1	--	1055
Aedes albopictus; XP_029723621.1	--	1616
Drosophila melanogaster; ABW09325.1	elqhsssgvskrsdldadgdesqddippqygseehstarsillprtadpmatsfygalpd	5162
Aedes aegypti; XP_021710788.1	dlqhsssgvskksdiei--ddsqdeippqygsdevksaitiasafkpdpmstsfygqlpd	4283
Culex quinquefasciatus; EDS38616.1	dlqhsssgvskksdiei--ddsqdeippqygsdevksaitiasaykpdpmstsfygqlpd	4246
Culex pipiens pallens; XP_039443854.1	dlqhsssgvskksdiei--ddsqdeippqygsdevksaitiasaykpdpmstsfygqlpd	4383

Anopheles merus; XP_041782981.1	--	2806
Anopheles arabiensis; XP_040172246.1	--	2808
Anopheles coluzzii; XP_040218532.1	--	2808
Anopheles darlingi; ETN65512.1	--	1055
Aedes albopictus; XP_029723621.1	--	1616
Drosophila melanogaster; ABW09325.1	sfdvvmkp-----stepipiqga-------------psgdsqssesvesssqtwaghkfl	5204
Aedes aegypti; XP_021710788.1	vsdtavttsvtstksvpipit--tgkaftktymeyassgdssvdsshihk---sadrkyl	4338
Culex quinquefasciatus; EDS38616.1	isdstpv-gitasksvpipisapgkaafaktymeyassgessvdsshqhhktissdrkyl	4305
Culex pipiens pallens; XP_039443854.1	isdstpv-gttasksvpipisapgkaafaktymeyassgessvdsshqhhktissdrkyl	4442

Anopheles merus; XP_041782981.1	--	2806
Anopheles arabiensis; XP_040172246.1	--	2808
Anopheles coluzzii; XP_040218532.1	--	2808
Anopheles darlingi; ETN65512.1	--	1055
Aedes albopictus; XP_029723621.1	--	1616
Drosophila melanogaster; ABW09325.1	dqadkdfqraleehvqargaevmssvtakysyspsk---------aeemeqivsgtaerq	5255
Aedes aegypti; XP_021710788.1	deadldfekaftksdkv---dlmtq---smhfssekefmaattttssamttvadsskkel	4392
Culex quinquefasciatus; EDS38616.1	deadmdfektfak---a---dlmtt---smhfssekefmaat--------tiadsskkel	4348
Culex pipiens pallens; XP_039443854.1	deadmdfektfak---a---dlmtt---smhfssekefmaat--------tiadsskkel	4485

Anopheles merus; XP_041782981.1	--	2806
Anopheles arabiensis; XP_040172246.1	--	2808
Anopheles coluzzii; XP_040218532.1	--	2808
Anopheles darlingi; ETN65512.1	--	1055
Aedes albopictus; XP_029723621.1	--	1616
Drosophila melanogaster; ABW09325.1	rfplsdvqrarvaesgfatvgsvasqqqqqekggeveqavptttavtasttatasstgal	5315
Aedes aegypti; XP_021710788.1	df----------tasglpttisasqaqttlttt-aqss-----ttattsgstsqqsqsdk	4436
Culex quinquefasciatus; EDS38616.1	df----------tasglpstattsttaqsttqq-sssssssttsasttvttsttassstsq	4397
Culex pipiens pallens; XP_039443854.1	df----------tasglpstattsttaqsttqq-sssssssttsasttvt------tstsq	4528

Anopheles merus; XP_041782981.1	--	2806
Anopheles arabiensis; XP_040172246.1	--	2808
Anopheles coluzzii; XP_040218532.1	--	2808
Anopheles darlingi; ETN65512.1	--	1055
Aedes albopictus; XP_029723621.1	--	1616
Drosophila melanogaster; ABW09325.1	pkdrleewgkplglpspaplpveggadirttpkkerrlvatktrlnneknlrrrsespnk	5375
Aedes aegypti; XP_021710788.1	dkdplaswgkplglpspaplnd---enmkttpkrerkmilsktklnneknlrkrsespik	4493
Culex quinquefasciatus; EDS38616.1	pqdplaswgkplglpspaplnd---dn-kttpkkerrtlisktklnneknlrkrsespik	4453
Culex pipiens pallens; XP_039443854.1	pqdplaswgkplglpspaplnd---dn-kttpkkerrtlisktklnneknlrkrsespik	4584

Anopheles merus; XP_041782981.1	--	2806
Anopheles arabiensis; XP_040172246.1	--	2808
Anopheles coluzzii; XP_040218532.1	--	2808
Anopheles darlingi; ETN65512.1	--	1055
Aedes albopictus; XP_029723621.1	--	1616
Drosophila melanogaster; ABW09325.1	agkk--papvyvdltyvphngnsyyahvdffkrvraryyvfsgtepsrqvydalleakqt	5433
Aedes aegypti; XP_021710788.1	sakr--papvyvdlsyvphhgnsyyanveffkrvraryyvfsgtepsrevynalleakqt	4551
Culex quinquefasciatus; EDS38616.1	gakktptapvyvdlsyvphhgnsyyanveffkrvraryyvfsgtepsrevynalleakqt	4513
Culex pipiens pallens; XP_039443854.1	gakktptapvyvdlsyvphhgnsyyanveffkrvraryyvfsgtepsrevynalleakqt	4644

Anopheles merus; XP_041782981.1	--	2806
Anopheles arabiensis; XP_040172246.1	--	2808
Anopheles coluzzii; XP_040218532.1	--	2808
Anopheles darlingi; ETN65512.1	--	1055
Aedes albopictus; XP_029723621.1	--	1616
Drosophila melanogaster; ABW09325.1	wedkelevtiiptydtdvlgywvaeneellakhridlspsasrctinlqdhetscsayrl	5493
Aedes aegypti; XP_021710788.1	wedkelevtiiptydtdvlgywvsenedllakyhidlspsaarctinlqdhetscsayrl	4611
Culex quinquefasciatus; EDS38616.1	wedkdlevtiiptydtdvlgywvsenedllakyhidlspsaarctinlqdhetscsayrl	4573
Culex pipiens pallens; XP_039443854.1	wedkdlevtiiptydtdvlgywvsenedllakyhidlspsaarctinlqdhetscsayrl	4704

Anopheles merus; XP_041782981.1	--	2806
Anopheles arabiensis; XP_040172246.1	--	2808
Anopheles coluzzii; XP_040218532.1	--	2808
Anopheles darlingi; ETN65512.1	--	1055
Aedes albopictus; XP_029723621.1	--	1616
Drosophila melanogaster; ABW09325.1	ef	5495
Aedes aegypti; XP_021710788.1	ef	4613
Culex quinquefasciatus; EDS38616.1	ef	4575
Culex pipiens pallens; XP_039443854.1	ef	4706

**Percent Identity Matrix for futsch**	*Anopheles merus*; XP_041782981.1	*Anopheles arabiensis*; XP_040172246.1	*Anopheles coluzzii*; XP_040218532.1	*Anopheles darlingi*; ETN65512.1	*Aedes albopictus*; XP_029723621.1	*Drosophila melanogaster*; ABW09325.1	*Aedes aegypti*; XP_021710788.1	*Culex quinquefasciatus*; EDS38616.1	*Culex pipiens pallens*; XP_039443854.1
Anopheles merus; XP_041782981.1	100.00	95.04	95.18	16.82	17.21	19.97	19.93	21.07	21.14
Anopheles arabiensis; XP_040172246.1	95.04	100.00	96.76	17.03	16.72	19.36	19.19	20.49	20.59
Anopheles coluzzii; XP_040218532.1	95.18	96.76	100.00	16.77	16.56	19.11	19.25	20.21	20.32
Anopheles darlingi; ETN65512.1	16.82	17.03	16.77	100.00	16.02	19.70	20.41	21.84	22.30
Aedes albopictus; XP_029723621.1	17.21	16.72	16.56	16.02	100.00	20.15	19.78	19.60	19.48
Drosophila melanogaster; ABW09325.1	19.97	19.36	19.11	19.70	20.15	100.00	39.18	40.49	41.10
Aedes aegypti; XP_021710788.1	19.93	19.19	19.25	20.41	19.78	39.18	100.00	69.14	69.23
Culex quinquefasciatus; EDS38616.1	21.07	20.49	20.21	21.84	19.60	40.49	69.14	100.00	98.50
Culex pipiens pallens; XP_039443854.1	21.14	20.59	20.32	22.30	19.48	41.10	69.23	98.50	100.00

S1.4. Multiple sequence alignment for gephyrin (Gene ID: 10243)

Homo sapiens; NP_001364448.1 mategmiltnhdhqirvgvltvsdscfrnlaedrsginlkdlvqdpsllggtisaykivp 60
Drosophila melanogaster; P39205.3 -----------Mesitfgvltisdtcwqepekdtsgpilrqliget--fantqvignivp 47
Culex quinquefasciatus; EDS42142.1 -------------Mmkfsvitvsdscsngtatdtsgphlvslvkek--ldatdvnylvvp 45
Culex pipiens pallens; XP_039440701.1 --------------mqtnditvsdscsngtatdtsgphlvslvkek--ldatdvnylvvp 44
Anopheles arabiensis; XP_040156974.1 ---------------mfsvitvsdscaagkatdtsgphlvelikqs--lktdtvnyllip 43
Anopheles darlingi; ETN60700.1 ---------------mfyvitisdscsageakdtsgpllvgliket--lktesvnyllvp 43
:*:**:* * ** * *: : : ::*

Homo sapiens; NP_001364448.1 deieeiketlidwcdekelnliltggtgfaprdvtpekfptfpfcglqkgatkeviere 120
Drosophila melanogaster; P39205.3 dekdiiqqelrkwidreelrvilttggtgfaprdvtpe-------------atrqlleke 94
Culex quinquefasciatus; EDS42142.1 deadviqklliyqcdvlkvravfttggtgfalrdvtpe-------------atravltke 92
Culex pipiens pallens; XP_039440701.1 deadviqklliyqcdvlkvravfttggtgfaprdvtpe-------------atravltke 91
Anopheles arabiensis; XP_040156974.1 deedlikqsliyacdvlkvravfttggtgfaprdvtpe-------------atraiitke 90
Anopheles darlingi; ETN60700.1 derdlieksllyacdvlkvravfttggtgfaprdvtpe-------------atravitke 90
** : *:: * * ::. ::******** ****** **: :: :*

Homo sapiens; NP_001364448.1 apgmalamlmgslnvtplgmlsrpvcgirgktliinlpgskkgsqecfqfilpalphaid 180
Drosophila melanogaster; P39205.3 cpqlsmyitlesikqtqyaalsrglcgiagntlilnlpgsekavkecfqtisallphavh 154
Culex quinquefasciatus; EDS42142.1 apqltmamtltslektkfavlsravcgvrektlivnfpgskkavdecfhsivnviphilq 152
Culex pipiens pallens; XP_039440701.1 apqltmamtltslektkfavlsravcgvrektlivnfpgskkavdecfhsivnviphilq 151
Anopheles arabiensis; XP_040156974.1 apqltlamtlcslektkfavlsravcgvrnktlvvnfpgskkavgecfqsivdvlphlln 150
Anopheles darlingi; ETN60700.1 apqlslamtlrslektkfavlsravcgirdrtlivnlpgskkavqecyesivdvlphvln 150
.* ::: : : *:: * . *** :**: .**::*:***:*. **:. * :** :.

Homo sapiens; NP_001364448.1	llrd-aivkvkevhdeledlpspppplspppttsphkqtedkgvqceeeeeekkdsgvas	239
Drosophila melanogaster; P39205.3	ligd-dvslvrkthaevqgsa---------------------------------------	174
Culex quinquefasciatus; EDS42142.1	lmad-eipkvkethrkiqsge---------------------------------------	172
Culex pipiens pallens; XP_039440701.1	lmsd-eipkvkethrkiqsge---------------------------------------	171
Anopheles arabiensis; XP_040156974.1	llnegeiervrethrkvqatgdgpvpq---------------------------------	177
Anopheles darlingi; ETN60700.1	llsdgeiesvrethrevqqg---ttnt---------------------------------	174
	*: : : *:::* :::	

Homo sapiens; NP_001364448.1	tedsssshitaaaiaakipdsiisrgvqvlprdtaslsttpsespraqatsrlstascpt	299
Drosophila melanogaster; P39205.3	----qks--hicph	182
Culex quinquefasciatus; EDS42142.1	----ghv--hvcph	180
Culex pipiens pallens; XP_039440701.1	----ghv--hvcph	179
Anopheles arabiensis; XP_040156974.1	----evy--hvcph	185
Anopheles darlingi; ETN60700.1	----krq--hvcph	182
	**	

Homo sapiens; NP_001364448.1	pkvqsrcsskenilrashsavditkvarrhrmspfpltsmdkafitvlemtpvlgteiin	359
Drosophila melanogaster; P39205.3	k---------------------tgtgtdsdrnspypmlpvqevlsiifntvqktan----	217
Culex quinquefasciatus; EDS42142.1	a---------------------tgkggd-drnspypmlevvdaleiimstiatqki----	214
Culex pipiens pallens; XP_039440701.1	a---------------------tgkggd-drnspypmlevvdaleiimstiatqki----	213
Anopheles arabiensis; XP_040156974.1	a---------------------tgkggd-drnspfpmidvddalrkilttvpsvqt----	219
Anopheles darlingi; ETN60700.1	a---------------------tgkgsdddrnspypmisvdealqkilatlpsiet----	217
	. .* **:*: : ..: ::	

Homo sapiens; NP_001364448.1	yrdgmgrvlaqdvyakdnlppfpasvkdgyavraadgpgdrfiigesqageqptqtvm-p	418
Drosophila melanogaster; P39205.3	-----lnkillemnapvnippfrasikdgyamkstgfsgtkrvlgciaagdspnslplae	272
Culex quinquefasciatus; EDS42142.1	-----pr----kqisrvnippfrasvkdgyalkssgdkgvkrvvgyvaagngiirdyfdi	265
Culex pipiens pallens; XP_039440701.1	-----pr----kqisrvnippfrasvkdgyalkssgdkgvkrvvgyvaagngiirdyfdi	264
Anopheles arabiensis; XP_040156974.1	-----ar----kqlsrvnippfrasikdgyalksiggkgmkkvigyisagdaivqtnfti	270
Anopheles darlingi; ETN60700.1	-----tr----pqmsrvnippfrasikdgyalksiggkgmkkvigyiaagdaivrtnfti	268
	. : *:*** **:*****:::. * : ::* **: :	

Homo sapiens; NP_001364448.1	gqvmrvttgapipcgadavvqvedteliresddgteelevrilvqarpgqdirpighdik	478
Drosophila melanogaster; P39205.3	decykintgaplpleadcvvqvedtkllqldkngqes-lvdilvepqagldvrpvgydls	331
Culex quinquefasciatus; EDS42142.1	dqcykintgapvpdqadaviqiedtklist-dndiek-iievlsspspnldirsigsdlr	323
Culex pipiens pallens; XP_039440701.1	dqcykintgapvpdqadaviqiedtklist-dndiek-iievlsspspnldirsigsdlr	322
Anopheles arabiensis; XP_040156974.1	decykintgapvpqhadaiiqvedtklvsr-kndyes-iveilaepsasldirsigsdlr	328
Anopheles darlingi; ETN60700.1	decykintgaplpehadaiiqiedtklisr-endyer-mievltdpepsldvraigsdlr	326
	.: ::.****:* **.::*:***:*: .:. * : :* . .*:* :* *:	

Homo sapiens; NP_001364448.1	rgecvlakgthmgpseigllatvgvtevevnkfpvvavmstgnellnpedd-------ll	531
Drosophila melanogaster; P39205.3	tndrifpaldpspvvvksllasvgnrlil--skpkvaivstgselcsprnq-------lt	382
Culex quinquefasciatus; EDS42142.1	mgedvfehrypldaaqrsllasigetinv--gqlriavistgdelvhpygdgaq--trti	379
Culex pipiens pallens; XP_039440701.1	mgedvfehrypldaaqrsllasigetinv--gqlriavistgdelvhpygdgaq--tgti	378
Anopheles arabiensis; XP_040156974.1	mseevfqyrfpldacqrallaavgekasv--vklkvaiistgdellhpydtsaaadassl	386
Anopheles darlingi; ETN60700.1	maeevfryrfpldasqrgmlasigekvsl--vklkvailstgdellhpydaipa--egsl	382
	: :: .:**::* :*::***.** * .	

Homo sapiens; NP_001364448.1	pgkirdsnrstllatiqehgyptin----lgivgdnpddllnalnegisradviitsggv	587
Drosophila melanogaster; P39205.3	pgkifdsnttmltellvyfgfncmh----tcvlsdsfqrtkesllelfevvdfvicsggv	438
Culex quinquefasciatus; EDS42142.1	egkifdsnttmlvqmlrqfgfteeqclvsqivvhddyqslrkqieiltgsvhailctggv	439
Culex pipiens pallens; XP_039440701.1	egkifdsnttmlvqllrqfgfteeqclvsqivvhddyqslrkqieiltgsvhailctggv	438
Anopheles arabiensis; XP_040156974.1	egkifdsnttmlvalvrqcgftedqceiqqrvvkddfeslkaeiesltgavhiiictggv	446
Anopheles darlingi; ETN60700.1	egkiydsnttmlvalirqfgftedqceirqqvvkddfeslenavasltgsmhviictggv	442
	*** *** : * : *: : :: *. : : . :: :***	

Homo sapiens; NP_001364448.1	smgekdylkqvldidlhaqihfgrvfmkpglpttfatldidgvrkiifalpgnpvsavvt	647
Drosophila melanogaster; P39205.3	smgdkdfvksvle-dlqfrihcgrvnikpgkpmtfasrkd----kyffglpgnpvsafvt	493
Culex quinquefasciatus; EDS42142.1	smgdkdfvkpvlk-dlgydlkfgrvnmkpgkpftfaskdi----trffglpgnpvsafvt	494
Culex pipiens pallens; XP_039440701.1	smgdkdfvkpvlk-dlgydlkfgrvnvkpgkpftfaskdi----trffglpgnpvsafvt	493
Anopheles arabiensis; XP_040156974.1	smgdkdfvkpvlk-alnyelifgrvnmkpgkpcayatskv----tkffglpgnpvsafvt	501
Anopheles darlingi; ETN60700.1	smgdkdfvkpvlk-hlgyklvfgrvnvkpgkpctyasspt----tkffglpgnpvsafvt	497
	:**::* **. * : *** : * ::*: . :*.********.**	

Homo sapiens; NP_001364448.1	cnlfvvpalrkmqgildpr---------ptiikarlscdvkldprpeyhrciltwhhqep	698
Drosophila melanogaster; P39205.3	fhlfalpairfaag------wdrckcslsvlnvklln-dfsldsrpefvrasvisk--sg	544
Culex quinquefasciatus; EDS42142.1	fhlfalpalrkylatlnqttsnlakchlpkitvklltesyeldprpeyarasifsk--gg	552
Culex pipiens pallens; XP_039440701.1	fhlfalpalrkylatlnqttsnlakchlpkitvklltesyeldprpeyarasivsk--gg	551
Anopheles arabiensis; XP_040156974.1	fhlfalpalrqylatlnetapntakaslpmitvelldakyvldprpeyarasivsr--ng	559
Anopheles darlingi; ETN60700.1	fhlfalpalrwylcainetkpqiakcclpmidvelldsineldprpefaratirsr--kg	555
	:**.:**:* : * ** ***: *. : :	

Homo sapiens; NP_001364448.1	lpwaqstgnqmssrlmsmrsangllmlppkteqyvelhkgevvdvmvigrl------	749
Drosophila melanogaster; P39205.3	elyasvngnqissrlqsivgadvlinlpartsdrplakageifpasvlrfdfiskye	601
Culex quinquefasciatus; EDS42142.1	elwaqitgqqissrlksiveadvllhlparspskttivrgesvtaltlradfistye	609
Culex pipiens pallens; XP_039440701.1	elwaqitgqqissrlksiveadvllhlpvrspskttivrgesvtaltlradfisaye	608
Anopheles arabiensis; XP_040156974.1	kllasitggqissrlkstieadvllelpartetkpyitagtplkalvlrsdfisrye	616
Anopheles darlingi; ETN60700.1	kllasvtgnqlssrlkstidadalielpaktgertslkagtlvkayvirsdfvsyyd	612
	. . *:**** * *: *: ** :: * . . .:	

**Percent Identity Matrix for gephyrin**	*Homo sapiens; NP_001364448.1*	*Drosophila melanogaster; P39205.3*	*Culex quinquefasciatus; EDS42142.1*	*Culex pipiens pallens; XP_039440701.1*	*Anopheles arabiensis; XP_040156974.1*	*Anopheles darlingi; ETN60700.1*
Homo sapiens; NP_001364448.1	100.00	38.91	40.75	40.65	41.16	41.47
Drosophila melanogaster; P39205.3	38.91	100.00	50.08	50.00	51.10	51.44
Culex quinquefasciatus; EDS42142.1	40.75	50.08	100.00	98.03	69.19	65.84
Culex pipiens pallens; XP_039440701.1	40.65	50.00	98.03	100.00	68.86	66.17
Anopheles arabiensis; XP_040156974.1	41.16	51.10	69.19	68.86	100.00	76.10
Anopheles darlingi; ETN60700.1	41.47	51.44	65.84	66.17	76.10	100.00

S1.5. Multiple sequence alignment for phosphosynapsin (Gene ID: 6853)

Homo sapiens; NP_008881.2	-------------mnylrrrlsdsnfmanlpngymtdl--------qrp-----------	28
Drosophila melanogaster; AAF54506.3	mnfssfkssftsnvnflkrrfssgdlssevddvdpnslppaarpiqdqptkppvaggppn	60
Anopheles darlingi; ETN61740.1	--------mfr---trthenrrsgdlssecedvdpkdlppaarsqqpplqqqpmpggpps	49
Anopheles stephensi; XP_035891026.1	---mpggppn	7
Anopheles arabiensis; XP_040154215.1	---mpggppn	7
Anopheles merus; XP_041764250.1	---mpggppn	7
Anopheles coluzzii; XP_040240634.1	---mpggppn	7
Culex quinquefasciatus; XP_038122578.1	---mpggppn	7
Aedes aegypti; XP_021705226.1	---mpggppn	7
Aedes albopictus; XP_029728640.1	---mpggppn	7

Homo sapiens; NP_008881.2	qppppppgahspgatpgp---------gtataerssgvapaaspaap--spgssggg---	74
Drosophila melanogaster; AAF54506.3	mppppapgqpag---------------aapelslsfgagktpataapapprgvsaptspa	105
Anopheles darlingi; ETN61740.1	ippppapgt-gavspggpvppgsapptggpelslsfgkgp---------qrgtsapsspa	99
Anopheles stephensi; XP_035891026.1	ippppapgtagsvspggpvppgsapptggpelslsfgkgp--------aqrgtsapsspa	59
Anopheles arabiensis; XP_040154215.1	ippppapgtagsvspggpvppgsapptggpelslsfgkgp--------aqrgtsapsspa	59
Anopheles merus; XP_041764250.1	ippppapgtagsvspggpvppgsapptggpelslsfgkgp--------aqrgtsapsspa	59
Anopheles coluzzii; XP_040240634.1	ippppapgtagsvspggpvppgsapptggpelslsfgkgp--------aqrgtsapsspa	59
Culex quinquefasciatus; XP_038122578.1	ippppapgtagsavspgpv-pgsapptggpelslsfgkgp---------qrgtsapsspa	57
Aedes aegypti; XP_021705226.1	ippppapg---savspgpv-pgsapptggpelslsfgkgp---------qrgtsapsspa	54
Aedes albopictus; XP_029728640.1	ippppapg---savspgpv-pgsapptggpelslsfgkgp---------qrgtsapsspa	54
	**** ** . .* * . * *.	

Homo sapiens; NP_008881.2	gffsslsnavkqttaaaaatfseqvgggsgg-----agrggaasrvllvidephtdwaky	129
Drosophila melanogaster; AAF54506.3	ksresllqrvqsltgaardqgasilgaavqsatqrapafskdkyftllvlddqntdwsky	165
Anopheles darlingi; ETN61740.1	ksresllqrvqsltgaardqgasiigaavstas-rvqpfnrdkcftllvvddqntdwsky	158
Anopheles stephensi; XP_035891026.1	ksresllqrvqsltgaardqgasiigaavstas-rvqpfnrdkcftllvvddqntdwsky	118
Anopheles arabiensis; XP_040154215.1	ksresllqrvqsltgaardqgasiigaavstas-rvqpfnrdkcftllvvddqntdwsky	118
Anopheles merus; XP_041764250.1	ksresllqrvqsltgaardqgasiigaavstas-rvqpfnrdkcftllvvddqntdwsky	118
Anopheles coluzzii; XP_040240634.1	ksresllqrvqsltgaardqgasiigaavstas-rvqpfnrdkcftllvvddqntdwsky	118
Culex quinquefasciatus; XP_038122578.1	ksresllqrvqsltgaardqgasiigaavstaa-rvqpfnkdkcftllvlddqntdwsky	116
Aedes aegypti; XP_021705226.1	ksresllqrvqsltgaardqgasiigaavssaa-rvqpfnkdkcftllvlddqntdwsky	113
Aedes albopictus; XP_029728640.1	ksresllqrvqsltgaardqgasiigaavssaa-rvqpfnkdkcftllvlddqntdwsky	113
	.** : *:.. *.** :. :*.. . .***:*: :***:**	

Homo sapiens; NP_008881.2	fkgkkihgeidikveqaefsdlnlvahanggfsvdmevlrngvkvvrslkpdfvlirqha	189
Drosophila melanogaster; AAF54506.3	frgrrlhgdfdirveqaefrditvvssadtgpvvtmaayrsgtrvarsfrpdfvlirqpp	225
Anopheles darlingi; ETN61740.1	frgkrlhsdydirveqaefreitltasadsgpmvgfnr-gggskqakpfrpdfilvrqpp	217
Anopheles stephensi; XP_035891026.1	frgkrlhsdydirveqaefreimltasadsgpmvgcnr-g-gskqakpfrpdfilvrqpp	176
Anopheles arabiensis; XP_040154215.1	frgkrlhsdydirveqaefreimltasadsgpmvgcnr-a-gskqvkpfrpdfilvrqpp	176
Anopheles merus; XP_041764250.1	frgkrlhsdydirveqaefreimltasadsgpmvgcnr-a-gskqakpfrpdfilvrqpp	176
Anopheles coluzzii; XP_040240634.1	frgkrlhsdydirveqaefreimltasadsgpmvgcnr-g-gskqakpfrpdfilvrqpp	176
Culex quinquefasciatus; XP_038122578.1	frgkrlhgdydirveqaefreisltanadagplvsfnr-g-sskqpksfrpdfilvrqap	174
Aedes aegypti; XP_021705226.1	frgkrlhgdydirveqaefreisltanadagplvsfnr-g-sskqpksyrpdfilvrqap	171
Aedes albopictus; XP_029728640.1	frgkrlhgdydirveqaefreisltanadagplvsfnr-g-sskqpksyrpdfilvrqap	171
	::::*.: **:****** :: :.: *: * * .: : :***:*:**	

Homo sapiens; NP_008881.2	fsmarngdyrslviglqyagipsvnslhsvynfcdkpwvfaqmvrlhkklgteefplidq	249
Drosophila melanogaster; AAF54506.3	r--dgssdyrstilglkyggvpsinslhsiyqfqdkpwvfshllqlqrrlgrdgfplieq	283
Anopheles darlingi; ETN61740.1	r--dgskdyrstllglkyggvpsinslhslyqfqdkpwvfahllqlqrrlgrdvfplveq	275
Anopheles stephensi; XP_035891026.1	r--dgskdyrstllglkyggvpsinslhslyqfqdkpwvfahllqlqrrlgrdafplveq	234
Anopheles arabiensis; XP_040154215.1	r--dgskdyrstllglkyggvpsinslhslyqfqdkpwvfahllqlqrrlgrdafplveq	234
Anopheles merus; XP_041764250.1	r--dgskdyrstllglkyggvpsinslhslyqfqdkpwvfahllqlqrrlgrdafplveq	234
Anopheles coluzzii; XP_040240634.1	r--dgskdyrstllglkyggvpsinslhslyqfqdkpwvfahllqlqrrlgrdafplveq	234
Culex quinquefasciatus; XP_038122578.1	r--dgakdyrstllglkyggvpsinslhslyqfqdkpwvfahllqlqrrlgreafplveq	232
Aedes aegypti; XP_021705226.1	r--dgakdyrstllglkyggvpsinslhslyqfqdkpwvfahllqlqrrlgkeafplveq	229
Aedes albopictus; XP_029728640.1	r--dgakdyrstllglkyggvpsinslhslyqfqdkpwvfahllqlqrrlgkegfplveq	229
	**** ::**:* *:**:*****:*:* ******:::::*:::** : ***::*	

Homo sapiens; NP_008881.2	tfypnhkemlssttyp-------vvvkmghahsgmgkvkvdnqhdfqdiasvva-----l	297
Drosophila melanogaster; AAF54506.3	tffpnprdlfqftkfp-------svlkaghchggvatarlenqsalqdaaglvsgagn-d	335
Anopheles darlingi; ETN61740.1	tffpnpkdmtasfytnrqkpsstspvksqdcqm---------------------------	308
Anopheles stephensi; XP_035891026.1	tffpnpkdmftwtrfp-------cvlkaghchggkatakldnpgalqdaagllcgtglsd	287
Anopheles arabiensis; XP_040154215.1	tffpnpkdmftwtrfp-------cvlkaghchggkatakldnpgalqdaagllcgtglsd	287
Anopheles merus; XP_041764250.1	tffpnpkdmftwtrfp-------cvlkaghchggkatakldnpgalqdaagllcgtglsd	287
Anopheles coluzzii; XP_040240634.1	tffpnpkdmftwtrfp-------cvlkaghchggkatakldnpgalqdaagllcgtglsd	287
Culex quinquefasciatus; XP_038122578.1	tffpnpremlawqrfp-------cvlkaghchggkatakldspgalqdaagllcgtglse	285
Aedes aegypti; XP_021705226.1	tffpnprdmlawqrfp-------cvlkaghcyggkatakldnpgalqdaagllcgtglsd	282
Aedes albopictus; XP_029728640.1	tffpnprdmlawqrfp-------cvlkaghchggkatakldnpgalqdaagllcgtglsd	282
	**:** ::: :* ..	

Homo sapiens; NP_008881.2	tktyataepfidakydvrvqkigqnykaymrtsvsgnwktntgsamleqiamsdryklwv	357
Drosophila melanogaster; AAF54506.3	shcyctiepyidakfsvhiqkignnykafmrksitgnwktnqgsamleqitltekykswv	395
Anopheles darlingi; ETN61740.1	--308	
Anopheles stephensi; XP_035891026.1	ngsycslepyidakfdvhiqkigstykafmrksisgnwktnqgsamleqipmtekyktwv	347
Anopheles arabiensis; XP_040154215.1	ngsycslepyidakfdvhiqkigtsykafmrksisgnwktnqgsamleqipmtekyktwv	347
Anopheles merus; XP_041764250.1	ngsycslepyidakfdvhiqkigtsykafmrksisgnwktnqgsamleqipmtekyktwv	347
Anopheles coluzzii; XP_040240634.1	ngsycslepyidakfdvhiqkigtsykafmrksisgnwktnqgsamleqipmtekyktwv	347
Culex quinquefasciatus; XP_038122578.1	stsycslepyidakfdvhiqkiggnykafmrksisgnwktnqgsamleqipmtekykswi	345
Aedes aegypti; XP_021705226.1	stsycslepyvdakfdihiqkigtnykafmrksisgnwktnqgsamleqipmtekykawi	342
Aedes albopictus; XP_029728640.1	stsycslepyvdakfdihiqkigtnykafmrksisgnwktnqgsamleqipmtekykawi	342

Homo sapiens; NP_008881.2	dtcseifggldicavealhgkdgrdhiievvgssmpligdhqdedkqlivelvvnkmaqa	417
Drosophila melanogaster; AAF54506.3	deiselfggmevcglsvvvakdgreyiisacdstfaligdtqeedrrqiadlvsgrmqnv	455
Anopheles darlingi; ETN61740.1	--308	
Anopheles stephensi; XP_035891026.1	devselfggmevcgvavivskegkefiisaadstfplmgdsqeedrrqiadlvvgrmqnv	407
Anopheles arabiensis; XP_040154215.1	devselfggmevcgvavivskegkefiisaadstfplmgdtqeedrrqiadlvvgrmqnv	407
Anopheles merus; XP_041764250.1	devselfggmevcgvavivskegkefiisaadstfplmgdtqeedrrqiadlvvgrmqnv	407
Anopheles coluzzii; XP_040240634.1	devselfggmevcgvavivskegkefiisaadstfplmgdtqeedrrqiadlvvgrmqnv	407
Culex quinquefasciatus; XP_038122578.1	devselfgsmevcgiavivskegkefiisasdstfplmgdtqeedrrqiadlvvgrmqnv	405
Aedes aegypti; XP_021705226.1	devselfggmevcgiavivskegkefiisasdstfplmgdtqeedrrhiadlvvgrmqnv	402
Aedes albopictus; XP_029728640.1	devselfggmevcgiavivskegkefiisasdstfplmgdtqeedrrhiadlvvgrmqnv	402

Homo sapiens; NP_008881.2	lprqrqrdaspgrg-shgqtpspgalplgrqtsqqpagppaqqrpppqggppqpgpgpqr	476
Drosophila melanogaster; AAF54506.3	crpsmaq-tgpgklpsrssvssraesptdegvaptp-plpagprpapmg-gpppipe--r	510
Anopheles darlingi; ETN61740.1	--	308
Anopheles stephensi; XP_035891026.1	crpsmmt-ka----vsrssissrgtspted----ap-pvpigtrpvpvgggpppipe--r	455
Anopheles arabiensis; XP_040154215.1	crpsmmt-ka----vsrssissrgtspted----ap-pvpigtrpvpvgggpppipe--r	455
Anopheles merus; XP_041764250.1	crpsmmt-ka----vsrssissrgtspted----ap-pvpigtrpvpvgggpppipe--r	455
Anopheles coluzzii; XP_040240634.1	crpsmmt-ka----vsrssissrgtspted----ap-pvpigtrpvpvgggpppipe--r	455
Culex quinquefasciatus; XP_038122578.1	crpsmlt-ka----tsrtsissrggsptee----np-pvpigsrpapvgggpppipe--r	453
Aedes aegypti; XP_021705226.1	crpsmmt-ka----tsrssissrggsptee----mp-pvpigtrpapvgggpppipe--r	450
Aedes albopictus; XP_029728640.1	crpsmmt-ka----tsrtsissrggsptee----mp-pvpigtrpapvgggpppipe--r	450

Homo sapiens; NP_008881.2	qgpplqqrpppqgqqhlsglgppagsplpqrlpsptsapqqpasqaapptqgqgrq----	532
Drosophila melanogaster; AAF54506.3	tspavgsi------grlssrssise-----------vpeepss-sgpstvggvrrdsqt	551
Anopheles darlingi; ETN61740.1	--	308
Anopheles stephensi; XP_035891026.1	ttpgvgsi------grhgsfssqsg-----------eppeqpse-raptlnsvgrrdsqa	497
Anopheles arabiensis; XP_040154215.1	ttpgvgsi------grhgsfssqsg-----------eppeqpse-raptlnsvgrrdsqa	497
Anopheles merus; XP_041764250.1	ttpgvgsi------grhgsfssqsg-----------eppeqpse-raptlnsvgrrdsqa	497
Anopheles coluzzii; XP_040240634.1	ttpgvgsi------grhgsfssqsg-----------eppeqpse-raptlnsvgrrdsqa	497
Culex quinquefasciatus; XP_038122578.1	ttpgvgsi------grhgsissqsg-----------eppeqpmd-kaptlnsvgrrdsqa	495
Aedes aegypti; XP_021705226.1	ttpgvgsi------grhgsissqsg-----------eipdqpte-kaptlnslgrrdsqa	492
Aedes albopictus; XP_029728640.1	ttpgvgsi------grhgsissqsg-----------eipeqpse-kaptlnslgrrdsqa	492

Homo sapiens; NP_008881.2	--srpvaggp---------gappaarpp-aspspqrqagppq-atrqt--svsgp-----	572
Drosophila melanogaster; AAF54506.3	sqsstisssvsrag----------qrppqtqnsvvedaedtmknlrktfagifgdmxeia	601
Anopheles darlingi; ETN61740.1	--	308
Anopheles stephensi; XP_035891026.1	sqsssvsgissasaarsaagkappan-atgapsvvedaedtmknlrktfagifgdm----	552
Anopheles arabiensis; XP_040154215.1	sqsssvsgvssasaarsgvgkvpaagagpggpsvvedaedtmknlrktfagifgdm----	553
Anopheles merus; XP_041764250.1	sqsssvsgvssasaarsgvgkvpaagagpggpsvvedaedtmknlrktfagifgdm----	553
Anopheles coluzzii; XP_040240634.1	sqsssvsgvssasaarsgvgkvpaagagpggpsvvedaedtmknlrktfagifgdm----	553
Culex quinquefasciatus; XP_038122578.1	sqsssisgvssasaarsnaskvgp-gpgqgalgnvdeaedtmknlrktfagifgdm----	550
Aedes aegypti; XP_021705226.1	sqsssvsgvssasaarsgvskvtt-lpgqgtv--adeaedtmknlrktfagifgdm----	545
Aedes albopictus; XP_029728640.1	sqsssvsgvssasaarsgvskvtt-vpgqgtv--adeaedtmknlrktfagifgdm----	545

Homo sapiens; NP_008881.2	-------appkasgappggqqrqgppqkppgpagptrq---------asqag--------	608
Drosophila melanogaster; AAF54506.3	nkkrgrtasetssgsgpgs---------vpssagpgsgfsssflgkqfsfagkgegvist	652
Anopheles darlingi; ETN61740.1	--	308
Anopheles stephensi; XP_035891026.1	--	552
Anopheles arabiensis; XP_040154215.1	--	553
Anopheles merus; XP_041764250.1	--	553
Anopheles coluzzii; XP_040240634.1	--	553
Culex quinquefasciatus; XP_038122578.1	--	550
Aedes aegypti; XP_021705226.1	--	545
Aedes albopictus; XP_029728640.1	--	545

Homo sapiens; NP_008881.2	---pvprtgppttqqprpsgpgpagrpkpqlaqkpsqdvppp-ataaaggpphpqlnks-	663
Drosophila melanogaster; AAF54506.3	qptqrpseeppai----pttassavrpessvsvsdsrntdtlteragagyqpvtnyeqqe	708
Anopheles darlingi; ETN61740.1	--	308
Anopheles stephensi; XP_035891026.1	--	552
Anopheles arabiensis; XP_040154215.1	--	553
Anopheles merus; XP_041764250.1	--	553
Anopheles coluzzii; XP_040240634.1	--	553
Culex quinquefasciatus; XP_038122578.1	--	550
Aedes aegypti; XP_021705226.1	--	545
Aedes albopictus; XP_029728640.1	--	545

Homo sapiens; NP_008881.2	---------------------------qsl-------tnafnlpe-pa--pprp--sls	683
Drosophila melanogaster; AAF54506.3	rvnpfdkepsksgsaasihtsssssissssissrinrngnaiqsppppagpppppptnvt	768
Anopheles darlingi; ETN61740.1	--	308
Anopheles stephensi; XP_035891026.1	--	552
Anopheles arabiensis; XP_040154215.1	--	553
Anopheles merus; XP_041764250.1	--	553
Anopheles coluzzii; XP_040240634.1	--	553
Culex quinquefasciatus; XP_038122578.1	--	550
Aedes aegypti; XP_021705226.1	--	545
Aedes albopictus; XP_029728640.1	--	545

Homo sapiens; NP_008881.2	qdevkaetirslrksfaslfsd--------------------------------------	705
Drosophila melanogaster; AAF54506.3	avgsnansssgyrnsfssslskdktsygnygsttsvetitrmdtnttnigatateageas	828
Anopheles darlingi; ETN61740.1	--	308
Anopheles stephensi; XP_035891026.1	--	552
Anopheles arabiensis; XP_040154215.1	--	553
Anopheles merus; XP_041764250.1	--	553
Anopheles coluzzii; XP_040240634.1	--	553
Culex quinquefasciatus; XP_038122578.1	--	550
Aedes aegypti; XP_021705226.1	--	545
Aedes albopictus; XP_029728640.1	--	545

Homo sapiens; NP_008881.2	--	705
Drosophila melanogaster; AAF54506.3	gvtaitnisnsdgivapttgtittsvttndwrsaigmrsasvysapaavttvlpgdtsgy	888
Anopheles darlingi; ETN61740.1	--	308
Anopheles stephensi; XP_035891026.1	--	552
Anopheles arabiensis; XP_040154215.1	--	553
Anopheles merus; XP_041764250.1	--	553
Anopheles coluzzii; XP_040240634.1	--	553
Culex quinquefasciatus; XP_038122578.1	--	550
Aedes aegypti; XP_021705226.1	--	545
Aedes albopictus; XP_029728640.1	--	545

Homo sapiens; NP_008881.2	--	705
Drosophila melanogaster; AAF54506.3	dsnsiasqgeglnnpsdlpsytrpsysrsesnaskhsdldvifgdskttpasygngkytr	948
Anopheles darlingi; ETN61740.1	--	308
Anopheles stephensi; XP_035891026.1	--	552
Anopheles arabiensis; XP_040154215.1	--	553
Anopheles merus; XP_041764250.1	--	553
Anopheles coluzzii; XP_040240634.1	--	553
Culex quinquefasciatus; XP_038122578.1	--	550
Aedes aegypti; XP_021705226.1	--	545
Aedes albopictus; XP_029728640.1	--	545

Homo sapiens; NP_008881.2	--	705
Drosophila melanogaster; AAF54506.3	aagsisdadmifggppsnyktdrfgasksmsmtsggvgsgngsgsglggykiydsiqnaa	1008
Anopheles darlingi; ETN61740.1	--	308
Anopheles stephensi; XP_035891026.1	--	552
Anopheles arabiensis; XP_040154215.1	--	553
Anopheles merus; XP_041764250.1	--	553
Anopheles coluzzii; XP_040240634.1	--	553
Culex quinquefasciatus; XP_038122578.1	--	550
Aedes aegypti; XP_021705226.1	--	545
Aedes albopictus; XP_029728640.1	--	545

Homo sapiens; NP_008881.2	---------------------------------	705
Drosophila melanogaster; AAF54506.3	fsdfsdsgsmssigshtkrwsaskeeddeldlk	1041
Anopheles darlingi; ETN61740.1	---------------------------------	308
Anopheles stephensi; XP_035891026.1	---------------------------------	552
Anopheles arabiensis; XP_040154215.1	---------------------------------	553
Anopheles merus; XP_041764250.1	---------------------------------	553
Anopheles coluzzii; XP_040240634.1	---------------------------------	553
Culex quinquefasciatus; XP_038122578.1	---------------------------------	550
Aedes aegypti; XP_021705226.1	---------------------------------	545
Aedes albopictus; XP_029728640.1	---------------------------------	545

**Percent Identity Matrix for phos-phosynapsin**	*Homo sapiens*; NP_008881.2	*Drosophila melanogaster*; AAF54506.3	*Anopheles_darlingi*; ETN61740.1	*Anopheles stephensi*; XP_035891026.1	*Anopheles arabiensis*; XP_040154215.1	*Anopheles merus*; XP_041764250.1	*Anopheles coluzzii*; XP_040240634.1	*Culex quinquefasciatus*; XP_038122578.1	*Aedes aegypti*; XP_021705226.1	*Aedes albopictus*; XP_029728640.1
Homo sapiens; NP_008881.2	100.00	38.63	36.88	41.04	41.35	41.15	41.15	40.04	40.24	40.44
Drosophila melanogaster; AAF54506.3	38.63	100.00	68.18	72.52	72.38	72.57	72.57	71.70	71.62	72.20
Anopheles_darlingi; ETN61740.1	36.88	68.18	100.00	92.25	91.47	91.86	92.25	85.21	84.31	84.31
Anopheles stephensi; XP_035891026.1	41.04	72.52	92.25	100.00	97.46	97.64	97.83	88.52	87.87	88.05
Anopheles arabiensis; XP_040154215.1	41.35	72.38	91.47	97.46	100.00	99.82	99.64	88.55	88.81	88.99
Anopheles merus; XP_041764250.1	41.15	72.57	91.86	97.64	99.82	100.00	99.82	88.55	88.81	88.99
Anopheles coluzzii; XP_040240634.1	41.15	72.57	92.25	97.83	99.64	99.82	100.00	88.73	88.99	89.17
Culex quinquefasciatus; XP_038122578.1	40.04	71.70	85.21	88.52	88.55	88.55	88.73	100.00	94.31	94.68
Aedes aegypti; XP_021705226.1	40.24	71.62	84.31	87.87	88.81	88.81	88.99	94.31	100.00	98.90
Aedes albopictus; XP_029728640.1	40.44	72.20	84.31	88.05	88.99	88.99	89.17	94.68	98.90	100.00

S1.6 Multiple sequence alignment for choline acetyltransferase (Gene ID: 42249)

```
Drosophila melanogaster; AAA28406.2    masneastsaagsgpesaalfsklrsfsigsgpnspqrvvsnlrgflthrlsnitpsdtg   60
Culex quinquefasciatus; EDS31208.1     ------------------------------------------------------------   0
Aedes aegypti; XP_021713002.1          ------------------------------------------------------------   0


Drosophila melanogaster; AAA28406.2    wkdsilsipkkwlstaesvdefgfpdtlpkvpvpaldetmadyiralepittpaqlertk   120
Culex quinquefasciatus; EDS31208.1     -----------------mingft-lrmlpkvpvptldqtmaeylrvlspivtaqqldhtr   42
Aedes aegypti; XP_021713002.1          -------------------------mlpkvpvptldqtmteyqralqpiltpqqlerar   34
                                                                *******:**:**::* *.*.** *    **::::

Drosophila melanogaster; AAA28406.2    elirqfsapqgigarlhqylldkreaednwayyywlnemymdiriplpinsnpgmvfppr   180
Culex quinquefasciatus; EDS31208.1     svikhftapngpgtalqqylldkrdaddnwayyywlndmyldnplplpinsnpgmvmppr   102
Aedes aegypti; XP_021713002.1          giirqftapnglgttlqqflldkreaednwayyywlndmymdnplplpinsnpgmvlppr   94
                                         :*::*:**:* *: *:*:*****:*:***********:**:*    :***********:***

Drosophila melanogaster; AAA28406.2    rfktvhdvahfaarlldgilshremldsgelpleraasreknqplcmaqyyrllgscrrp   240
Culex quinquefasciatus; EDS31208.1     kfttvndisrfgarlidhlmlhkemldggglvqeratsrekgqplcmaqyyrllgscrrp   162
Aedes aegypti; XP_021713002.1          kfttvndlarfaarlvdhlvshkemldsgglaqeratsrekgqplcmaqyyrllgscrrp   154
                                         :*.**:*:::*.***:* :: *:****.* *    ***:****.*******************

Drosophila melanogaster; AAA28406.2    gvkqdsqflpsrerlndedrhvvvicrnqmycvvlqasdrgklseseiasqilyvlsdap   300
Culex quinquefasciatus; EDS31208.1     gdprdsqylpeqr----tdehvvvccrnqmyclpvkagdrgrlnedeiasqllyilndap   218
Aedes aegypti; XP_021713002.1          genrdsqylpeqr----tdehvivccrnqmyclpvkagdrgrlnedeiasqvlhvlndap   210
                                         *    :***:** :..         *.**:* *******: ::*.***:*.*.*****:*::*.***
```

Drosophila melanogaster; AAA28406.2	clpakpvpvglltaeprstwardremlqedernqrnlelietaqvvlcldeplagnfnar	360
Culex quinquefasciatus; EDS31208.1	clarkpprigvlttaqrpqwardrqmllleeqnarnielieqalvlicidepipltynar	278
Aedes aegypti; XP_021713002.1	clsrrasriglltaeprqrwakdrklllleeqnarnielieqalvlicidepipltfnar	270
	** : :*:**: * **:**::* :*:* **:**** * *::*:***: ::***	
Drosophila melanogaster; AAA28406.2	gftgatptvhragdrdetnmahemihgggseynsgnrwfdktmqliictdgtwglcyehs	420
Culex quinquefasciatus; EDS31208.1	gfngspagahycggrdesnmaqemihgggsefnsanrwfdktmqliicndgtwglcyehs	338
Aedes aegypti; XP_021713002.1	gfngspagahycggrdesnmaqemihgggseynsgnrwfdktmqliicndgtwglcyehs	330
	**.*: .* .*.***:***:*********:**.*************.***********	
Drosophila melanogaster; AAA28406.2	csegiavvqllekiykkieehpded-nglpqhhlppperlewhvgpqlqlrfaqasksvd	479
Culex quinquefasciatus; EDS31208.1	psegiavvqllegilkkidempaeagndtaqnhlppperlewivrpeidlrvreaarsid	398
Aedes aegypti; XP_021713002.1	psegiavvqllegiykkidempakee-tgsqdhlppperlewivrpeidqriqeaaknvd	389
	*********** * ***:* * : *.********** * *::: *. :*:: :*	
Drosophila melanogaster; AAA28406.2	kciddldfyvyryqsygktfikscqvspdvyiqlalqlahyk------------------	521
Culex quinquefasciatus; EDS31208.1	rsiedldfyvyrykpfgknfikacqvspdvfiqlalqlayfklvpvetlphvesqpglle	458
Aedes aegypti; XP_021713002.1	kniedldfyvyryrpfgknfikacqvspdvfiqlalqlayfk------------------	431
	: *:*********: :** ***:*******:********::*	
Drosophila melanogaster; AAA28406.2	--	521
Culex quinquefasciatus; EDS31208.1	krrsggstmtscplpscpvpdsanrfqhnpitltlprqtftlncpqhkhkhaplsircqn	518
Aedes aegypti; XP_021713002.1	--	431

Drosophila melanogaster; AAA28406.2	-----------------lygrlvatyesastrrflhgrvdciraastealewakamcqge	564
Culex quinquefasciatus; EDS31208.1	qvhasvrnslfrvivtrlyghlvstyesastrrfmlgrvdcirsassealewakamcqge	578
Aedes aegypti; XP_021713002.1	-----------------lygylvstyesastrrfllgrvdcirsasmealewakamcqge	474
	*** **:***********: *******:** *************	
Drosophila melanogaster; AAA28406.2	ganvplesdredeeesrkvkfsiyskdhlrelfrcavarqtevmvknilgngidipllgl	624
Culex quinquefasciatus; EDS31208.1	ganvtlesdkeddysae-aandakkrdnlrelfrcaaarqtevmvqnilghgidihllgl	637
Aedes aegypti; XP_021713002.1	ganvtlesdkeedysse--andakkrdhlrelfrcaaarqtevmvqnilghgidihllgl	532
	**** ****:*:: .:. . ..:*:*********.********:****:**** ****	
Drosophila melanogaster; AAA28406.2	reasiev-tgemhelfkdesyiisqcfllstsqvacstdsfmgygpvtprgygcsynphp	683
Culex quinquefasciatus; EDS31208.1	reaskelnqgtvhdlftddcykiancfllstsqvacstnsfmgygpvtphgygasynphp	697
Aedes aegypti; XP_021713002.1	reacre-qqgtlhelftdesykiancfllstsqvacstnsfmgygpvtphgygasynphp	591
	. * * :*:** *:. * *::**********:**********:***.******	
Drosophila melanogaster; AAA28406.2	eqivfcvsafyscedtsasryakslqdsldimrdllqn	721
Culex quinquefasciatus; EDS31208.1	neiifcisaffssdktsasrfarslqdsldamrdlls-	734
Aedes aegypti; XP_021713002.1	neiifcisaffssdktsasrfarslqdsldamrdlls-	628
	::*:**:***:* .:. *****:*:******** *****.	

**Percent Identity Matrix for choline acetyl-transferase**	*Drosophila melanogaster*; AAA28406.2	*Culex quinquefasciatus*; EDS31208.1	*Aedes aegypti*; XP_021713002.1
Drosophila melanogaster; AAA28406.2	100.00	67.66	71.25
Culex quinquefasciatus; EDS31208.1	67.66	100.00	87.26
Aedes aegypti; XP_021713002.1	71.25	87.26	100.00

S1.7. Multiple sequence alignment for glutamine synthetase (Gene ID: 2752)

Homo sapiens; NP_001028216.1	-------------------------------------mttsasshlnkgikqvymslpqg	23
Drosophila melanogaster; AHN59591.1	----------------------------mhsamsariledspnarinktildrylslplq	32
Aedes aegypti; AAV31915.1	---------------------------------msrtleespnahinktlldkymslpmp	27
Culex tarsalis; JAV34836.1	msfrvfgllirqelagvasgkastrmistssvrsarilkdspnaylnktlldryqklkyd	60
Culex quinquefasciatus; EDS39993.1	--	0
Culex pipiens pallens; XP_039453062.1	--	0

Homo sapiens; NP_001028216.1	-ekvqamyiwidgtgeglrcktr-----tldsepkcveelpewnfdgsstlqsegsnsdm	77
Drosophila melanogaster; AHN59591.1	enivqatyvwidgtgedlrckdr-----tldfipqspkelpvwnydgsscyqaegsnsdt	87
Aedes aegypti; AAV31915.1	egkiqatyiwidgtgenlrckdr-----tlefipekpsdlpiwnydgsscyqaeghnsdv	82
Culex tarsalis; JAV34836.1	pkyvqatyiwidgtgenvrckdr-----tldfipekpsdlpiwnydgsscyqaeghnsdv	115
Culex quinquefasciatus; EDS39993.1	----------------mqncreplipntlsysfpaistdlpiwnydgsscyqaeghnsdv	44
Culex pipiens pallens; XP_039453062.1	--	0

Homo sapiens; NP_001028216.1	ylvpaamfrdpfrkdpnklvlcevfkynrrpaetnlrhtckrimdmvsnqhpwfgmeqey	137
Drosophila melanogaster; AHN59591.1	ylypvaiykdpfrrgnnilvmcdtykfdgtptdtnkrktclevankcaaeepwfgieqey	147
Aedes aegypti; AAV31915.1	ylhpvaifkdpmrpgnnilvmcetyrfdgtptqsnkrrtcrevcdkvadqqpwfgieqey	142
Culex tarsalis; JAV34836.1	ylhpvaiykdpfrrgnnilvmcetyrfdgrptesnkrrtcrevcdqvaaekpwfgieqey	175
Culex quinquefasciatus; EDS39993.1	ylhpvaiyrdpfrrgnnilvmcetyrfdgrptesnkrrtcrevcdqvagekpwfgieqey	104
Culex pipiens pallens; XP_039453062.1	--------------------mcetyrfdgrptesnkrrtcrevcdqvagekpwfgieqey	40
	:*:.:::: *:::* *:** .: : : :.****:****	

```
Homo sapiens; NP_001028216.1                  tlmgtdghpfgwpsngfpgpqgpyycgvgadraygrdiveahyraclyagvkiagtnaev   197
Drosophila melanogaster; AHN59591.1           tfldfdghplgwpkngfpgpqgpyycgvgankvyardivdahyraclyagikvsgtnaev   207
Aedes aegypti; AAV31915.1                     tlldidgrplgwpkngfpgpqgpyycgvgankvyardivdahyraclyagikicgtnaev   202
Culex tarsalis; JAV34836.1                    tlldidgrplgwpkngfpgpqgpyycgvgadkvyardivdahyraclyagvkicgtnaev   235
Culex quinquefasciatus; EDS39993.1            tlldidgrplgwpkngfpgpqgpyycgvgadkvyardivdahyraclyagvkicgtnaev   164
Culex pipiens pallens; XP_039453062.1         tlldidgrplgwpkngfpgpqgpyycgvgadkvyardivdahyraclyagvkicgtnaev   100
                                              *::. **:*:***.*****************::.*.****:**********:*:.******

Homo sapiens; NP_001028216.1                  mpaqwefqigpcegismgdhlwvarfilhrvcedfgviatfdpkpipgnwngagchtnfs   257
Drosophila melanogaster; AHN59591.1           mpaqwefqvgpcegisigddlwmarfllhriseefgivstldpkpmpgdwngagahtnvs   267
Aedes aegypti; AAV31915.1                     mpaqweyqvgpcegisigdelwvsrfllhriaeefgivatldpkpmvgdwngagahtnvs   262
Culex tarsalis; JAV34836.1                    mpaqweyqvgpcegismgddlwmsrfllhriaeefgivatldpkpmpgdwngagahtnvs   295
Culex quinquefasciatus; EDS39993.1            mpaqweyqvgpcegismgddlwmsrfllhriaeefgivatldpkpmpgdwngagahtnvs   224
Culex pipiens pallens; XP_039453062.1         mpaqweyqvgpcegismgddlwmsrfllhriaeefgivatldpkpmpgdwngagahtnvs   160
                                              ******:*:********:**.**::**:***::*:**::*:****: *:*****.***.*

Homo sapiens; NP_001028216.1                  tkamreenglkyieeaieklskrhqyhiraydpkggldnarrltgfhetsnindfsagva   317
Drosophila melanogaster; AHN59591.1           tkamredggirdiekavaklskcherhiraydpkqgqdnarrltgkhetssindfsagva   327
Aedes aegypti; AAV31915.1                     tktmreeggiveiesaiaklskcherhiraydpregkdnerrltgkhetssiydfnagva   322
Culex tarsalis; JAV34836.1                    tqamreegglaeiekaiaklsqcherhiraydprggkdnerrltgkhetssihdfnagva   355
Culex quinquefasciatus; EDS39993.1            tqamreegglaeiekaiaklstcherhiraydprggkdnerrltgkhetssihdfnagva   284
Culex pipiens pallens; XP_039453062.1         tqamrdegglaeiekaiaklstcherhiraydprggkdnerrltgkhetssihdfnagva   220
                                              *::**::*:   **.*: ***   *: *******: * ** ***** ****.* **.****
```

Homo sapiens; NP_001028216.1	nrsasiriprtvgqekkgyfedrrpsancdpfsvtealirtcllnetgdepfqykn	373
Drosophila melanogaster; AHN59591.1	nrgcsiriprgvnddgkgyfedrrpssncdpysvveailrticlde----------	373
Aedes aegypti; AAV31915.1	nrgasvriprgvadegkgyfedrrpssncdpysvveailrticlde----------	368
Culex tarsalis; JAV34836.1	nrgasvriprgvadegkgyfedrrpssncdpyvvceailrticlne----------	401
Culex quinquefasciatus; EDS39993.1	nrgasvriprgvadegkgyfedrrpssncdpyvvceailrticlde----------	330
Culex pipiens pallens; XP_039453062.1	nrgasvriprgvadegkgyfedrrpssncdpyvvceailrticlde---------- 266	
	**.:*:**** * :: **********:****: * **::** *:*	

**Percent Identity Matrix for glutamine synthetase**	*Homo sapiens*; NP_001028216.1	*Drosophila melanogaster*; AHN59591.1	*Aedes aegypti*; AAV31915.1	*Culex tarsalis*; JAV34836.1	*Culex quinquefasciatus*; EDS39993.1	*Culex pipiens pallens*; XP_039453062.1
Homo sapiens; NP_001028216.1	100.00	65.29	65.01	65.56	66.15	69.55
Drosophila melanogaster; AHN59591.1	65.29	100.00	81.79	80.97	80.00	83.08
Aedes aegypti; AAV31915.1	65.01	81.79	100.00	87.77	87.38	91.35
Culex tarsalis; JAV34836.1	65.56	80.97	87.77	100.00	94.15	98.50
Culex quinquefasciatus; EDS39993.1	66.15	80.00	87.38	94.15	100.00	99.62
Culex pipiens pallens; XP_039453062.1	69.55	83.08	91.35	98.50	99.62	100.00

S1.8. Multiple sequence alignment for tyrosine-hydroxylase (Gene ID: 25085)

Rattus norvegicus; XP_038957248.1	mptpsapspqpk-------gfrravseqdak-------qaeavtvrricchpesprfigr	46
Drosophila melanogaster; CAA53802.1	mmavaaaqknremfaikks-----------------------------ys--iengypsr	29
Culex quinquefasciatus; XP_038115090.1	------meaklnlfsvlktifkmhte------------pqqviqillnce--eengypsr	40
Culex pipiens pallens; XP_039429428.1	------meaklnlfsvlktifkmhte------------pqqviqillnce--eengypsr	40
Aedes aegypti; EJY57397.1	--	0
Anopheles darlingi; ETN61516.1	mmavaaaqknremfaikk-sysieikkrdnrcvlscvalsegdgvvldec--tsngypsr	57
Anopheles merus; XP_041769716.1	mmavaaaqknremfaikk-sysie------------------------------ngypsr	29
Anopheles sinensis; AMZ03511.1	mmavaaaqknremfaikk-sysie------------------------------ngypsr	29

Rattus norvegicus; XP_038957248.1	rqsliedarkereaaaa---	63
Drosophila melanogaster; CAA53802.1	rrslvddarfetlvvkqtkqtvleearskan-----------------------------	60
Culex quinquefasciatus; XP_038115090.1	rrslvddarfetivvkqtkqtvldearvkandsdslevq-----------qvpdqhp---	86
Culex pipiens pallens; XP_039429428.1	rrslvddarfetivvkqtkqtvldearvkandsdslevq-----------qvpdqhp---	86
Aedes aegypti; EJY57397.1	---medavp-------	6
Anopheles darlingi; ETN61516.1	rrslvddarfetivvkqtkqtvldearakandssvestvlqaqeq-qqedklsqeiqqtv	116
Anopheles merus; XP_041769716.1	rrslvddarfetivvkqtkqtvldearakandsslectilqaqeqhqqedkipqevqqtv	89
Anopheles sinensis; AMZ03511.1	rrslvddarfetivvkqtkqtvldearakan-----------------edklpqevqqtv	72

Rattus norvegicus; XP_038957248.1	---------------------------aaaaavassepgnpleavvfee-------rdgn	89
Drosophila melanogaster; CAA53802.1	------------------------------------dygltedeillanaasessdaeaa	84
Culex quinquefasciatus; XP_038115090.1	------------qavddlpydpeeiv-sngaqddetdaglteeevvlqnaasespeaeke	133
Culex pipiens pallens; XP_039429428.1	------------qavddlpydpeeiv-sngaqddetdaglteeevvlqnaasespeaeke	133
Aedes aegypti; EJY57397.1	-e--------qqlqadev---pqmeenqatsqdeiddaglteeevvlqnaasespeaekq	54
Anopheles darlingi; ETN61516.1	edndydeeeirmaavdslphkpqehepsaddeekekdaglteeevvlqnaasespeaeke	176
Anopheles merus; XP_041769716.1	edqnddeeeirmvavdelpqkpqehvpsaddedketdaglteeevvlqnaasespeaeke	149
Anopheles sinensis; AMZ03511.1	edqdndeeeirmvavddlpqkpeehvpsandedketdaglteeevvlqnaasespeaeke	132
	: * : ::: : :	
Rattus norvegicus; XP_038957248.1	avlnllfslrgtkpsslsravkvfetfeakihhletrpaqrplagsphleyfvrfevpsg	149
Drosophila melanogaster; CAA53802.1	mqsaalvvrlkegisslgrilkaietfhgtvqhvesrqsrve---gvdhdvlikldmtrg	141
Culex quinquefasciatus; XP_038115090.1	vvraavvvhlrdgmgslgrilkavetfhgtvvhlesrqsrsd---gvmfdvlikvdmara	190
Culex pipiens pallens; XP_039429428.1	vvraavvvhlrdgmgslgrilkavetfhgtvvhlesrqsrsd---gvmfdvlikvdmara	190
Aedes aegypti; EJY57397.1	vvraavvvhlrdgmgslgrilkavetyhgtvvhlesrqsrse---gvqfdvlikvdmars	111
Anopheles darlingi; ETN61516.1	vvraavvvrlrdgmgslgrilkaveayhgvvvhlesrqsrsd---gvqfdvlikidmara	233
Anopheles merus; XP_041769716.1	vvraavvvrlrdgmgslgrilkaveayhgtvvhlesrqsrse---gvqfdvlvkvdmara	206
Anopheles sinensis; AMZ03511.1	vvraavvvrlrdgmgslgrilkavegyhgtvvhlesrqsrse---gvafdvlikvdmars	189
	:. .**.* :*..* :.. : *:*:* :: . : ::.:: .	

Rattus norvegicus; XP_038957248.1	dlaallssvrrvsdd-------vrsaredkvpwfprkvseldkchhlvtkfdpdldldhp	202
Drosophila melanogaster; CAA53802.1	nllqlirslrqsgsfssmnlmadn-nlnvkapwfpkhaseldncnhlmtkyepdldmnhp	200
Culex quinquefasciatus; XP_038115090.1	nllqlirslrqtasfgsvavlaenntvnvkapwfpkhaseldncnhlmtkyepdldmnhp	250
Culex pipiens pallens; XP_039429428.1	nllqlirslrqtasfgsvavlaenntvnvkapwfpkhaseldncnhlmtkyepdldmnhp	250
Aedes aegypti; EJY57397.1	nllqlirslrqtasfgsvsllaen-nvsvkapwfpshaseldncnhlmtkyepdldmnhp	170
Anopheles darlingi; ETN61516.1	nllqlirslrqtqsfgsvsllsen-nvnvkapwfpkhaseldncnhlmtkyepdldmnhp	292
Anopheles merus; XP_041769716.1	nllqlirslrqtqsfgsvsllsen-nvnvkapwfpkhaseldncnhlmtkyepdldmnhp	265
Anopheles sinensis; AMZ03511.1	nllqlirslrqtqsfgsvsllsen-nvnvkapwfpkhaseldncnhlmtkyepdldmnhp	248
	:* *: *:*: . . . *.**** .:****:*:**:**::****::**	

Rattus norvegicus; XP_038957248.1	gfsdqvyrqrrkliaeiafqykhgepiphveytaeeiatwkevyvtlkglyathacrehl	262
Drosophila melanogaster; CAA53802.1	gfadkvyrqrrkeiaeiafaykygdpipfidysdvevktwrsvfktvqdlapkhacaeyr	260
Culex quinquefasciatus; XP_038115090.1	gfadqvyrarrkeiaeiafsykygdpiphinytetenktwsavfarvkdlmqkhacseyv	310
Culex pipiens pallens; XP_039429428.1	gfadqvyrarrkeiaeiafsykygdpiphinytetenktwsavfarvkdlmqkhacseyv	310
Aedes aegypti; EJY57397.1	gfadqvyrarrkeiaeiafaykygdpiphinytetenktwsavfnrvkelmtkhacseyv	230
Anopheles darlingi; ETN61516.1	gfadqvyrarrkeiaeiafayrygdpiphidytetenktwaavfgrvkelmvkhacseyi	352
Anopheles merus; XP_041769716.1	gfadqvyrarrkeiaeiafayrygdpiphidytetenktwaavfgrvkelmvqhacseyi	325
Anopheles sinensis; AMZ03511.1	gfadqvyrarrkeiaeiafayrygdpiphinytetenktwsavftrvkelmvkhacseyi	308
	**:*:*** *** ****** *::*:***.::*: * ** *: :: * *** *:	

Rattus norvegicus; XP_038957248.1	egfqllerycgyredsipqledvsrflkertgfqlrpvagllsardflaslafrvfqctq	322
Drosophila melanogaster; CAA53802.1	aafqklqdeqifvetrlpqlqemsdflrkntgfslrpaaglltardflaslafrifqstq	320
Culex quinquefasciatus; XP_038115090.1	avfhkleeekifvngripqlqemsdflrkntgftlrpaaglltardflaslafrifqstq	370
Culex pipiens pallens; XP_039429428.1	avfhkleeekifvngripqlqemsdflrkntgftlrpaaglltardflaslafrifqstq	370
Aedes aegypti; EJY57397.1	avfkkledekifvkerlpqlqemsdflrkntgftlrpaaglltardflaslafrifqstq	290
Anopheles darlingi; ETN61516.1	avfrkleeekifvkerlpqlqemsdflrkntgftlrpaaglltardflaslafrifqstq	412
Anopheles merus; XP_041769716.1	avfrkledekifvkerlpqlqemsdflrkntgftlrpaaglltardflaslafrifqstq	385
Anopheles sinensis; AMZ03511.1	avfkkleeekifvnerlpqlqemsdflrrntgftlrpaaglltardflaslafrifqstq	368
	*: *: : : :***:::* **:..*** ***.****:***********:**.**	

Rattus norvegicus; XP_038957248.1	yirhasspmhspepdcchellghvpmladrtfaqfsqdiglaslgasdeeieklstvywf	382
Drosophila melanogaster; CAA53802.1	yvrhvnspyhtpepdsihellghmplladpsfaqfsqeiglaslgasdeeieklstvywf	380
Culex quinquefasciatus; XP_038115090.1	yvrhinspyhtpepdcihellghmplladpsfaqfsqeiglaslgasdeeieklstvywf	430
Culex pipiens pallens; XP_039429428.1	yvrhinspyhtpepdcihellghmplladpsfaqfsqeiglaslgasdeeieklstvywf	430
Aedes aegypti; EJY57397.1	yvrhinspyhtpepdcihellghmplladpsfaqfsqeiglaslgasdeeieklstvywf	350
Anopheles darlingi; ETN61516.1	yvrhinspyhtpepdcihellghmplladpsfaqfsqeiglaslgasdeeieklstvywf	472
Anopheles merus; XP_041769716.1	yvrhinspyhtpepdcihellghmplladpsfaqfsqeiglaslgasdeeieklstvywf	445
Anopheles sinensis; AMZ03511.1	yvrhinspyhtpepdcihellghmplladpsfaqfsqeiglaslgasdeeieklstvywf	428
	*:** .** *:****. ******:*:*** :******:**********************	

Rattus norvegicus; XP_038957248.1	tvefglckqngelkaygagllssygellhslseepevrafdpdtaavqpyqdqtyqpvyf	442
Drosophila melanogaster; CAA53802.1	tvefglckehgqikaygagllssygellhaisdkcehrafepastavqpyqdqeyqpiyy	440
Culex quinquefasciatus; XP_038115090.1	tvefglckennevkaygagllsaygellhaisdkpehrafepastavqpyqdqeyqpiyy	490
Culex pipiens pallens; XP_039429428.1	tvefglckennevkaygagllsaygellhaisdkpehrafepastavqpyqdqeyqpiyy	490
Aedes aegypti; EJY57397.1	tvefglckennevkaygagllsaygellhaisdkpehrafdpastavqpyqdqeyqpiyy	410
Anopheles darlingi; ETN61516.1	tvefglckekdevkaygagllsaygellhaisdkpehrafdpastavqpyqdqeyqpiyy	532
Anopheles merus; XP_041769716.1	tvefglckekdevkaygagllsaygellhaisdkpehrpfepastavqpyqdqeyqpiyy	505
Anopheles sinensis; AMZ03511.1	tvefglckeknevkaygagllsaygellhaisdkpehrpfdpastavqpyqdqeyqpiyy	488
	********::.:::*********:******::*:: * * *:* ::******** ***:*:	

Rattus norvegicus; XP_038957248.1	vsesfndakdklrnyasriqrpfsvkfdpytlaidvldsphtiqrslegvqdelhtlaha	502
Drosophila melanogaster; CAA53802.1	vaesfedakdkfrrwvstmsrpfevrfnphtervevldsvdkletlvhqmnteilhltna	500
Culex quinquefasciatus; XP_038115090.1	vaesfedakekfrrwvstmsrpfevrfnphtervevldsvdkldtlvsqlnteilhltna	550
Culex pipiens pallens; XP_039429428.1	vaesfedakekfrrwvstmsrpfevrfnphtervevldsvdkldtlvsqlnteilhltna	550
Aedes aegypti; EJY57397.1	vaesfedakekfrrwvstmsrpfevrfnphtervevldsvekldtlvsqlnteilhltna	470
Anopheles darlingi; ETN61516.1	vaesfedakekfrrwvstmsrpfevrfnphtervevldsvdkletlvsqlntemlhltna	592
Anopheles merus; XP_041769716.1	vaesfedakekfrrwvstmsrpfevrfnphtervevldsvdkletlvsqlntevlhltna	565
Anopheles sinensis; AMZ03511.1	vaesfedakekfrrwvstmsrpfevrfnphtervevldsvdkldtlvsqlntellhltna	548
	*:***:***:*:*.::.* .:*** *:*:*:* ::**** ..:: : :: *: *::*	

Rattus norvegicus; XP_038957248.1	lsais----	507
Drosophila melanogaster; CAA53802.1	isklrrpf-	508
Culex quinquefasciatus; XP_038115090.1	iaklrqpfc	559
Culex pipiens pallens; XP_039429428.1	iaklrqpfc	559
Aedes aegypti; EJY57397.1	iaklrqpfc	479
Anopheles darlingi; ETN61516.1	iaklrqpfc	601
Anopheles merus; XP_041769716.1	iaklkqpfc	574
Anopheles sinensis; AMZ03511.1	ieklrqpfc	557
	: :	

**Percent Identity Matrix for tyrosine-hydroxylase**	*Rattus norvegicus*; XP_038957248.1	*Drosophila melanogaster*; CAA53802.1	*Culex quinquefasciatus*; XP_038115090.1	*Culex pipiens pallens*; XP_039429428.1	*Aedes aegypti*; EJY57397.1	*Anopheles darlingi*; ETN61516.1	*Anopheles merus*; XP_041769716.1	*Anopheles sinensis*; AMZ03511.1
Rattus norvegicus; XP_038957248.1	100.00	49.57	48.68	48.68	51.59	47.70	48.54	48.75
Drosophila melanogaster; CAA53802.1	49.57	100.00	80.88	80.88	82.37	82.84	83.50	83.10
Culex quinquefasciatus; XP_038115090.1	48.68	80.88	100.00	100.00	91.95	84.56	87.06	89.31
Culex pipiens pallens; XP_039429428.1	48.68	80.88	100.00	100.00	91.95	84.56	87.06	89.31
Aedes aegypti; EJY57397.1	51.59	82.37	91.95	91.95	100.00	90.19	90.40	91.21
Anopheles darlingi; ETN61516.1	47.70	82.84	84.56	84.56	90.19	100.00	95.64	94.79
Anopheles merus; XP_041769716.1	48.54	83.50	87.06	87.06	90.40	95.64	100.00	95.69
Anopheles sinensis; AMZ03511.1	48.75	83.10	89.31	89.31	91.21	94.79	95.69	100.00
